# Adoption of Standard Reference SNP Identifiers in Agricultural Genomics for Interoperability and Data Reuse

**DOI:** 10.1038/s41597-026-07208-0

**Published:** 2026-04-16

**Authors:** Marcela K. Tello-Ruiz, Timothee Cezard, Carson Andorf, Sonia Balyan, Nahla V. Bassil, Sebastian Beier, Jill M. Bushakra, Tao-Ho Chang, Kapeel Chogule, Irene Cobo-Simón, Sarah Dyer, Christine G. Elsik, Nicholas Gladman, Melanie Harrison, Jodi Humann, Catherine Kim, Vivek Kumar, Raja S. Nandety, Rex Nelson, Andrew Olson, Taner Z. Sen, Moira J. Sheehan, Sharon Wei, Doreen Ware

**Affiliations:** 1 Cold Spring Harbor Laboratory, Cold Spring Harbor, NY 11724 USA; 2 European Molecular Biology Laboratory, European Bioinformatics Institute (EMBL-EBI), Wellcome Genome Campus, Hinxton, Cambridge CB10 1SD UK; 3 Department of Computer Science, Iowa State University, Ames, IA 50011 USA; 4 United States Department of Agriculture, Agricultural Research Service (USDA-ARS), Corn Insects and Crop Genetics Research Unit, Ames, IA 50011 USA; 5 Indian Biological Data Centre, Regional Centre for Biotechnology, Faridabad, Haryana 121001 India; 6 USDA ARS National Clonal Germplasm Repository, Corvallis, OR 97333 USA; 7 Institute of Bio- and Geosciences (IBG-4 Bioinformatics), CEPLAS, BIOSC, Forschungszentrum Jülich GmbH, Wilhelm Johnen Straße, Jülich, Germany; 8 Program in Plant Health Care, Academy of Circular Economy, National Chung Hsing University, Taichung, Taiwan; 9 Institute of Forest Science, National Center National Institute for Agricultural and Food Research and Technology, Spanish National Research Council (ICIFOR-INIA-CSIC), Madrid, Spain; 10 Division of Animal Sciences, University of Missouri, Columbia, MO 65211 USA; 11 USDA ARS NEA, Plant Soil & Nutrition Laboratory Research Unit, Ithaca, NY 14853 USA; 12 USDA ARS, Plant Genetic Resources Conservation Unit, Griffin, GA 30223 USA; 13 Department of Horticulture, Washington State University, Pullman, WA 99164 USA; 14 USDA ARS PA, Edward T. Schafer Agricultural Research Center, Fargo, ND 58102 USA; 15 USDA ARS, Crop Improvement and Genetics Research, Albany, CA 94710 USA; 16 Department of Plant Breeding and Genetics, Cornell University, Ithaca, NY 14850 USA; 17 Breeding Insight, University of Florida - IFAS, Gainesville, FL 32611 USA; 18 Present Address: Phoenix Bioinformatics, 39899 Balentine Dr, Ste 200, Newark, CA 94560 USA

**Keywords:** Genetic databases, Agricultural genetics

## Abstract

Agricultural research has long faced challenges with data sharing, often relying on informal networks and requiring significant effort to clean and harmonize data. This hampers collaboration and limits data reuse. While FAIR (Findable, Accessible, Interoperable, and Reusable) principles are widely adopted in biomedical research, their uptake in agricultural genomics has lagged. The AgBioData Standards for Genetic Variation Working Group aims to close this gap by promoting FAIR data practices. We surveyed current standards for managing agricultural genetic variation and recommend adopting reference SNP identifiers (rsIDs) as a key step. We present examples from crop research communities with varying data maturity, including those without reference assemblies. Milestones include introducing nearly 220 million rsIDs to Gramene and pangenome databases, projecting rsIDs from reference to pangenome varieties in sorghum and maize, and developing an agricultural FAIR guide for rsID adoption. Better coordination among data producers, repositories, and breeding platforms is essential to improve interoperability, consistency, and accelerate genetic variant discovery for crop trait improvement.

## Introduction

Genetic variation underpins evolution and the improvement of crops and livestock, providing the raw material for selection and adaptation in both natural ecosystems and breeding programs. As the agricultural sector increasingly turns to genomics-assisted breeding to meet global food security challenges, the ability to effectively reuse and integrate genetic variation data across studies and platforms has become essential. The FAIR principles of data management promote standardization through the use of shared identifiers, file formats, and metadata schemas^1^. However, agricultural genomics still lags behind human genetics in adopting FAIR practices. Fragmented workflows, inconsistent standards, and limited cross-platform interoperability have led to siloed and poorly annotated datasets, impeding collaboration, reproducibility, and downstream breeding applications.

FAIRification, the process of applying FAIR principles, is particularly challenging in agriculture due to the diversity of species, data types, research goals, and community practices. To address these barriers, the AgBioData Consortium^2^ formed the Standards for Genetic Variation Working Group (SGV WG), bringing together geneticists, breeders, bioinformaticians, and biocurators to improve the interoperability and reusability of genetic variation data. Descriptions of key data standards for biosamples, phenotypic traits, and genetic markers, in the context of FAIR management best practices are provided in Supplementary Information section 1.

A major obstacle in the FAIRification of agricultural data has been the limited and inconsistent use of globally recognized identifiers, particularly reference SNP cluster IDs (rsID, singular; rsIDs plural), for genetic markers. Introduced in 1998 by dbSNP^3^, rsIDs are globally unique, assembly-independent identifiers that cluster genetic variants observed at the same genomic locus. They enable consistent referencing of variants across assemblies, databases, and studies, unlocking compatibility with a wide range of bioinformatics tools and analysis pipelines^4^. rsIDs are widely adopted in human genomics, where they form the backbone of genome-wide association studies (GWAS), clinical variant interpretation, and large-scale meta-analyses^5–7^. Their adoption in agriculture, however, has been slow.

**Fig. 1:**
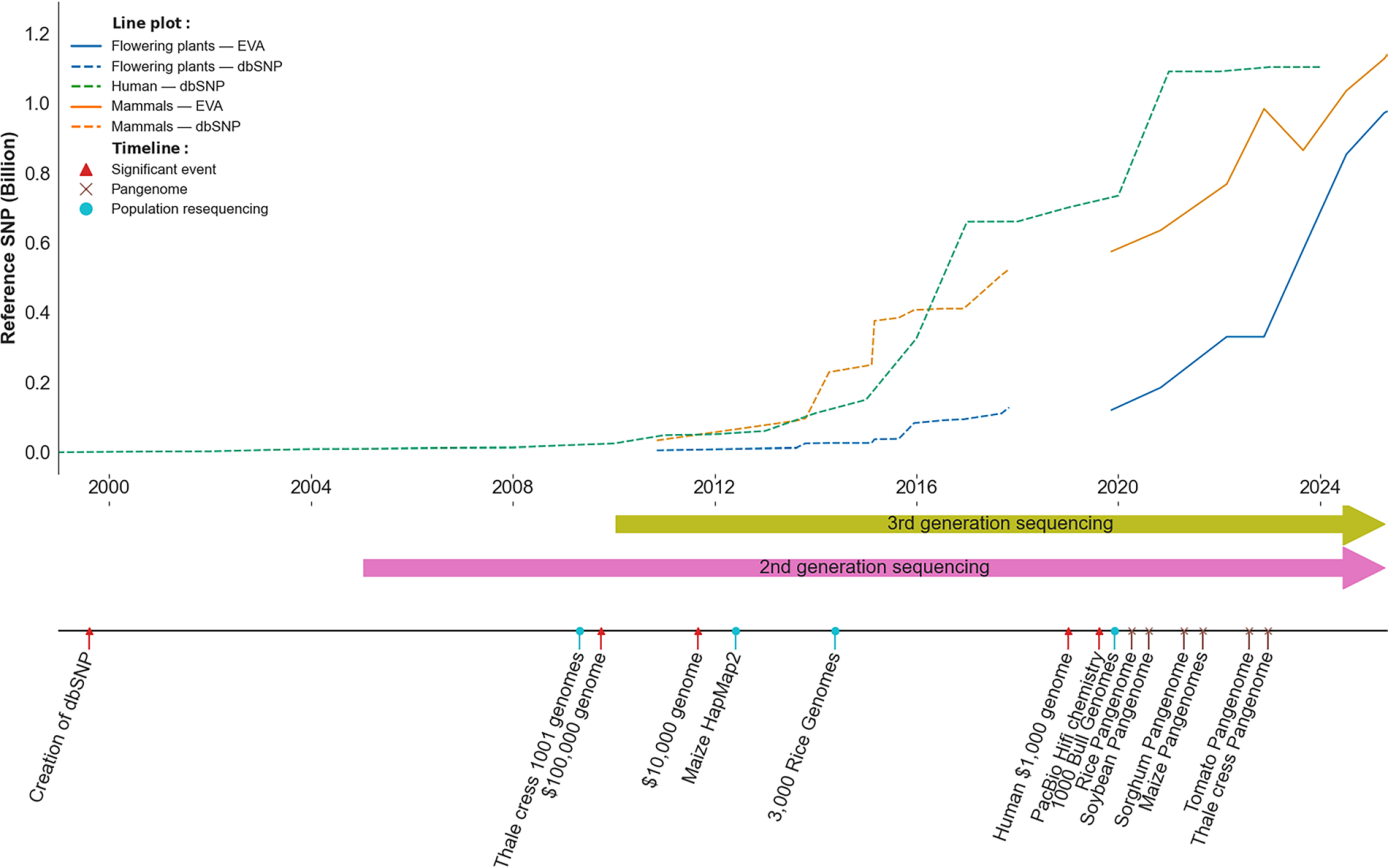
Timeline of rsID availability alongside major sequencing and genomics milestones. The graph shows the number of rsIDs released by dbSNP and EVA over time for humans, mammals or flowering plants; the gap marks the transition of non-human variant curation from dbSNP to EVA between 2018–2020. The timeline at the bottom indicates key milestones and developments in the adoption of rsIDs.

This delay stems from key differences between agricultural and human genomics. While human genetics focuses on a single species and benefits from centralized funding and infrastructure, agricultural genetics spans numerous species, each with its own research community, data ecosystem, and breeding priorities. dbSNP initially assigned rsIDs to multiple species but discontinued non-human support in 2017. The European Variation Archive^8^ (EVA, https://www.ebi.ac.uk/eva) assumed this role, becoming the largest short genetic variant database^9^. It currently holds over 3.4 billion variants across 288 species, with the integration of agricultural species gaining momentum in recent years (Fig. 1).

Another limiting factor has been the lack of high-quality, International Nucleotide Sequence Database Collaboration^10^ (INSDC)-accessioned reference genomes in many crop species, a prerequisite for assigning rsIDs by the EVA. Historically, agricultural genomics communities relied heavily on internal IDs or project-specific marker names, often without consistent metadata or shared naming conventions, making it difficult to harmonize genetic variant data files or integrate data across studies. Breeding programs further prioritized short-term deployment over long-term data interoperability, contributing to fragmented data ecosystems.

Recent developments are beginning to shift this landscape. A growing number of high-quality plant and animal reference genomes have now been accessioned in the INSDC databases (https://www.insdc.org), meeting a key requirement for rsID assignment. Figure 1 outlines technology shifts (e.g., from short- to long-read sequencing) that have supported the development of non-human genomes, as well as key milestones in the evolution of rsID use, illustrating both the early success of human genomics and their delayed, but accelerating availability in agriculture. While rsIDs were introduced in 1998 and have been widely adopted in human genomics for over two decades, the scalable support for non-human organisms by EVA only began in recent years, leaving a multi-year gap in persistent identification for agricultural datasets. This delay underscores the historical fragmentation in agricultural genomics and the recent efforts to bridge these gaps.

Here, we describe coordinated, community-led efforts to close this gap, including practical guidance on data standardization, submission pipelines, and pioneering implementations that demonstrate the feasibility and benefits of large-scale rsID adoption in agriculture. rsIDs enable consistent tracking, comparative analyses, and long-term data sustainability. Embedding rsIDs in pangenome browsers, breeding databases, and annotation pipelines enables critical linkages between genotype and phenotype, powering more effective association studies and trait discovery. We are now at a tipping point, driven by improved reference genome availability, mature submission workflows, and increased community engagement. This article highlights the strategic importance of rsID adoption for agricultural research and proposes a roadmap for advancing standardization and interoperability. We call on researchers, professional societies, journals, funders, and data stewards to join in this effort to unlock the full potential of genetic variation data for crop and livestock improvement.

## Results

### Challenges for genetic variation data standardization

One of our primary targets has been to support the harmonization and adoption of standards for genetic variation data, and to promote interoperability and access to agricultural datasets. After surveying the agricultural research community on existing and anticipated genetic variation data sets, accessibility, data handling practices, interoperability, and usability, we identified challenges and proposed initiatives to FAIRify the data. Table S1 (see supplementary xlsx file) provides an overview of the main challenges identified over a three-year period through surveys, panel presentations, and breakout room discussions at focused workshops with other members of the AgBioData community. Chief among them was the need to promote the widespread adoption of standard rsIDs among agricultural communities.

### Applications of standard rsIDs in agricultural research

**Fig. 2:**
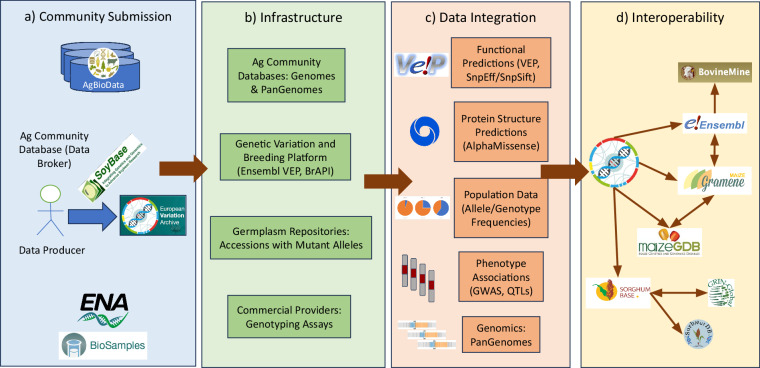
The agricultural genetic variation data journey enabled by rsID adoption. The diagram illustrates the lifecycle of standardized variant data across four stages: (**a**) Community Submission: Data producers submit variant datasets to agricultural community databases (acting as data brokers, e.g., SoyBase), which facilitate validation and submission to the European Variation Archive (EVA) for the assignment of persistent rsIDs. (**b**) Infrastructure: This standardized data is ingested into a foundational infrastructure, including community genome databases, pangenome collections, and commercial genotyping assays. (**c**) Data Integration: rsIDs serve as stable anchors for downstream analysis, enabling the integration of variants with functional predictions (VEP, SnpEff), protein structure models (AlphaMissense), population allele frequencies, and phenotypic associations (GWAS/QTLs). (**d**) Interoperability: The final stage represents a network where rsIDs link distinct bioinformatic resources (e.g., Ensembl, Gramene, MaizeGDB, BovineMine) with germplasm repositories (e.g., GRIN-Global, SorbMutDB), allowing researchers to trace specific variants from genomic data directly to physical seed accessions.

Standardized rsIDs enable consistent tracking and identification of specific SNPs across studies, platforms, and databases, with important applications in agricultural research. Genetic variation data undergoes a complex, multi-step life cycle, which herein we refer to as the data journey (Fig. 2). This journey progresses from initial variant detection and submission to centralized archives like the EVA, to rsID assignment and downstream applications such as integration into community databases, pangenome projections, and the development of marker assays. In the following sections, we describe the practical implementation of this framework by agricultural community databases, highlighting the specific results achieved and the FAIR-compliant resources developed at each stage of the data journey (Table 1 and Fig. 2).

**Table 1:** Summary of Results and Resources Advancing rsID Adoption and Interoperability in Agricultural Genomics.

Category	Main Objective	Key Actions	Results and Resources
Submission	Support community submissions of curated SNP datasets to EVA	• Identified key studies in sorghum and soybean • SorghumBase and SoyBase contacted lead PIs to promote the importance of rsID adoption • EVA and SoyBase provided submission and brokering support to under-resourced research groups	**• Result**: Sorghum rsIDs in EVA increased from 8 M to 55 M • **Result**: Soybean rsIDs in EVA increased from 18 M to 28 M • **Resource**: EVA submission guideline for agricultural species
Database adoption	Integrate rsIDs with traits, germplasm, and functional predictions	• Replaced custom IDs with rsIDs in community databases (*e.g*., MaizeGDB, SorghumBase, and SoyBase) • Used Ensembl VEP for variant annotation • Aligned sample metadata with available genotyping information	• **Result**:220 M rsIDs adopted across 5 species in Gramene pangenome databases • **Result**: Integrated GWAS/QTLs with germplasm and phenotypes • **Resource**: Enabled seed ordering via GRIN and other repositories
Pangenome projection	Ensure consistency across agricultural pangenomes for variant tracking	• Benchmarking with EBI remapping pipeline • SorghumBase projected rsIDs across sorghum cultivars • Gramene Maize applied approach in maize cultivars	**• Result**: Validated projection accuracy using functional variants (e.g., *Su1*) in sweet corn • **Resource**: Expanded pangenome tools in Gramene Maize
Commercial arrays	Extend rsIDs usage in commercial marker panels	• Developed streamlined rsIDs assignment protocol with EVA • Collaborated with genotyping service providers to foster rsID adoption • Matched array markers to rsIDs	• **Resource**: Assigned rsIDs to 2,421 SCMP and 3,490 DArTag markers • **Resource**: Shared rsIDs lists with 12 providers
Inter- operability	Enable cross-resource integration of variant data	• Integrating EVA rsIDs with Ensembl, BovineMine, MaizeGDB, Gramene, etc. • Linking variants with accessions in germplasm repositories	• **Resource**: Indexed accessions with putative loss-of-function (pLoF) variants across repositories (i.e., variants in Gramene databases linked to germplasm accessions in GRIN, IRRI, MaizeGDB, SorbMutDB) • **Resource**: Created “Germplasm” tab for seed ordering associated with pLoFs in Gramene’s search interface

#### Brokering the submission of community-generated variant datasets to the EVA

To quantify the impact of active curation on data availability, we brokered the submission of key datasets from the sorghum and soybean communities to the EVA (Supplementary Information section 2). In sorghum, the submission of three major studies^11–13^ increased the number of rsIDs available to the community from 8 million in EVA release 3 (February 2022) to over 55 million in release 7 (April 2025). Similarly, SoyBase^14^ (https://www.soybase.org) curators submitted over 30 million soybean SNPs to the EVA on behalf of data generators from three selected studies^15–17^ aiming at broadly representing genomic variation across the species, raising the number of assigned rsIDs from 18 million to over 28 million. For communities such as raspberry and blackberry, where the lack of an INSDC-accessioned reference genome prevented immediate archiving, we established a support workflow to guide principal investigators through genome submission. This intervention initiates the prerequisite steps for future rsID generation in under-resourced crops. These results demonstrate that targeted brokering effectively populates central archives, creating a foundation of standardized identifiers that can be reused by the broader community.

#### Agricultural community databases adopt rsIDs to integrate variation data with traits, functional predictions, and population-level data

Submissions to the EVA must include validated VCF files with allele frequency or genotype data, along with metadata describing the samples. Once rsIDs are assigned, these identifiers act as anchors for cross-platform interoperability between the EVA, INSDC databases, and biosample/germplasm repositories, enabling downstream use by various agricultural research platforms (Figure S1, see Supplementary Information document).

Gramene Plants^18^ (https://www.gramene.org) and its associated pangenome sites: SorghumBase^19^ (https://www.sorghumbase.org), Gramene Oryza^20^ (https://oryza.gramene.org), Gramene Maize (https://maize-pangenome.gramene.org), and Gramene Grapevine (https://vitis.gramene.org), pioneered the adoption of standardized rsIDs for genetic variants in five crops and plant model species, leveraging EVA releases 5 and 6. In total, nearly or almost 220 million custom variant IDs were replaced with stable rsIDs: 78.9 million in maize, 67.7 million in rice, 46.4 million in sorghum, 26.3 million in Arabidopsis, and 315,353 in grapevine. rsID adoption enabled consistent variant tracking across assemblies and streamlining germplasm and phenotyping data integration to support marker-assisted breeding. Functional consequences of variants were predicted using the Ensembl Variant Effect Predictor (VEP)^21^ incorporating algorithms such as SIFT^22^. These analyses identified putative truncating variants (PTVs) including putative loss-of-function (pLOF) alleles across species. A key outcome was the implementation of a Germplasm Tab in SorghumBase^19^, Gramene Oryza^20^, and Gramene Maize (Supplementary Information section 3.1). This feature allows users to cross-reference predicted functional variants with the germplasm accessions carrying the corresponding alleles. Researchers can then order seeds directly through instances of the Germplasm Resource Information Network (GRIN)-Global, such as the International Rice Research Institute (IRRI, see https://www.irri.org/genesys-rice#/a/0385b84d-cad1-4067-80fa-16e01984d5a5). Interoperability with other germplasm resources was also implemented between sorghum pLoF variants and SorbMutDB^23^ (https://www.depts.ttu.edu/igcast/SorbMutDB.php), and integrating Gramene Maize variants with MaizeGDB’s SNPVersity^24^. This process revealed critical biocuration challenges, such as inconsistent cultivar spelling and synonyms, underscoring the parallel need for globally unique identifiers for biosamples.

Building on this interoperable framework, SorghumBase replaced temporary IDs, with permanent rsIDs, and integrated these standardized variants with GWAS phenotypes. Sorghum QTLs were further linked to corresponding entries in the OZ Sorghum QTL Atlas^25^ (https://aussorgm.org.au/sorghum-qtl-atlas), representing the third stage of the data journey (Fig. 1c). This integration supports phenotype prediction, and enhances trait-based marker discovery (Figure S2, see Supplementary Information document). Similarly, MaizeGDB^26^ (https://maizegdb.org; see Supplementary Information section 3.2), is in the second stage of the data journey (Fig. 2b), extending its variant-processing pipeline to assign stable rsIDs at the point of data intake, using EVA release 7. By integrating rsIDs with standard germplasm identifiers, MaizeGDB is enabling direct linking to and ordering from major repositories like the USDA-ARS National Clonal Germplasm System (NPGS) through the GRIN-Global database (https://npgsweb.ars-grin.gov/gringlobal/search), the International Maize and Wheat Improvement Center (CIMMYT, https://www.cimmyt.org/work/seed-request), and the Maize Genetics Cooperation Stock Center (https://maizecoopsc.org/about-our-collection). Standardizing phenotypic variation is crucial for integrating it with genetic variant data from GWAS and QTLs. For example, MaizeGDB hosts over 300 traits associated with more than 40,000 genomic positions, curated from three major GWAS datasets^27–29^. Where rsIDs were not initially assigned, they were added for cases where a GWAS SNP mapped to the same position as a variant existing in the EVA release. This integrated data is presented as genome browser tracks and tables on gene model pages.

Other community databases like TreeGenes^30^ and CartograPlant^31^ for forest trees, and GrainGenes^32^ and the T3/Wheat Triticeae Toolbox^33^ for small grain cereals, are in earlier phases of adopting rsIDs and implementing similar practices (Supplementary Information sections 3.3 and 3.4).

Together, these examples illustrate how rsID adoption across agricultural community databases enhances the integration of genetic variation with functional predictions, trait data, and germplasm accessions. This infrastructure forms a critical foundation for reproducible, interoperable, and FAIR-aligned data practices in agricultural research.

#### Pioneering rsIDs assignment across agricultural pangenomes

As more agricultural pangenomes are sequenced, calling variants separately for each cultivar becomes increasingly impractical. A more scalable solution is to project existing rsIDs onto related cultivar assemblies. This approach is especially important for ensuring consistency in functional studies and gene editing applications. For example, in sorghum, the reference genome BTx623 is not commonly used in molecular systems such as CRISPR, whereas the cultivar RTx430 is preferred due to its higher transformation and regeneration efficiency^34–37^. Projecting rsIDs across these genomes ensures that variant data remains usable and interoperable across experimental platforms.

**Fig. 3:**
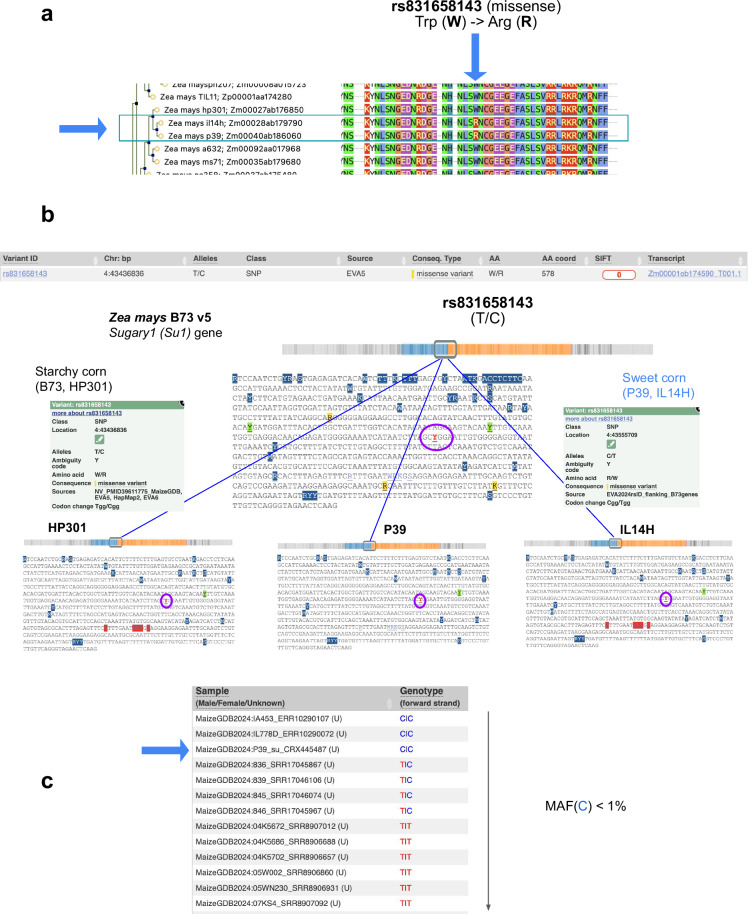
rsID-mediated projection of a functional *Sugary1 (Su1)* variant identifies sweet corn mutants in the Gramene Maize pangenome browser. (**a**) Sweet corn arises from mutations in starch biosynthesis pathway genes, such as *Su1*, that increase sugar content in the endosperm at the expense of starch^62–64^. The missense variant rs831658143 (alleles T/C) is located at amino acid residue 578 towards the 3′ end of a highly conserved N-terminal glycoside hydrolase domain in *Su1*(blue box), which plays a role in converting starchy corn into sweet corn^65^. Shown is a zoomed-in view of the amino acid alignment for the *Su1* gene family tree highlighting the tryptophan-to-arginine substitution at residue 578 (W578R) that was previously reported^62,63,66^. The alternative allele C at this variant locus (blue arrow) is observed in two sweet corn varieties: P39 and IL14H. The gene tree was constructed using 60 plant genomes including 54 distinct maize varieties. (**b**) Flanking sequence context for rs831658143 in the reference maize B73 genome and three other accessions (HP301, P39, and IL14H). Sweet corn varieties carry the alternative allele C encoding arginine, whereas starchy corn varieties retain the reference T allele encoding tryptophan. The variant position is highlighted in red (purple circle), with nearby variants indicated by colored letters and IUPAC ambiguity codes. In the reference B73 genome, this position is homozygous for the T allele. The W-to-R substitution constitutes a missense mutation with a SIFT score of zero, indicating a high likelihood of deleterious impact. (**c**) The Gramene Maize browser displays genotypes for 1,438 accessions at rs831658143, including three homozygous sweet corn varieties (IA453^67^, IL778D, and P39_su), and four heterozygous landraces (835, 836, 839, and 846). This corresponds to a minor allele frequency (MAF) of <1%, indicating that fewer than 1% of accessions bear the C allele^24^.

SorghumBase and Gramene Maize tested this strategy using the EBI Variation variant-remapping pipeline (see Methods) in sorghum^19^ and maize (Fig. 3). According to EBI’s documentation, the pipeline had worked well between different assemblies of the same genome or closely related genomes with SNPs and short Indels, but it had not been tested with larger or more complex variants. This approach was refined in maize, where gene-centric subsets of rsIDs within 1.5 Kb flanking regions of the reference maize B73 gene models were projected onto the genomes of 25 maize genomes from the Nested Association Mapping (NAM) panel and made available as genome tracks in Gramene Maize (see https://maize-pangenome.gramene.org/News?section=Release%205). This method enabled accurate rsIDs mapping within and across genomes of closely related accessions, offering significant potential to accelerate breeding efforts. Of note, MaizeGDB has also started propagating rsIDs across maize pangenomes.

#### Standardizing commercial genotyping arrays facilitates long-term variant data reuse

SNP genotyping arrays are powerful tools widely used in agricultural research and breeding. They support molecular genetics studies by facilitating the discovery of QTLs, GWAS analyses, and inference of kinship and parentage. In genomic and molecular breeding, these arrays facilitate genomic prediction, genomic selection, and marker-assisted selection to improve breeding efficiency. They also play a key role in cultivar development by accelerating the release of new varieties, identifying valuable genetic markers in existing germplasm, and selecting germplasm suited to specific growing environments. Additionally, SNP arrays are critical for intellectual property protection through the verification of clonal purity and confirmation of parent-offspring relationships.

rsIDs allow for better tracking of variants across genome assembly versions by mapping, tracking, merging and deprecating identifiers, which would assist genotyping platform upgrades. In collaboration with Gramene and SorghumBase, the EVA developed a protocol to engage commercial genotyping providers in generating rsIDs for species with incomplete coverage of rsIDs in EVA. Contact was established with 12 genotyping service providers and breeding companies to advocate for the use of rsIDs as a variation identifier in their arrays. The protocol involves reaching out to these providers to explain the benefits of incorporating rsIDs into their workflows and offering a streamlined process through which EVA can assign rsIDs to valid markers not currently covered, without requiring full submission through the archive’s standard procedure (https://www.ebi.ac.uk/eva/?Submit-Data). We applied this protocol to a new Sorghum Community Marker Panel (SCMP), a mid-density array developed by the sorghum research community in collaboration with AgriPlex^38^. The SCMP consists of 2,421 markers that were curated from existing resources including the DArTag genotyping service (https://excellenceinbreeding.org/toolbox/services/sorghum-mid-density-genotyping-services) offered by Diversity Arrays Technology Ltd for the Consultative Group on International Agricultural Research (CGIAR), a community Kompetitive Allele Specific PCR (KASP) panel (https://excellenceinbreeding.org/module3/kasp), quality control markers, and trait-associated markers suggested by breeders. Of the 2,421 SCMP variants, 2,395 were matched to existing rsIDs in EVA release 5. The remaining 26 variants received new rsIDs without having to wait for the next release. All 2,421 rsIDs (Table S2, see supplementary xlsx file) are now included in EVA release 7 for Sorghum bicolor (NCBI assembly GCA_000003195.3). Tailored for the US sorghum community, SCMP is well suited for marker-assisted selection, genetic purity testing and germplasm quality control.

#### Stable identifiers enable resource interoperability

The EVA plays a central role in enabling data integration across resources. For example, Ensembl^39^ (http://www.ensembl.org; see Supplementary Information section 3.5) imports variation data from several public sources, with data for agricultural species primarily coming from the EVA. Its new beta infrastructure at https://beta.ensembl.org (Figure S3, see Supplementary Information document) directly incorporates EVA-submitted datasets, supporting 55 species, 27 of which are agriculturally relevant (12 crops, 10 livestock, and 5 aquaculture), as of release 7. This evolution strengthens the connection between agricultural variant data and Ensembl’s broader annotation and visualization ecosystem, enhancing accessibility and comparative analysis capabilities.

Stable identifiers are essential for connecting genetic, phenotypic, and germplasm data across diverse bioinformatics resources. BovineMine^40^ (https://bovinemine.rnet.missouri.edu; Supplementary Information section 3.6), the data warehouse of the Bovine Genome Database, exemplifies this by integrating single-nucleotide variants and variant effect predictions from Ensembl^39^, with QTL and GWAS data from AnimalQTLdb^41^ (https://www.animalgenome.org/cgi-bin/QTLdb/index), all mapped to the latest bovine genome assembly. Trait associations are annotated using the Vertebrate Trait Ontology^42^, while variants are indexed using rsIDs and include SNP array aliases to support flexible querying. Through InterMine^43^ tools such as QueryBuilder (https://bovinemine.rnet.missouri.edu/bovinemine/customQuery.do) and Genomic Regions search (https://bovinemine.rnet.missouri.edu/bovinemine/genomicRegionSearch.do), users can retrieve, filter, and combine genomic, functional, and trait data, enabling cross-platform integration, reproducible analyses, and customized exploration of genotype-to-phenotype relationships in cattle.

In agricultural systems, interoperability also relies on consistent identifiers for germplasm accessions. Community databases like MaizeGDB, SorghumBase, and Gramene Oryza support cross-referencing with major germplasm repositories using standardized sample identifiers, such as PI and GSOR numbers from GRIN, and IS numbers from the International Crops Research Institute for the Semi-Arid Tropics (ICRISAT, https://genebank.icrisat.org/IND/orderGermplasmDashboard). These identifiers allow researchers and breeders to easily locate and request seeds for accessions of interest.

SorghumBase, Gramene Maize and Gramene Oryza have further extended interoperability by implementing a Germplasm tab within gene search interfaces, linking gene-level variation data with stock center records (Figure S2, see Supplementary Information document). These interfaces index accessions carrying variants predicted by the Ensembl VEP to truncate protein function. A curated metadata layer maps population samples to standardized accession names across stock centers, including GRIN-Global, IRRI, and SorbMutDB, allowing researchers to order both natural accessions and ethyl methanesulfonate (EMS)-induced mutants bearing pLoF alleles.

### FAIR Implementation Guide for Agricultural Genetic Variation Data

We have developed a FAIR Implementation Guide (see Supplementary Information annex) to standardize the sustainable submission, management, and use of variation datasets across diverse agricultural research contexts. This guide addresses critical questions such as how to verify whether existing variants already have assigned rsIDs in the EVA, how to submit new SNP data to EVA, including scenarios where an accessioned reference genome exists for the species in the INSDC, and how to submit one if it does not. It also provides guidance on how to ensure that the variant data is provided in a standardized, validated format such as VCF, including VCF metadata requirements, and the use of globally recognized sample identifiers from major germplasm repositories, such as BioSample or GRIN to ensure traceability and interoperability. The guide enables researchers in adopting the best practices to produce FAIR-compliant variant datasets that are reusable, interoperable and ready for integration in global genomic resources.

We have also advocated for the importance of adopting FAIR standards for genetic variant data, and particularly for the adoption of rsIDs via presentations at major conferences, agricultural workshops, and collaborator meetings. Genetic variation data producers are encouraged to use standard formats like VCF (preferably using rsIDs when available), submitting key SNP data sets to the EVA to obtain rsIDs, and adopting standard sample identifiers associated with a major germplasm repositories like the NPGS, ICRISAT or the Genebank Information System of the Leibniz Institute of Plant Genetics and Crop Plant Research (IPK-GBIS).

## Discussion

SNPs are among the most important forms of genetic variation and are foundational to genomic research, including GWAS, marker-assisted selection, and population diversity analysis. Accurate SNP calling depends on high-quality read mapping, variant calling, and variant filtering, all of which benefit from improved reference genomes and bioinformatics tools.

Historically, many crop species (particularly clonally propagated or polyploid species) lacked high-quality, accessioned reference genomes due to challenges such as genome repetitiveness, polyploidy, and heterozygosity. Recent advances in long-read sequencing technologies^44^ applied to the creation of telomere-to-telomere (T2T) assemblies have helped close longstanding genomic gaps, particularly in repetitive regions such as centromeres and telomeres. This has led to the discovery of novel sequences and previously undetectable SNPs, providing a more complete picture of genetic variation essential for understanding disease resistance, adaptation, and complex trait architecture, which facilitates its use in breeding programs. These technological improvements have also enabled the construction of pangenomes, which offer more accurate placement of short reads and improved variant calling across genetically diverse populations. These enhancements reduce false positives, recover novel SNPs, and ultimately improve the resolution of domestication history and population diversity studies, as demonstrated in humans^45^ and goats^46^.

Despite these advances, agricultural genomics has lagged behind human genomics in adopting standardized variant identifiers and shared formats. Human genetic databases use rsIDs as stable, assembly-independent references to specific variant loci. This consistency enables integration across genome builds, platforms, and publications, and supports large-scale meta-analysis and data reuse. By contrast, agricultural SNP databases have often been siloed and fragmented, leading to inconsistencies and reduced resource interoperability.

The use of rsIDs represents a critical step forward for agricultural genomics. They serve as persistent identifiers that allow researchers to unambiguously refer to the same variant across genome browsers, literature, phenotypic databases, and functional annotations. In human studies, rsIDs are central to GWAS and ClinVar^6^ catalogs, genotyping arrays, and literature indexing tools such as PubMed (https://pubmed.ncbi.nlm.nih.gov) and LitVar^47^. Resources hosting SNP variant data and genotype-to-phenotype (G2P) associations for agricultural species, such as Genome Variation Map^48^, AnimalQTLdb^41^, Agricultural Animal Omics Database (http://animal.omics.pro) and related variation databases^49–52^), and OMIA^53^, are examples of databases that integrate rsIDs. Through our efforts, rsID adoption and implementation has begun to spread to agricultural community resources like SorghumBase and BovineMine, enabling integration of variant data with germplasm resources and trait information. Other knowledgebases focused on variant cataloging for multiple agricultural species and that were not part of our group, like GWAS Atlas^54^ and Animal-SNP Atlas^55^, could greatly benefit from rsID adoption to support integration with agro-functional genomics and breeding pipelines. Despite these benefits, the adoption of rsIDs in newly emerging crops or livestock genomics initiatives remains challenging. Key bottlenecks include the need for coordination among community databases, breeding programs, and germplasm repositories; the availability of a sufficiently mature reference genome accessioned in the INSDC; limited submission capacity and familiarity with EVA workflows in smaller laboratories; and the retrospective harmonization of incompatible, pre-existing SNP naming schemes. Based on our experience, these challenges can be mitigated by seeding variant catalogs from existing genotyping panels or published GWAS datasets, establishing an initial set of ~10K–50 K high-confidence variants to demonstrate value, assessing whether rsIDs already exist in the EVA, and promoting lightweight tools and concise training materials. Even simple liftOver workflows and short submission guides, adapted from community resources such as the ELIXIR FAIR Cookbook (https://faircookbook.elixir-europe.org) and our FAIR User Guide (see Supplementary Information annex), could substantially lower barriers and accelerate rsID adoption in new agricultural genomics efforts.

Most agricultural SNP studies have relied on a single reference genome, but the adoption of pangenomic approaches has revealed previously missed variants and allowed for correction of misassigned SNPs. For example, improved mapping quality in pangenome contexts reduces spurious SNPs and improves detection of variants linked to economically important traits^56^. Some rsIDs initially assigned to orthologous loci can be revised as reference genomes improve, underscoring the value of having flexible but persistent identifiers.

In this study, we demonstrate that the use of standard rsIDs facilitates merging variant datasets across studies, even when using different reference assemblies or genotyping technologies. We also model how rsIDs support integration with GWAS trait data and improve coordination across databases cataloging pLoF variants. The result is a more interoperable, reusable data ecosystem that supports pangenomic comparisons, cross-platform meta-analyses, and more efficient discovery of genotype-phenotype relationships.

Critically, rsIDs enable compatibility with widely used tools for functional prediction (e.g., Ensembl VEP^21^), protein structure modeling (e.g., AlphaMissense^57^), and literature tracking (e.g., LitVar^47^). They also promote long-term dataset sustainability by enabling variant tracking across genome builds and pangenomes. This makes them indispensable for integration across breeding databases (e.g., BreedBase^58^, DeltaBreed^59^), pangenome browsers (e.g., SorghumBase, Gramene), and germplasm repositories (e.g., NPGS, ICRISAT, IPK-GBIS). Also providing an anchor point for integrating multi-omics datasets (e.g., transcriptomics, epigenomics, metabolomics), facilitating systems-level analyses of trait regulation.

The impact of this approach is already evident. Our group’s work facilitated the assignment of standardized rsIDs for 47 million sorghum and 10 million soybean variants in the EVA. To encourage widespread adoption of rsIDs by agricultural communities, we are providing a stepwise FAIR Genetic Variation User Guide (see Supplementary Information annex) according to the maturity level of a community in the data journey. For example, for communities with an INSDC-accessioned reference genome and existing variant datasets, this might be achieved with simple tools such as bcftools annotate or custom scripts to retroactively assign rsIDs, provided the sequence and alleles match known entries in EVA. In sorghum, rsIDs enabled integration of genotypic data with phenotypic traits and germplasm collections, facilitating direct access to seed materials through platforms like GRIN-Global.

The need for rsID adoption is also seen in commercial genotyping platforms. For example, the strawberry community has a 50 K SNP genotyping array^60^ that includes SNPs tied to multiple reference genomes, making it necessary to map SNP identifiers across platforms. The absence of rsIDs limited interoperability and backward compatibility with prior arrays, while incorporating rsIDs into such platforms would greatly enhance their long-term utility and data traceability.

Going forward, adoption of rsIDs must extend to autopolyploid crops such as alfalfa and blueberry, which pose additional challenges for variant representation. Special considerations are also needed for targeted-amplicon approaches (e.g., DArTag) and for integrating rsIDs with microhaplotype-based identifiers to support haplotype-level selection in breeding programs.

Realizing the full potential of rsID standardization across agricultural genomics requires a coordinated and sustained effort across the research community. While the work presented here demonstrates the feasibility and impact of integrating rsIDs in agricultural pangenome resources, widespread adoption will depend on a multi-pronged strategy involving data infrastructure, tool development, policy alignment, and community engagement.

First, data submission pipelines must be strengthened. Researchers are encouraged to submit high-quality, validated VCF files along with complete sample metadata to the EVA, ensuring that associated reference genomes are accessioned in the INSDC^10^, such as ENA, NCBI, or DDBJ. Comprehensive guidance for these submissions is available through the ELIXIR FAIR Cookbook (e.g., https://w3id.org/faircookbook/FCB061) and the FAIR Implementation Guide we developed (see Supplementary Information annex), which emphasize best practices for data standardization and reproducibility.

Second, the ability to propagate rsIDs across multiple genome assemblies is critical for ensuring variant consistency across diverse germplasm, including landraces, breeding lines, and elite cultivars. Tools such as the EVA remapping workflow provide the technical foundation for this process, supporting alignment across the rapidly growing landscape of pangenomes.

Third, community databases and bioinformatics platforms must be updated to integrate rsIDs within both backend systems and user-facing tools. Embedding rsIDs within variant browsers, functional annotation pipelines, and germplasm access portals will enhance traceability, support G2P association studies, and streamline access to seeds and tissue materials linked to functionally relevant variants.

Equally important is policy-level advocacy. Encouraging journals, funding agencies, and breeding programs to recommend or mandate the use of rsIDs in publications and public data releases will help establish consistent norms. Such policy alignment would not only accelerate adoption but also improve data interoperability across disciplines and repositories. Beyond policy mandates, the data management systems employed by agricultural researchers and plant and animal breeders day-to-day currently do not support storage, display, or retrieval of rsIDs. This is in part due to the lack of annotated rsIDs for many crops, but also confounded by the absence of breeder-defined use cases that require or involve rsIDs. Without defined use cases, software developers cannot create visualizations, analytics, or other features in breeding data platforms to incorporate rsIDs. If and when breeders start to see the value of rsIDs in making more data-driven decisions, and concurrent with the more widespread annotation and adoption of rsIDs across crops, those breeders can make defined feature requests to integrate these data standards into their management software platforms and workflows.

Finally, ongoing community training and support are essential to bridging adoption gaps, particularly for under-resourced crop communities. Targeted outreach, technical documentation, and hands-on workshops will help lower barriers to entry and ensure that all researchers, regardless of crop system or region, can participate in and benefit from rsID-based variant tracking. Without coordinated community efforts, particularly bulk submissions of novel variants and standardized practices, the benefits of rsIDs will remain unrealized.

At the same time, challenges remain in ensuring accurate variant calling in highly repetitive regions, achieving representation of under-studied crops, and securing long-term funding for data infrastructure. Addressing these limitations will be key to realizing the full potential of rsID-based frameworks.

We also advocate for development of expert-curated frameworks, similar to the ACMG/AMP guidelines^61^ in human genetics, for interpreting variant causality in crops. These would provide a consistent basis for linking genotypes to agriculturally important traits across species and breeding systems.

In summary, the delayed adoption of rsIDs in agricultural genomics has constrained progress toward data reuse, interoperability, and large-scale integration. But the field is now at a critical inflection point. With infrastructure in place, community momentum building, and successful implementation examples like maize, soybean, and sorghum, the opportunity is ripe to establish rsIDs as a universal standard for agricultural variation. This transition is not simply a technical upgrade, it is a foundational shift that will enable FAIR data principles, accelerate crop improvement, and link decades of genetic research through a shared, reusable framework.

## Methods

Our methodology followed a three-stage process to standardize agricultural genetic variation: (1) assessing actionable community needs to define data standards, (2) developing technical pipelines to replace temporary identifiers in existing databases, and (3) establishing workflows to project these identifiers across pangenomes.

### Community assessment and definition of standards

In order to identify specific barriers to interoperability, we convened a consistent group of agricultural bioinformatics experts including 5-8 major agricultural bioinformatics resources and a broader membership of approximately 35 researchers. We assessed current practices within the wider AgBioData community through anonymous surveys and workshops, gathering insights on the use of different data types, quality control measures, and the adoption of stable identifiers across member resources (Supplementary Information). These assessments identified two primary technical requirements: the need for standardized biosample identifiers and broader adoption of rsIDs. Based on these findings, we consulted with USDA-affiliated scientists and germplasm curators to review and refine recommendations for making agricultural genotyping data FAIR, with a focus on VCF formatting and metadata alignment. Together with a targeted review of published SNP data sets co-authored by members of the AgBioData Consortium, as well as the identification of unpublished SNP datasets and data standardization challenges through community presentations, this effort informed the development of a “Data Journey” model that illustrates stages of rsID adoption. Such adoption was subsequently extended to pangenomes, supported by community-developed tools and templates for INSDC submission and rsID generation, as described below. Workshop outcomes have been described in Table S1 (see supplementary xlsx file), and metadata guidelines are described in the FAIR User Guide to rsID adoption (see Supplementary Information annex).

### Implementation of rsIDs in community databases

To facilitate the retroactive assignment of stable identifiers, we developed a custom Perl script *import_variant_rsid_all.pl* (https://github.com/warelab/gramene-ensembl/blob/e506aaf4001340e91351f0b620dffb9aea25651d/scripts/load-scripts/import_variant_rsid_all.pl) that processes VCF files from the EVA (releases 5 and 6) to update existing community databases (such as Gramene). The workflow involves downloading full rsID sets for target species (https://ftp.ebi.ac.uk/pub/databases/eva/rs_releases/), splitting them by chromosome for parallel processing, and matching them against local database records using genomic position and allele identity. When a match is identified, the script replaces the temporary internal identifier with the standard rsID and archives the original name in a synonym table to maintain backward compatibility; if no match is found, a new variation record is created.

### Projection of rsIDs across agricultural pangenomes

We adapted the EBI variant remapping pipeline (https://github.com/EBIvariation/variant-remapping/blob/master/README.md) to extend rsID consistency across pangenome assemblies by projecting identifiers from a reference genome to target cultivar assemblies. This pipeline uses the reference assembly flanking sequences of the rsID to anchor it to the target assembly, implementing a tiered mapping strategy using increasing flanking sequence sizes (200 bp, 5 Kb and 50 Kb) based on benchmarking results from sorghum. In order to increase the specificity and reduce the computing cost, we partitioned the rsIDs into chromosome-specific subsets, and restricted mapping to the corresponding chromosomes.

## Supplementary information

Supplementary Information

## Data Availability

The remapped rsIDs in the Gramene Maize PanGenome are available in VCF at https://ftp.gramene.org/maize/release-current/variation/rsID_remapping/. The data sources for BovineMine are available at http://bovinegenome.org/bovinemine/dataCategories.do.

## Code availability

Code from the EBI variant remapping pipeline is available at https://github.com/EBIvariation/variant-remapping/tree/master. Complementary custom scripts for rsIDs mapping, including *import_variant_rsid_all.pl*, are available from https://github.com/warelab/gramene-ensembl/blob/e506aaf4001340e91351f0b620dffb9aea25651d/scripts/load-scripts/.
