# Supplementary material for: Adoption of Standard Reference SNP Identifiers in Agricultural Genomics for Interoperability and Data Reuse
Source: Sci Data. 2026 Apr 16;13:885. doi: 10.1038/s41597-026-07208-0 (PMC13260324; doi:10.1038/s41597-026-07208-0)
Supplement: Supplementary file 1 — Supplementary Information [file 41597_2026_7208_MOESM1_ESM.pdf]

Supplementary Information

1. Phenotypic and Genetic variation data types **1**

2. Submitting variants to the European Variation Archive for rsID generation **3**

3. Agricultural resources with genetic variation data that have adopted rsIDs or have implementation plans to adopt rsIDs **5**

4. Other Supplementary Methods **10**

5. Table S1. Challenges and proposed solutions **18**

6. Table S2. Sorghum Community Marker Panel **19**

7. Annex: FAIR User Guide for rsID adoption **76**

1. Phenotypic and Genetic variation data types

1.1. Biosamples

Genetic materials such as seeds, tissues, or DNA that are used for breeding and conservation are referred to as germplasm. Standardized germplasm identifiers are critical for tracing plant and animal accessions across databases and breeding programs. BioSamples is the central metadata hub for the European Bioinformatics Institute (EMBL-EBI) and is used in the backend of many of its resources. As a hub, it links sample information to a wide array of data across different repositories[1]. The European Nucleotide Archive (ENA) has established sample checklists of expected metadata values that list the minimum amount of information required during sample registration for any sequencing data, and to which all samples registered at ENA must conform. The most suitable checklist for sample registration depends on the type of sample. For example, there are three checklists for plants: 1) Plant Sample Checklist (https://www.ebi.ac.uk/ena/browser/view/ERC000037), which includes a recommended field named 'source material identifiers', 2) Crop Plant Sample Enhanced Annotation Checklist (https://www.ebi.ac.uk/ena/browser/view/ERC000035), designed to capture enriched annotation of published crop plant samples that lack sufficient reported metadata and are typically associated with systematic transcriptomic realignment-based analyses, and 3) GSC MIxS Plant-Associated Checklist (https://www.ebi.ac.uk/ena/browser/view/ERC000020), a Genomic Standards Consortium extension for reporting environmental measurements and observations related to plant samples is in the process of getting aligned to the BioSamples Plant Minimal Information About Plant Phenotyping Experiment (MIAPPE) Checklist (https://www.ebi.ac.uk/biosamples/schemas/certification/plant-miappe.json) to ensure compatibility and interoperability between omics and phenomic data. Moreover, recent recommendations for a standard Variant Calling Format (VCF) file for plant variant data[2] proposed including persistent identifiers from BioSamples (https://www.ebi.ac.uk/biosamples/) into the metadata fields of the VCF header.

1.2. Genetic Markers

Features such as single-nucleotide polymorphisms (SNPs), insertions/deletions (Indels), and structural variants (SVs) including copy number variations (CNVs) and inversions are key components of genotypic data critical for agricultural breeding and research. In order to facilitate consistent referencing and data integration, public repositories assign standardized identifiers. The Reference SNP ID (rsID, rs# or RefSNP cluster) was introduced in 1998 by dbSNP[3] as a unique identifier for variant loci, clustering variants that share type and genomic position. Initially applied across species, dbSNP now focuses exclusively on human data, while the European Variation Archive (EVA[4]) has assumed responsibility for assigning rsIDs to non-human organisms. rsIDs serve as globally recognized, assembly-independent identifiers, enabling consistent tracking of variants across studies, assemblies, and databases. They support the aggregation of genetic variation observed in different samples or studies, thereby enhancing the resolution and interpretability of datasets. Their stability facilitates cross-referencing in genome browsers, literature, phenotypic databases, and plant and animal breeding platforms. rsIDs have become a staple in the field of human genomics, playing an important role in Genome-Wide Association Studies (GWAS), clinical variant interpretation, and polygenic risk assessments. In agricultural genomics, their use can similarly serve as a foundation for the identification and association between genetic variants and specific traits. This facilitates reproducibility, fosters collaboration, and enables the reuse of existing data. Importantly, rsIDs are routinely remapped to updated genome assemblies or to related varieties within a species, preserving their identity across coordinate systems. In the context of pan-genomes, rsIDs provide a unifying framework for aligning and comparing variants across multiple accessions, enabling the construction of more comprehensive, population-scale variant catalogs[5–7]. For the past two decades, human geneticists have used rsIDs as the prevailing standard reference for SNPs and Indels. In medical genetics, rsIDs are crucial for identifying and referencing variants in genes causing Mendelian disorders[8–12]. Clinical laboratories also use rsIDs to annotate and track variants within their internal databases and to contribute to public databases like dbSNP. Variants identified as "pathogenic" according to the guidelines[13] developed by the American College of Medical Genetics and Genomics (ACMG) and the Association for Molecular Pathology (AMP), have their corresponding dbSNP rsID linked to that classification in ClinVar[10] and eventually in the Online Mendelian Inheritance in Man (OMIM[14]), allowing for easy retrieval and reference in future analyses. Text mining approaches such as LitVar[15] allow to compute and extract relationships between rsIDs and associated entities like diseases and chemicals or drugs. In animal genomics, rsIDs are widely adopted to standardize genetic marker identification and enable interoperability across databases such as OMIA[16], AnimalQTLdb[17], CorrDB[17], and BovineMine[18]. These resources use rsIDs to integrate QTL, GWAS, and variant effect data, facilitating meta-analyses and cross-species comparisons. rsIDs also support computational tools like Ensembl's Variant Effect Predictor (VEP)[19] and AlphaMissense[20] for predicting variant impact, and they simplify literature mining by linking synonyms and assembly versions. Standard file formats (VCF[2], HapMap[21] and PLINK[22]) combined with harmonized metadata ensure robust data sharing and FAIR practices in agricultural genomics.

2. Submitting variants to the European Variation Archive for rsID generation

As the primary repository for rsIDs in non-human organisms, the EVA is the main resource agricultural researchers and bioinformaticians should consult to find rsIDs for their species of interest or to submit new SNP datasets for rsID assignment (Figure S1). To facilitate standardized adoption of rsIDs in agricultural research, we outline the submission and implementation of rsIDs based on established protocols from the EVA (https://www.ebi.ac.uk/eva/?Submit%20Data) and supporting documentation from the ELIXIR FAIR Cookbook (https://w3id.org/faircookbook/FCB061), we are providing a FAIR Implementation Guide (see Supplementary Information annex).

Since variation data are describing modifications from the reference, a key requirement for submission is that the reference genome used in the VCF file must be fully available and deposited in one of the International Nucleotide Sequence Database Collaboration (INSDC) repositories (Figure S1). To support agricultural data producers in making genetic variation datasets FAIR, we have developed a comprehensive FAIR Implementation Guide, detailed below.

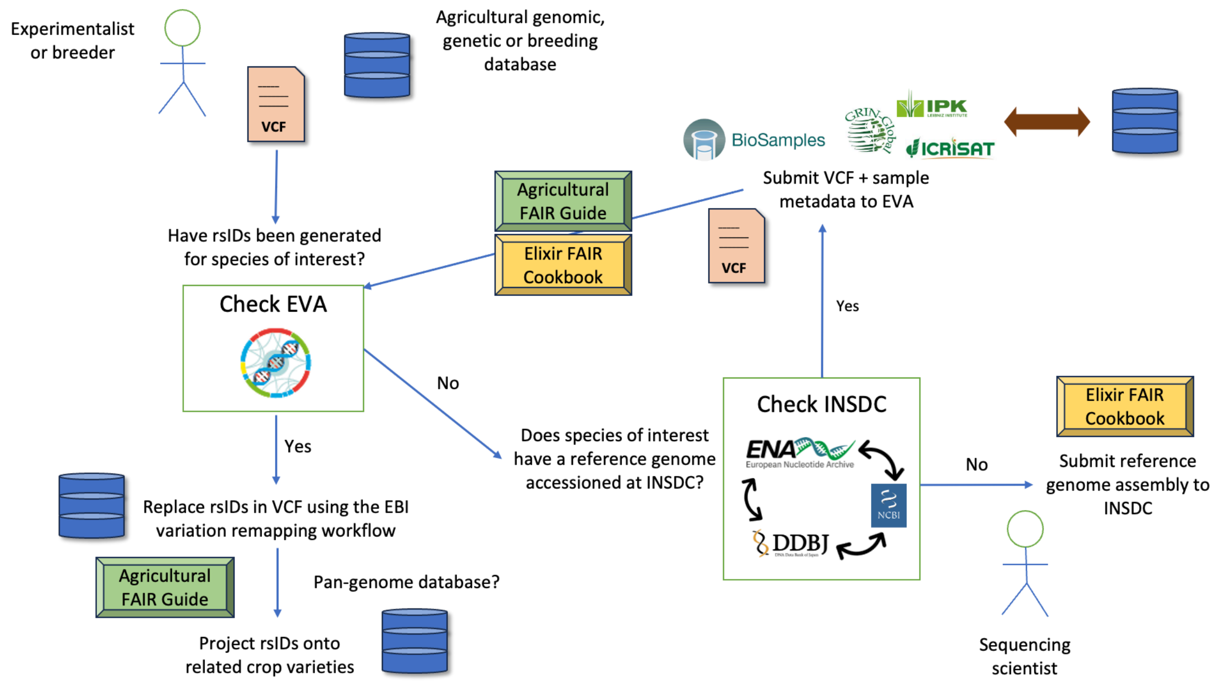

Figure S1. Overview of the rsID adoption and integration process including data submission to the EVA for de novo generation of rsIDs.

The first requirement for a VCF file to be submitted to the EVA is the accession of the reference genome sequence in one of the INSDC archives, which include the ENA, the National Center for Biotechnology Information (NCBI), and the DNA Data Bank of Japan (DDBJ). The submission process starts by following the directions for formatting and validating VCF files from the EVA submission portal (https://www.ebi.ac.uk/eva/?Submit%20Data). Many programs that emit VCF files do not totally conform to the VCF standard. To help in the validation process, the EBI has made available a GitHub repository for a command line tool (CLI, https://github.com/EBIvariation/eva-sub-cli). Detailed setup instructions and usage documentation, including VCF formatting requirements and metadata template descriptions, are available in the repository's documentation. Along with a valid VCF file, the submission process requires filling a metadata template to collect information that will allow EVA to create BioProject, Analysis and Samples entries for the study.

By streamlining and standardizing variant submission processes, EVA plays a critical role in enabling FAIR-compliant genetic variant data for agricultural species. Adoption of these practices ensures that genetic variants are uniquely identified, consistently tracked across genome assemblies, and interoperable across research platforms, laying the groundwork for robust, scalable genotype–phenotype association agricultural studies.

3. Agricultural resources with genetic variation data that have adopted rsIDs or have implementation plans to adopt rsIDs

3.1. SorghumBase and Gramene pan-genome sites

Gramene[23] (https://www.gramene.org) offers comparative genomics and gene annotation resources built upon the Ensembl[24], Reactome[25], EBI Atlas[26] and BAR[27] platforms. The main site, Gramene Plants[23], is kept in sync with Ensembl Plants[28] on a yearly basis, but recently the focus has shifted to crop pan-genome sites for sorghum, maize, rice and grapevine. Each site hosts multiple assembled genomes for each crop, as well as a common set of outgroup "anchor" genomes to support comparative genomic analyses and functional inference. SorghumBase and the Gramene pan-genome sites have been engaged in FAIRification of the genetic variation data for the sorghum, maize, rice, and grapevine research communities. The platform has adopted rsIDs provided by EVA, utilized the variant remapping pipeline to label variants on closely related genomes, worked to standardize germplasm nomenclature, and biocurated phenotypes analyzed in GWAS and QTL studies to standard trait identifiers. These efforts have sparked development of new views within the sites to improve access to genetic resources associated with putative loss-of-function (pLoF) variation (Figure S2).

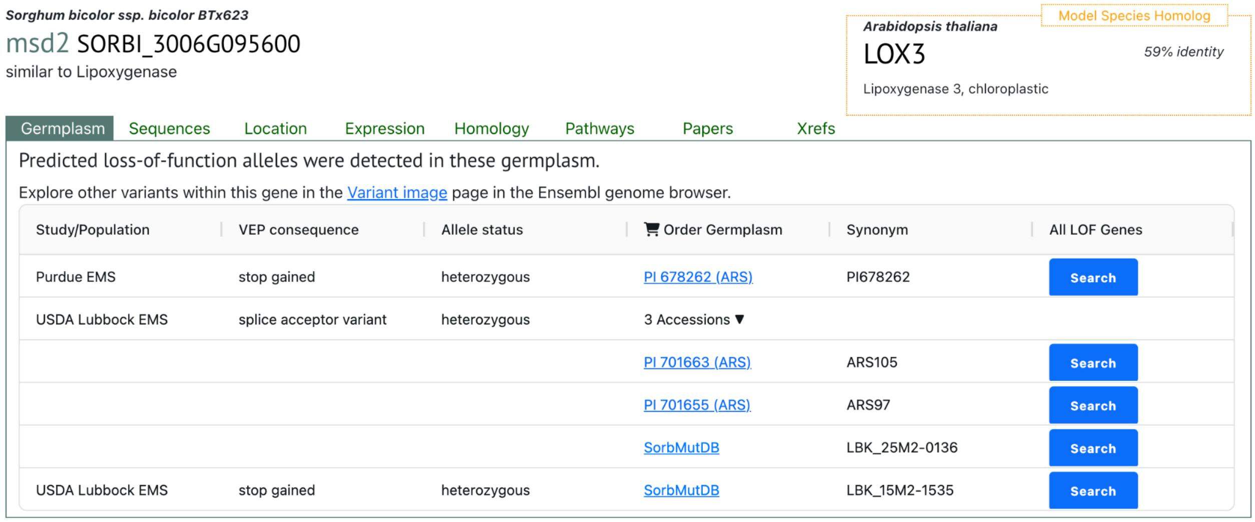

Figure S2. The Germplasm tab in the SorghumBase gene search interface, showing accessions predicted by the Ensembl VEP to harbor pLoF alleles within the target gene. This table is grouped by consequence type and allele heterozygosity and includes links to stock centers, the Ensembl variant image view, and a button to search for other genes predicted to be impacted by the genotype of a specific natural accession or mutant line.

3.2. MaizeGDB: Building a FAIR-compliant resource for genetic variation in maize

MaizeGDB[29] (https://maizegdb.org) provides regular updates of its genetic variation resources by harmonizing diverse datasets to the reference B73 RefGen_v5 coordinate system and presenting them within a unified visualization and analysis framework. MaizeGDB currently hosts three major variant datasets. The first comprises a high-coverage set (~230 million loci) and a high-confidence subset (~75 million loci), derived from remapping publicly available whole-genome sequences representing nearly 1,500 accessions from inbred lines, landraces, and teosintes[30]. The second dataset includes ~36 million SNPs identified from comparative analysis of B73 and the 26 founder inbred lines of the Nested Association Mapping (NAM) panel[31]. The third dataset integrates resequencing data from 1,276 previously published maize accessions and 239 newly sequenced lines, yielding a set of ~46 million high-confidence variants[32]. This panel spans crop wild relatives, landraces, and tropical and temperate lines representing multiple breeding eras. All datasets are accessible through SNPversity[30], an interactive web-based tool that enables variant filtering, visualization, phylogenetic tree construction, and bulk downloads in VCF and HDF5 formats. To support variant interpretation, MaizeGDB has implemented pipelines to annotate the variant effects from these datasets using SnpEff[33], and to predict functional impacts (including likely deleterious variants) using the DNA language model PlantCaduceus[34]. Protein-level consequences are assessed through the ESM protein language model, enabling integrated functional and structural impact scoring. The MaizeGDB genome browser also hosts remapped variants from dbSNP[3] and EVA[4] release 3 (~78 million loci with rsIDs), genome-wide association (GWAS) signals for 321 traits from 157 studies in GWAS Atlas[35], a collection of 41 GWAS-associated traits, trait-associated loci shaped by environmental conditions[36] or breeding history[37], and selective sweep intervals across 28,000 genomic regions[36].

3.3. TreeGenes: Expanding FAIR forestry genomics with CartograPlant

For over 25 years, TreeGenes[38] (https://treegenesdb.org) has supported diverse research needs within the forest tree community by integrating genetic resources with curation and analytical tools. TreeGenes hosts a range of data, from whole genomes and transcripts to genetic markers, phenotypes, proteins, genetic maps, and more. Key data sources include primary repositories like NCBI and Phytozome, alongside direct user submissions. The database is built on Tripal[39,40], an open-source tool that facilitates the creation, sharing, and reuse of genomic data management functionality across repositories. TreeGenes offers a variety of tools for genome visualization (JBrowse), sequence similarity searches (TSeq), data submission (TPPS[39,40]), as well as landscape and population analysis (CartograPlant[41]).

The Tripal Plant PopGen Submit (TPPS[39,40]; https://treegenesdb.org/tpps) pipeline is a user-friendly tool that accepts genotypic, phenotypic, and environmental data, generating a permanent DOI upon submission[39,40]. Built on FAIR principles, TPPS aims to standardize and connect data across datasets through key practices such as the assignment of Digital Object Identifiers (DOIs) to submissions and enforcing the use of standardized metadata, ontologies, and controlled vocabularies. Through these practices TPPS ensures interoperability across

plant genomics and phenotyping datasets and studies. CartograPlant[41] (https://cartograplant.org) is a map-based application that leverages georeferenced data from TPPS and other datasets, providing users with the ability to filter, select, visualize and analyze data on high-performance computational resources. CartograPlant integrates environmental layers and species range maps with data from sources such as TPPS, BIEN database (https://bien.nceas.ucsb.edu/bien), and TreeSnap[42] (https://treesnap.org), a mobile app for citizen science phenotyping. The interface guides users through workflows such as GWAS, which have been adapted specifically for forest tree research.

3.4. GrainGenes and the Triticeae Toolbox (T3)

Genotyping of small grains, which include wheat, barley, rye, and oat, is mainly carried out through standard SNP arrays approved by community-centered consortiums including the 90K array for wheat[43], the 50K array for barley[44], and the 6K array for oats[45]. Other assays actively used for genotyping include amplicon-based genotyping assays and GBS that are also actively used for genotyping assays. The community standard reference genomes for wheat, oat, and barley are Chinese Spring v2.1, OT3098, and Morex V3, respectively. The genotyping data is indexed and stored in centralized databases for small grains (https://graingenes.org/GG3). Large efforts are recently directed towards development of pan-genomic resources for wheat[46,47], barley[48,49] and oat[50].

Two USDA-funded centralized databases for small grains (including wheat, barley, rye, and oat) are GrainGenes[51] (https://graingenes.org) for genetic and genomic data, and the Triticeae Toolbox[52] (T3, https://triticeaetoolbox.org) for genotypic and phenotypic data, follow community guidelines for the FAIR management and visualization of small grains datasets. International collaborations were independently established to create community data standards for wheat[53] and oat[54]. Such standards can be easily adapted to barley and rye given the similarities in the evolutionary history of their chromosomes with wheat. rsIDs have not been widely adopted for small grains.

3.5. From EVA to Ensembl

Ensembl[23] is an open platform that integrates publicly available genomics data across the tree of life, with a focus on eukaryotic species relevant to human health, agriculture, and biodiversity. Agricultural species are distributed across Ensembl's component sites, for example farmed animal genomes are hosted at the main site (ensembl.org), crop genomes at Ensembl Plants (plants.ensembl.org), and insect genomes at Ensembl Metazoa (metazoa.ensembl.org). These sites provide genome assemblies and annotations, including gene models, regulatory features, and genetic variants, together with integrated search tools, protein structures, population-level data, and comparative genomics views. Ensembl data can be accessed via the web interface, large-scale querying through BioMart, FTP downloads, and programmatic access via APIs. Ensembl's infrastructure and data also underpin external sites such as Gramene

(Supplementary Information section 3.1) and BovineMine (Supplementary Information section 3.6), and cross-references are provided to key resources such as EVA, AlphaFoldDB, and UniProt.

From 2026, all species will be consolidated into a single site, currently available in Beta (beta.ensembl.org). The new site already hosts genetic variation data for 55 species, 27 of which are agriculturally relevant (12 crops, 10 livestock, and 5 aquaculture species). Variation data is imported directly from EVA, and genetic variants are displayed, with identifiers, in the Genome Browser, enabling exploration of their genomic context (Figure S3). Users can also investigate variants in greater detail through the Entity Viewer. Transcript consequences generated by Ensembl's VEP, are available in both views, supporting the discovery of variants with potential functional impacts.

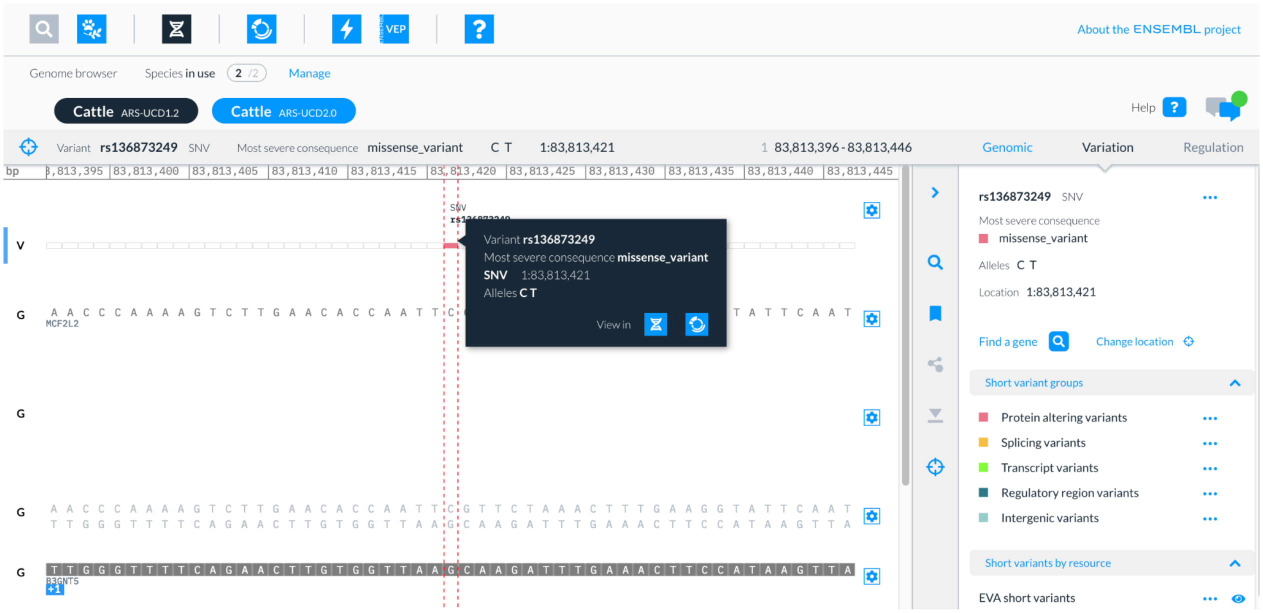

Figure S3. Screenshot showing variation in the genome browser of the new Ensembl site (beta.ensembl.org). The variant's rsID and consequences predicted by Ensembl VEP can be seen in the pop up and side drawer.

3.6. BovineMine: Facilitating the Reuse of Variation Data for Meta-Analysis in Cattle

BovineMine (https://bovinemine.rnet.missouri.edu), the data mining warehouse of the Bovine Genome Database[18], integrates genome annotation data, including genomic variants, from a variety of sources to enable users to retrieve customized datasets and to perform

meta-analyses with their own data. Variation data sources include single-nucleotide variants and variant effect predictions from Ensembl[24], and QTL and GWAS data from AnimalQTLdb[17]. The QTL/GWAS data includes Vertebrate Trait Ontology[55] terms assigned by AnimalQTLdb curators. Both the Ensembl variant data and the AnimalQTLdb QTL/GWAS data include features that were mapped from previous bovine genome assemblies to the most recent bovine genome assembly by different bioinformatics groups, so the data is harmonized across sources by removing references to variants in the QTL/GWAS data that are not concordant with their genome locations in the Ensembl data. The primary Identifiers for the Ensembl variation data are rsIDs, while identifiers from bovine SNP arrays are included as aliases, so variants can be queried with the alternative identifiers.

BovineMine, which was developed based on the InterMine data warehousing platform[56], provides several search tools. The QueryBuilder allows the construction of custom queries, while pre-constructed query templates provide simple menus for users to perform queries based on a single identifier, or term or a list of identifiers (Figure S4). The output is provided as a table that can be sorted, filtered and exported. Data columns within the output table can be saved as lists to use as input in subsequent queries. An additional search tool is the Genomic Regions search which allows users to upload chromosome locations to retrieve overlapping features, including SNP and QTL. By integrating the variation data with information about genes from a multitude of sources, users can explore gene functions and tissue-specific expression in the context of variation data.

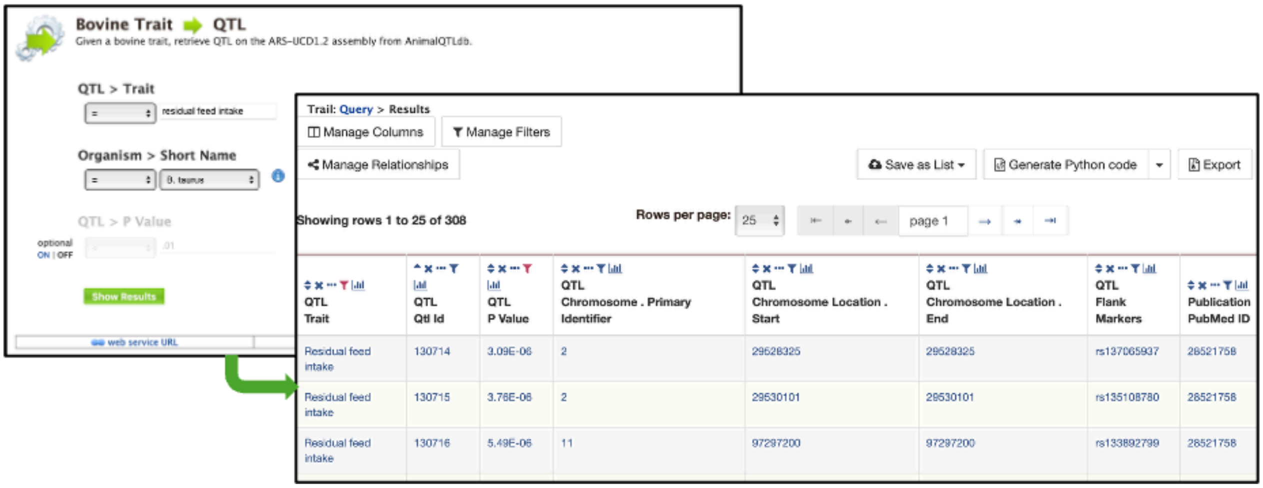

Figure S4. A BovineMine query template for retrieving QTL for a user-specified trait, and the output for the search term “residual feed intake”. rsIDs are used to designate QTL flanking markers, and PubMed identifiers are used to link to the relevant publication with the QTL/GWAS hit, facilitating interoperability across bioinformatics resources.

4. Other Supplementary Methods

Identification of challenges in agricultural standards for genetic variation via surveys, meetings, workshops, panels and breakout room discussions

The SVG WG has held monthly meetings since November 2021 with a steady participation of 5-8 agricultural bioinformatics resources and a total membership of approximately 35. Originally focused on standardizing variant representation and associated metadata, the group expanded its scope in 2024 to include phenotypic trait data following a merger with the Public Genetic Resources Working Group (PGR WG). This expansion reflects a broader recognition that integrated variant and trait data is central to enabling predictive breeding and functional discovery.

In addition, information about genetic variation data being hosted by AgBioData community member resources, as well as validation and quality control practices for interoperability and data reusability was collected through anonymous surveys and workshop discussions sponsored by the AgBioData Consortium. The surveys gathered responses from diverse stakeholders from the AgBioDatra community in order to establish how standards were used in these different resources. The survey comprised 15 questions covering variation dataset characteristics (species, reference assemblies, sequencing technologies, data types), data management practices (storage formats, sharing methods, quality control procedures), metadata linkage (reference genomes, BioSamples, germplasm centers), and workflows for handling reference genome updates and stable variant identifiers. Participants were also asked about their interest in joining the Standards for Genetic Variation (SGV) working group. Responses were collected automatically through the online form. Highlights included that the main datatype among respondents were SNPs; other resources stored SVs, CNVs, and Short-Tandem Repeats (STRs). Although all survey respondents of the 2022 survey performed format validation, less than 60% checked for accuracy between reference allele against the reference genome, genotype count versus ploidy or removing false positives, and less than 30% of respondents claimed to use stable variant identifiers for their variation data. In a 2024 survey, 89% of the respondents were interested in collaboration opportunities with 68% willing to provide genetic markers linked to traits and/or FAIR interoperability information.

These results were presented in the annual AgBioData workshop during which the attending community gave additional input through breakout group structured discussions. A summary of the main challenges identified is presented in Table S1 (see supplementary xlsx file). These exercises highlighted two main challenges: 1) the need of enforcing standard biosample identifiers, and 2) the need to adopt standard rsIDs.

Over a period of three months, United States Department of Agriculture (USDA)-affiliated scientists from the Germplasm Resources Information Network (GRIN) and the National Clonal Germplasm Repository (NCGR) held regular biocuration meetings with data producers and agricultural community database representatives. During such meetings, the recommendations for the formatting of VCF files to make plant genotyping data FAIR[57], as well as relevant plant and animal-associated checklists developed by the EBI, minimum information standard guidelines for reporting biological data, and representative genetic variation data sets for sorghum and strawberry in the Gramene and SorghumBase databases were reviewed. As a

result, additional recommendations to enforce capturing biosample identifiers from a major germplasm repository were made
A systematic review of the scientific literature and of presentations at prominent agricultural genomics meetings such as the Plant and Animal Genomes conference facilitated the identification of genetic variation datasets at various degrees of maturity, to which we herein referred to as the Data Journey, selected to model rsID adoption and projection to pangenomes (Figure 2).

Other products developed through the Data Journey include template emails for supporting submission of a genome assembly to the INSDC, and to expedite generation of rsIDs for commercial genotyping arrays.

Supplementary Information References

1. Courtot, M., Gupta, D., Liyanage, I., Xu, F. & Burdett, T. BioSamples database: FAIRer samples metadata to accelerate research data management. *Nucleic Acids Res.* **50**, D1500–D1507. https://doi.org/10.1093/nar/gkab1046 (2022).
2. Beier, S. *et al.* Recommendations for the formatting of Variant Call Format (VCF) files to make plant genotyping data FAIR. *F1000Res.* **11**, 231. https://doi.org/10.12688/f1000research.109080.1 (2022).
3. Sherry, S. T. *et al.* dbSNP: the NCBI database of genetic variation. *Nucleic Acids Res.* **29**, 308–311. https://doi.org/10.1093/nar/29.1.308 (2001).
4. Cezard, T. *et al.* The European Variation Archive: a FAIR resource of genomic variation for all species. *Nucleic Acids Res.* **50**, D1216–D1220. https://doi.org/10.1093/nar/gkab960 (2022).
5. Wei, S. *et al.* GrameneOryza: a comprehensive resource for *Oryza* genomes, genetic variation, and functional data. *Database* **2025**, baaf021. https://doi.org/10.1093/database/baaf021 (2025).
6. Liao, W.-W. *et al.* A draft human pangenome reference. *Nature* **617**, 312–324. https://doi.org/10.1038/s41586-023-05896-x (2023).
7. Phan, L. *et al.* The evolution of dbSNP: 25 years of impact in genomic research. *Nucleic*

Acids Res. **53**, D925–D931. https://doi.org/10.1093/nar/gkae977 (2025).

8. Amberger, J. S., Bocchini, C. A., Schiettecatte, F., Scott, A. F. & Hamosh, A. OMIM.org: Online Mendelian Inheritance in Man (OMIM®), an online catalog of human genes and genetic disorders. *Nucleic Acids Res.* **43**, D789–D798. https://doi.org/10.1093/nar/gku1205 (2015).
9. Rehm, H. L. *et al.* ClinGen — the clinical genome resource. *N. Engl. J. Med.* **372**, 2235–2242. https://doi.org/10.1056/NEJMsr1406261 (2015).
10. Landrum, M. J. *et al.* ClinVar: public archive of relationships among sequence variation and human phenotype. *Nucleic Acids Res.* **42**, D980–D985. https://doi.org/10.1093/nar/gkt1113 (2014).
11. Shypitsyna, M. K., Thornton, G. R. & Postlethwaite, L. MendelVar: gene prioritisation at GWAS loci using phenotypic enrichment of Mendelian disease genes. *Bioinformatics* **37**, 1–8. https://doi.org/10.1093/bioinformatics/btaa1096 (2021).
12. Sollis, E. *et al.* The NHGRI-EBI GWAS Catalog: knowledgebase and deposition resource. *Nucleic Acids Res.* **51**, D977–D985. https://doi.org/10.1093/nar/gkac1010 (2023).
13. Richards, S. *et al.* Standards and guidelines for the interpretation of sequence variants: a joint consensus recommendation of the American College of Medical Genetics and Genomics and the Association for Molecular Pathology. *Genet. Med.* **17**, 405–424. https://doi.org/10.1038/gim.2015.30 (2015).
14. Amberger, J. S., Bocchini, C. A., Scott, A. F. & Hamosh, A. OMIM.org: leveraging knowledge across phenotype–gene relationships. *Nucleic Acids Res.* **47**, D1038–D1043. https://doi.org/10.1093/nar/gky1151 (2019).
15. Allot, A. *et al.* LitVar: a semantic search engine for linking genomic variant data in PubMed and PMC. *Nucleic Acids Res.* **46**, W530–W536. https://doi.org/10.1093/nar/gky355 (2018).

16. Nicholas, F. & Tammen, I. Online Mendelian Inheritance in Animals (OMIA). University of Sydney. https://doi.org/10.25910/2AMR-PV70 (1995).
17. Hu, Z.-L., Park, C. A. & Reecy, J. M. Bringing the Animal QTLdb and CorrDB into the future: meeting new challenges and providing updated services. *Nucleic Acids Res.* **50**, D956–D961. https://doi.org/10.1093/nar/gkab1116 (2022).
18. Shamimuzzaman, M. *et al.* Bovine Genome Database: new annotation tools for a new reference genome. *Nucleic Acids Res.* **48**, D676–D681. https://doi.org/10.1093/nar/gkz944 (2020).
19. McLaren, W. *et al.* The Ensembl Variant Effect Predictor. *Genome Biol.* **17**, 122. https://doi.org/10.1186/s13059-016-0974-4 (2016).
20. Cheng, J. *et al.* Accurate proteome-wide missense variant effect prediction with AlphaMissense. *Science* **381**, eadg7492. https://doi.org/10.1126/science.adg7492 (2023).
21. International HapMap Consortium. A haplotype map of the human genome. *Nature* **437**, 1299–1320. https://doi.org/10.1038/nature04226 (2005).
22. Purcell, S. *et al.* PLINK: a tool set for whole-genome association and population-based linkage analyses. *Am. J. Hum. Genet.* **81**, 559–575. https://doi.org/10.1086/519795 (2007).
23. Olson, A. *et al.* Gramene 2025: expanded comparative genomics and pathway resources, integrated search, and pan-genome portals for crop research. *Nucleic Acids Res.* **54**, D1720–D1732. https://doi.org/10.1093/nar/gkaf1260 (2025).
24. Dyer, S. C. *et al.* Ensembl 2025. *Nucleic Acids Res.* **53**, D948–D957. https://doi.org/10.1093/nar/gkae1071 (2025).
25. Naithani, S. *et al.* Plant Reactome: a knowledgebase and resource for comparative pathway analysis. *Nucleic Acids Res.* **48**, D1093–D1103. https://doi.org/10.1093/nar/gkz996 (2020).

26. Moreno, P. *et al.* Expression Atlas update: gene and protein expression in multiple species. *Nucleic Acids Res.* **50**, D129–D140. https://doi.org/10.1093/nar/gkab1030 (2022).
27. Sullivan, A. *et al.* 20 years of the Bio-Analytic Resource for Plant Biology. *Nucleic Acids Res.* **53**, D1576–D1586. https://doi.org/10.1093/nar/gkae920 (2025).
28. Yates, A. D. *et al.* Ensembl Genomes 2022: an expanding genome resource for non-vertebrates. *Nucleic Acids Res.* **50**, D996–D1003. https://doi.org/10.1093/nar/gkab1007 (2022).
29. Woodhouse, M. R. *et al.* A pan-genomic approach to genome databases using maize as a model system. *BMC Plant Biol.* **21**, 385. https://doi.org/10.1186/s12870-021-03173-5 (2021).
30. Andorf, C. M., Ross-Ibarra, J., Seetharam, A. S., Hufford, M. B. & Woodhouse, M. R. A unified VCF dataset from nearly 1,500 diverse maize accessions and resources to explore the genomic landscape of maize. *G3 (Bethesda)* **15**, jkae281. https://doi.org/10.1093/g3journal/jkae281 (2025).
31. Hufford, M. B. *et al.* De novo assembly, annotation, and comparative analysis of 26 diverse maize genomes. *Science* **373**, 655–662. https://doi.org/10.1126/science.abg5289 (2021).
32. Grzybowski, M. W. *et al.* A common resequencing-based genetic marker data set for global maize diversity. *Plant J.* **113**, 1109–1121. https://doi.org/10.1111/tpj.16123 (2023).
33. Cingolani, P. *et al.* A program for annotating and predicting the effects of single nucleotide polymorphisms, SnpEff: SNPs in the genome of *Drosophila melanogaster* strain w1118; iso-2; iso-3. *Fly* **6**, 80–92. https://doi.org/10.4161/fly.19695 (2012).
34. Zhai, J. *et al.* Cross-species modeling of plant genomes at single-nucleotide resolution using a pretrained DNA language model. *Proc. Natl Acad. Sci. USA* **122**, e2421738122. https://doi.org/10.1073/pnas.2421738122 (2025).

35. Tian, D. *et al.* GWAS Atlas: a curated resource of genome-wide variant–trait associations in plants and animals. *Nucleic Acids Res.* **48**, D927–D932. https://doi.org/10.1093/nar/gkz828 (2020).
36. Li, C. *et al.* Genomic insights into historical improvement of heterotic groups during modern hybrid maize breeding. *Nat. Plants* **8**, 750–763. https://doi.org/10.1038/s41477-022-01190-2 (2022).
37. Tibbs-Cortes, L. E., Guo, T., Andorf, C. M., Li, X. & Yu, J. Comprehensive identification of genomic and environmental determinants of phenotypic plasticity in maize. *Genome Res.* **34**, 1253–1263. https://doi.org/10.1101/gr.279027.124 (2024).
38. Falk, T. *et al.* Growing and cultivating the forest genomics database, TreeGenes. *Database* **2018**, bay084. https://doi.org/10.1093/database/bay084 (2018).
39. Wegrzyn, J. L. *et al.* Cyberinfrastructure to improve forest health and productivity: the role of tree databases in connecting genomes, phenomes, and the environment. *Front. Plant Sci.* **10**, 813. https://doi.org/10.3389/fpls.2019.00813 (2019).
40. Staton, M. *et al.* Tripal, a community update after 10 years of supporting open source, standards-based genetic, genomic and breeding databases. *Brief. Bioinform.* **22**, bbaa238. https://doi.org/10.1093/bib/bbab238 (2021).
41. Lind, B. *et al.* CartograPlant: bridging genomic, phenotypic, and environmental data to advance plant resilience and eco-evolutionary insight. Preprint at *EcoEvoRxiv*. https://doi.org/10.32942/x2q06d (2025).
42. Crocker, E. *et al.* TreeSnap: a citizen science app connecting tree enthusiasts and forest scientists. *Plants People Planet* **2**, 47–52. https://doi.org/10.1002/ppp3.41 (2020).
43. Wang, S. *et al.* Characterization of polyploid wheat genomic diversity using a high-density 90,000 single nucleotide polymorphism array. *Plant Biotechnol. J.* **12**, 787–796. https://doi.org/10.1111/pbi.12183 (2014).
44. Bayer, M. M. *et al.* Development and evaluation of a barley 50k iSelect SNP array. *Front.*

Plant Sci. **8**, 1792. https://doi.org/10.3389/fpls.2017.01792 (2017).

45. Tinker, N. A. *et al.* A SNP genotyping array for hexaploid oat. *Plant Genome* **7**, plantgenome2014.03.0010. https://doi.org/10.3835/plantgenome2014.03.0010 (2014).
46. Walkowiak, S. *et al.* Multiple wheat genomes reveal global variation in modern breeding. *Nature* **588**, 277–283. https://doi.org/10.1038/s41586-020-2961-x (2020).
47. Jiao, C. *et al.* Pan-genome bridges wheat structural variations with habitat and breeding. *Nature* **637**, 384–393. https://doi.org/10.1038/s41586-024-08277-0 (2025).
48. Jayakodi, M. *et al.* The barley pan-genome reveals the hidden legacy of mutation breeding. *Nature* **588**, 284–289. https://doi.org/10.1038/s41586-020-2947-8 (2020).
49. Jayakodi, M. *et al.* Structural variation in the pangenome of wild and domesticated barley. *Nature* **636**, 654–662. https://doi.org/10.1038/s41586-024-08187-1 (2024).
50. Avni, R. *et al.* A pangenome and pantranscriptome of hexaploid oat. *Nature* **637**, 112–121. https://doi.org/10.1038/s41586-025-09676-7 (2025).
51. Yao, E. *et al.* GrainGenes: genetics, genomes, and pangenomes. *Genetics*. https://doi.org/10.1093/genetics/iyaf270 (2025).
52. Blake, V. C. *et al.* The Triticeae Toolbox: combining phenotype and genotype data to advance small-grains breeding. *Plant Genome* **9**, plantgenome2014.12.0099. https://doi.org/10.3835/plantgenome2014.12.0099 (2016).
53. Boden, S. A. *et al.* Updated guidelines for gene nomenclature in wheat. *Theor. Appl. Genet.* **136**, 72. https://doi.org/10.1007/s00122-023-04253-w (2023).
54. Jellen, E. N. *et al.* A uniform gene and chromosome nomenclature system for oat (*Avena* spp.). *Crop Pasture Sci.* **75**, CP23247. https://doi.org/10.1071/CP23247 (2024).
55. Park, C. A. *et al.* The Vertebrate Trait Ontology: a controlled vocabulary for the annotation of trait data across species. *J. Biomed. Semant.* **4**, 13. https://doi.org/10.1186/2041-1480-4-13 (2013).
56. Smith, R. N. *et al.* InterMine: a flexible data warehouse system for the integration and

analysis of heterogeneous biological data. *Bioinformatics* **28**, 3163–3165. https://doi.org/10.1093/bioinformatics/bts577 (2012).

57. Wilkinson, M. D. *et al.* The FAIR Guiding Principles for scientific data management and stewardship. *Sci. Data* **3**, 160018. https://doi.org/10.1038/sdata.2016.18 (2016).

Table S1. Summary of challenges and proposed solutions for the FAIRification of genetic variation data in agricultural research

Challenge	Premise, pre-requisites, potential implications	Proposed solutions
Standard marker identifiers needed for agriculturally important SNP datasets	GWAS link agriculturally important traits with SNPs	1) Identify representative genetic variation (GV) data sets for a pilot rsID adoption exercise<br>2) Select examples at different stages of a "data journey" (i.e., Submission, Database adoption, Pan-genome projection, Commercial arrays, and Interoperability)<br>3) Document requirements for submitting selected GV sets to the EVA, identify granular challenges (eg, lack of INSDC-indexed reference genome, limited community resources for this task, etc), and model solutions like identifying key SNP sets to broker submission, writing a user guide to help data producers to submit data to EVA, etc.
Published studies not providing standard identifiers for markers or samples	Requires stakeholders to understand that standard samples and marker identifiers are necessary for data reuse	Requires stakeholders to understand that standard samples and marker identifiers are necessary for data reuse
Reference genome not accessioned in an INSDC database	SNPs must be mapped to an INSDC-accessioned genome for submission to the EVA. Data producers struggle with data submission to repositories like the INSDC for genome sequence assemblies and EVA for genetic variation (associated to accessioned genome assemblies)	1) Contact data producers to encourage assembly submission<br>2) Promote existing educational resources like the protocol for genome assembly submissions to the ENA in the ELIXIR Handbook<br>3) Promote the creation of additional INSDC hubs by geographical region<br>4) Devote funding from agricultural community databases to support (not actually format or fix errors) assembly submissions
Lack of standard marker identifiers in agricultural community databases	rsIDs are widely adopted in human genomics, specifically in GWAS, clinical variant interpretation, and large-scale meta-analyses. However, their adoption in agriculture has lagged behind	1) Model the adoption of reference identifers (rsIDs) by a community resource as proof-of-principle<br>2) Integrate rsIDs with agricultural trait data to promote adoption and develop educational materials<br>3) Propagate rsIDs across varieties of the same agricultural species (eg, sorghum pangenome)
Published studies not providing variant data in a standard file format like VCF	Stakeholders need to understand why machine-readable data is important for data sharing and reuse (e.g., meta-analyses), data integration (e.g., genotypes with trait data), and interoperability across bioinformatic resources, as well asunderstand how adding more SNP data can help research and add more value to the data	Enlist journal editors to make mandatory the use of standard file formats like VCF, PLINK or HapMap and implement automatic validation checks
Data producers struggle formatting data in standard file formats like VCF. Incidentally, some programs do not output correctly the desired format	Stakeholders need to understand why a correct VCF is important. VCF validators exist, but may not be widely known nor used	1) Promote existing educational material and data format conversion and validation tools<br>2) Promote the existence of VCF validators and make them readily available to data producers. Examples: VCFtools validator (https: //vcftoolsgithubio/perl_modulehtml) and EBIvariation/vcf-validator (https: //githubcom/EBIvariation/vcf-validator)<br>3) Community databases with designated funding could support users to validate (not actually format or fix errors)<br>4) Scientific journals and funders could provide incentives to make genetic variation data FAIR
EVA submission and recent VCF recommendations for plant data (Beier et al, 2022) proposed using BioSample identifiers, but those are not necessarily linked to a major germplasm repository	Associated biosamples metadata is required for SNP data submission to the EVA. Major germplasm repositories provide additional germplasm information and are the potential source of raw material for experimental study design and replication	1) Coordinate with EBI BioSamples to require mandatory germplasm IDs and a DOI/URL from a major germplasm center or community DB for all new sample submissions<br>2) Suggest that a sample DOI/URL from a major germplasm center or community DB is a required field for EVA submissions

Table S2. Sorghum Community Marker Panel (SCMP) SNPs with corresponding standard rsIDs

rsID	chromosome #	position	variant	SNP_name	associated trait category	50_Trait; 42_QC	notes (26 EVA_fast_track; 17 not_in_MDP)
rs875437421	2	6045380	-:G>A	snpSB00285	Fertility restoration gene	Trait-associated	
rs162432214	2	6843380	-:A>G	snpSB00286	Fertility restoration gene	Trait-associated	
rs874941941	2	8715821	-:A>G	snpSB00287	Fertility restoration gene	Trait-associated	
rs5980786983	2	59000770	-:A>C	snpSB0040	Post-flowering Drought tolerance	Trait-associated	EVA_fast_track
rs5980786973	2	59821923	-:T>G	snpSB0049	Post-flowering Drought tolerance	Trait-associated	EVA_fast_track
rs5980786986	2	60098184	-:T>G	snpSB0054	Post-flowering Drought tolerance	Trait-associated	EVA_fast_track
rs5980786874	2	61811307	-:T>A	snpSB00072	Post-flowering Drought tolerance; Fertility restoration gene	Trait-associated	EVA_fast_track
rs5980786977	2	67306935	-:A>C	snpSB0091	Post-flowering Drought tolerance	Trait-associated	EVA_fast_track
rs5980786985	2	67710384	-:T>A	snpSB0095	Post-flowering Drought tolerance	Trait-associated	EVA_fast_track
rs5980786976	2	71419274	~~-:A>C~~	snpSB0103	Post-flowering Drought tolerance	Trait-associated	EVA_fast_track
rs5980786918	3	30310883	-:T>G	snpSB00294	Fertility restoration gene	Trait-associated	EVA_fast_track
rs5980786984	4	364279	-:C>T	snpSB00297	Fertility restoration gene	Trait-associated	EVA_fast_track
rs266811913	5	838874	-:C>T	snpSB00106	Shoot fly resistance	Trait-associated	
rs266842621	5	1608322	-:G>A	snpSB00302	Fertility restoration gene	Trait-associated	
rs875142952	5	69794954	-:G>A	snpSB00253	Striga Resistance	Trait-associated	
rs162648388	5	69847924	-:C>T	snpSB00257	Striga Resistance	Trait-associated	
rs266387781	5	69851828	-:C>G	snpSB00255	Striga Resistance	Trait-associated	
rs266488695	5	69852443	-:A>G	snpSB00258	Striga Resistance	Trait-associated	
rs875519833	6	2682627	-:C>A	snpSB00315	Sugarcane Aphid Resistance	Trait-associated	
rs5435303193	6	2892438	-:C>G	snpSB00317	Sugarcane Aphid Resistance	Trait-associated	
rs162520331	8	653850	-:C>T	snpSB00306	Fertility restoration gene	Trait-associated	
rs162448701	8	60934182	-:G>C	snpSB00311	Fertility restoration gene	Trait-associated	
rs5446174524	9	4248350	-:A>G	snpSB00313	Fertility restoration gene	Trait-associated	
rs162481182	9	46616891	-:A>G	snpSB00312	Fertility restoration gene	Trait-associated	
rs162690324	10	58031253	-:C>G	snpSB00158	Shoot fly resistance	Trait-associated	
rs872191269	10	60919657	-:T>C	snpSB00169	Shoot fly resistance	Trait-associated	
rs162572125	1	7738853	-:C>T	snpSB00319	QC Panel	QC	
rs266975286	1	12758577	-:G>T	snpSB00321	QC Panel	QC	
rs162424710	1	25582611	-:A>G	snpSB00323	QC Panel	QC	
rs5420820633	2	47150048	-:A>G	snpSB00330	QC Panel	QC	
rs162611012	3	57493392	-:C>A	snpSB00338	QC Panel	QC	
rs266881600	4	1120014	-:G>C	snpSB00341	QC Panel	QC	
rs266531776	4	45602443	-:C>T	snpSB00343	QC Panel	QC	
rs873953033	4	52519811	-:G>A	snpSB00344	QC Panel	QC	
rs266989303	4	56727047	-:G>C	snpSB00345	QC Panel	QC	
rs162544388	5	8830742	-:T>C	snpSB00346	QC Panel	QC	
rs162613077	5	12453029	-:T>C	snpSB00347	QC Panel	QC	
rs5431145722	5	15917442	-:G>C	snpSB00348	QC Panel	QC	
rs162582149	5	50894812	-:G>A	snpSB00357	QC Panel	QC	

rs5434387785	5	62034047	-:G>A	snpSB00358	QC Panel	QC	
rs162665304	5	62509203	-:G>A	snpSB00359	QC Panel	QC	
rs3380086475	5	66910484	-:A>C	snpSB00360	QC Panel	QC	
rs874478149	5	67987987	-:A>G	snpSB00361	QC Panel	QC	
rs267071114	6	3199668	-:A>G	snpSB00364	QC Panel	QC	
rs266636670	6	3898444	-:T>C	snpSB00365	QC Panel	QC	
rs162420864	6	56193976	-:A>G	snpSB00369	QC Panel	QC	
rs162458192	10	49144697	-:T>C	snpSB00387	QC Panel	QC	
rs266746955	10	53353591	-:G>A	snpSB00389	QC Panel	QC	
rs875246538	1	18115987	-:C>T	snpSB00322	-		
rs873615678	2	7200900	-:G>A	snpSB00329	-		
rs162612394	2	65774198	-:A>G	snpSB00331	-		
rs5980786987	3	26141400	-:A>G	snpSB00336	-		EVA_fast_track
rs3379981054	3	60434436	-:C>T	snpSB00339	-		
rs873121350	3	67917118	-:T>A	snpSB00340	-		
rs795939341	4	26389679	-:T>C	snpSB00342	-		
rs5980786925	8	6429359	-:A>T	snpSB00375	-		EVA_fast_track
rs5446100135	9	5988640	-:G>A	snpSB00377	-		
rs266641355	9	9992707	-:C>A	S9_9992707	-		
rs162455275	9	35139068	-:G>C	snpSB00381	-		
rs5980786899	10	17655615	-:A>G	snpSB00385	-		EVA_fast_track
rs162407221	1	7953720	-:A>G	S1_7953720	-		
rs162407765	3	73926129	-:G>A	S3_73926129	-		
rs162408441	2	3711510	-:T>A	S2_3711510	-		
rs162408786	4	12642773	-:T>C	S4_12642773	-		
rs162409680	2	73494298	-:A>G	S2_73494298	-		
rs162409722	3	6692636	-:T>C	S3_6692636	-		
rs162410181	2	63466945	-:A>C	S2_63466945	-		
rs162410214	3	5643185	-:T>A	S3_5643185	-		
rs162410279	5	6866446	-:C>G	S5_6866446	-		
rs162411295	6	57987100	-:G>C	S6_57987100	-		
rs162411450	1	60657131	-:T>C	S1_60657131	-		
rs162411830	10	9239499	-:C>T	S10_9239499	-		
rs162412640	4	8525989	-:T>C	S4_8525989	-		
rs162413518	2	8707876	-:G>C	S2_8707876	-		
rs162413649	2	3272882	-:C>G	S2_3272882	-		
rs162413961	8	5017626	-:A>G	S8_5017626	-		
rs162415938	5	1584375	-:T>C	S5_1584375	-		
rs162417229	1	21334477	-:C>T	S1_21334477	-		
rs162417804	10	55748370	-:G>C	S10_55748370	-		
rs162417995	6	46493884	-:C>G	S6_46493884	-		
rs162418509	3	58715211	-:G>A	S3_58715211	-		
rs162418735	7	3541178	-:T>A	S7_3541178	-		

rs162420440	3	65324887	-:C>A	S3_65324887	-		
rs162420866	8	57274084	-:G>A	S8_57274084	-		
rs162421892	4	48061038	-:C>T	S4_48061038	-		
rs162422054	10	10169416	-:C>A	S10_10169416	-		
rs162422443	5	7145698	-:C>T	S5_7145698	-		
rs162422462	2	52125078	-:G>T	S2_52125078	-		
rs162423323	5	62754800	-:G>T	S5_62754800	-		
rs162423637	3	2931829	-:G>A	S3_2931829	-		
rs162425358	2	61121980	-:T>C	S2_61121980	-		
rs162425432	1	5771152	-:C>T	S1_5771152	-		
rs162425892	2	72190033	-:T>C	S2_72190033	-		
rs162426488	1	52223324	-:G>C	S1_52223324	-		
rs162427011	1	4583054	-:T>G	S1_4583054	-		
rs162428798	2	1829560	-:G>A	S2_1829560	-		
rs162429233	10	41667577	-:T>G	S10_41667577	-		
rs162430360	5	68538260	-:A>C	S5_68538260	-		
rs162430372	6	58115768	-:A>C	S6_58115768	-		
rs162431426	4	57959532	-:G>T	S4_57959532	-		
rs162431563	7	39076729	-:T>G	S7_39076729	-		
rs162431817	3	4872727	-:G>A	S3_4872727	-		
rs162431838	6	42832536	-:G>C	S6_42832536	-		
rs162431919	5	6991478	-:G>A	S5_6991478	-		
rs162433146	7	8105662	-:A>G	S7_8105662	-		
rs162433349	8	62023661	-:A>C	S8_62023661	-		
rs162433667	1	16745318	-:G>A	S1_16745318	-		
rs162434864	4	54798745	-:A>G	S4_54798745	-		
rs162435454	3	57901123	-:A>G	S3_57901123	-		
rs162435526	2	68273775	-:G>T	S2_68273775	-		
rs162436244	6	42047398	-:C>T	S6_42047398	-		
rs162436293	1	3910367	-:G>A	S1_3910367	-		
rs162437366	5	3631544	-:G>C	S5_3631544	-		
rs162437669	5	64396901	-:G>A	S5_64396901	-		
rs162437835	8	1268567	-:A>G	S8_1268567	-		
rs162438571	4	57914648	-:C>A	S4_57914648	-		
rs162438744	3	64937605	-:T>C	S3_64937605	-		
rs162438783	3	7419358	-:T>G	S3_7419358	-		
rs162439396	3	71798340	-:C>A	S3_71798340	-		
rs162439732	3	71654172	-:C>T	S3_71654172	-		
rs162439765	6	47114408	-:G>C	S6_47114408	-		
rs162440261	8	55725813	-:A>T	S8_55725813	-		
rs162440580	7	65083854	-:T>G	S7_65083854	-		
rs162441018	6	47644622	-:T>G	S6_47644622	-		
rs162441376	9	54245690	-:C>T	S9_54245690	-		

rs162441443	1	63955927	-:C>T	S1_63955927	-		
rs162441586	1	73644644	-:A>G	S1_73644644	-		
rs162441729	6	42256151	-:G>A	S6_42256151	-		
rs162443505	6	55698548	-:A>G	S6_55698548	-		
rs162443553	3	74011150	-:T>C	S3_74011150	-		
rs162443624	6	57074740	-:C>A	S6_57074740	-		
rs162443733	2	76684198	-:A>G	S2_76684198	-		
rs162443759	9	55826895	-:T>G	S9_55826895	-		
rs162443948	3	58433150	-:A>T	S3_58433150	-		
rs162444033	8	60931222	-:C>T	S8_60931222	-		
rs162444328	7	10287100	-:A>G	S7_10287100	-		
rs162444421	9	49337473	-:A>G	S9_49337473	-		
rs162444471	1	62992453	-:A>G	S1_62992453	-		
rs162444920	7	63325621	-:G>A	S7_63325621	-		
rs162444946	7	62481758	-:C>T	S7_62481758	-		
rs162444978	6	51539086	-:G>A	S6_51539086	-		
rs162445204	10	12320475	-:T>C	S10_12320475	-		
rs162445229	4	67359962	-:G>T	S4_67359962	-		
rs162445596	7	15545781	-:G>A	S7_15545781	-		
rs162446900	5	3022709	-:A>G	S5_3022709	-		
rs162447582	9	52937785	-:T>G	S9_52937785	-		
rs162447894	5	65847349	-:C>A	S5_65847349	-		
rs162448373	3	7813955	-:T>G	S3_7813955	-		
rs162448642	9	47254520	-:A>T	S9_47254520	-		
rs162448759	7	62966410	-:T>C	S7_62966410	-		
rs162449240	3	57216109	-:C>G	S3_57216109	-		
rs162449500	5	71726595	-:A>G	S5_71726595	-		
rs162449721	1	25075987	-:C>T	S1_25075987	-		
rs162449917	6	47420268	-:G>A	S6_47420268	-		
rs162450431	7	9425922	-:T>G	S7_9425922	-		
rs162450780	4	61295445	-:T>C	S4_61295445	-		
rs162450814	1	11169624	-:A>T	S1_11169624	-		
rs162451227	2	76427402	-:G>C	S2_76427402	-		
rs162452841	5	11781274	-:T>A	S5_11781274	-		
rs162453899	6	49712021	-:C>T	S6_49712021	-		
rs162453901	8	5242383	-:A>G	S8_5242383	-		
rs162454410	10	9650899	-:C>A	S10_9650899	-		
rs162454849	6	6401507	-:A>G	S6_6401507	-		
rs162454888	6	47539333	-:G>T	S6_47539333	-		
rs162455136	6	5959735	-:G>T	S6_5959735	-		
rs162455173	6	44076824	-:A>T	S6_44076824	-		
rs162455414	1	11322213	-:T>A	S1_11322213	-		
rs162455746	2	63368936	-:A>C	S2_63368936	-		

rs162455775	2	59198266	-:G>C	S2_59198266	-		
rs162455904	10	6015658	-:A>G	S10_6015658	-		
rs162456377	6	5765055	-:A>C	S6_5765055	-		
rs162456642	1	12223183	-:A>C	S1_12223183	-		
rs162457341	1	80207929	-:T>C	S1_80207929	-		
rs162457418	1	9366502	-:T>C	S1_9366502	-		
rs162458255	6	1911065	-:G>C	S6_1911065	-		
rs162458700	4	42504520	-:T>G	S4_42504520	-		
rs162459744	2	67844602	-:A>C	S2_67844602	-		
rs162461560	1	25497994	-:A>G	S1_25497994	-		
rs162462101	6	52051667	-:G>C	S6_52051667	-		
rs162462933	1	58396849	-:T>A	S1_58396849	-		
rs162463312	3	63390012	-:C>T	S3_63390012	-		
rs162464617	3	9795565	-:C>T	S3_9795565	-		
rs162464881	6	53990048	-:C>T	S6_53990048	-		
rs162465869	3	59206666	-:G>A	S3_59206666	-		
rs162466158	1	7327681	-:T>A	S1_7327681	-		
rs162466773	3	54098203	-:G>C	S3_54098203	-		
rs162467063	1	13156348	-:G>A	S1_13156348	-		
rs162467643	4	12205807	-:T>C	S4_12205807	-		
rs162468382	5	56707928	-:G>C	S5_56707928	-		
rs162468401	3	61294292	-:A>C	S3_61294292	-		
rs162469398	6	47677252	-:A>G	S6_47677252	-		
rs162469523	10	6285658	-:A>T	S10_6285658	-		
rs162469790	6	58761978	-:C>G	S6_58761978	-		
rs162469915	4	51515678	-:C>A	S4_51515678	-		
rs162470920	7	6343905	-:A>T	S7_6343905	-		
rs162472864	1	21487572	-:T>C	S1_21487572	-		
rs162472933	6	47315279	-:A>G	S6_47315279	-		
rs162475050	7	63869230	-:C>G	S7_63869230	-		
rs162475182	5	60450850	-:C>G	S5_60450850	-		
rs162475323	3	69966714	-:T>C	S3_69966714	-		
rs162475456	3	68088263	-:C>T	S3_68088263	-		
rs162475583	8	58972170	-:C>T	S8_58972170	-		
rs162475772	1	14831774	-:A>G	S1_14831774	-		
rs162475784	8	2604994	-:C>G	S8_2604994	-		
rs162475951	4	59190120	-:C>T	S4_59190120	-		
rs162476450	9	6375482	-:C>G	S9_6375482	-		
rs162477119	4	47809811	-:A>G	S4_47809811	-		
rs162478180	7	64737987	-:A>G	S7_64737987	-		
rs162478213	6	31581245	-:T>C	S6_31581245	-		
rs162478314	8	62482707	-:T>C	S8_62482707	-		
rs162478847	8	57039500	-:A>G	S8_57039500	-		

rs162479017	1	76494959	-:C>G	S1_76494959	-			
rs162479782	10	54952555	-:C>T	S10_54952555	-			
rs162480370	6	47674369	-:G>A	S6_47674369	-			
rs162480442	9	55750462	-:C>T	S9_55750462	-			
rs162481245	3	16372555	-:C>T	S3_16372555	-			
rs162481252	1	61354706	-:C>G	S1_61354706	-			
rs162481994	8	14252601	-:C>T	S8_14252601	-			
rs162482012	5	58856853	-:C>T	S5_58856853	-			
rs162482168	6	48582278	-:G>A	S6_48582278	-			
rs162482343	10	55364827	-:A>G	S10_55364827	-			
rs162482913	1	19245447	-:C>G	S1_19245447	-			
rs162483845	2	63140283	-:C>G	S2_63140283	-			
rs162483869	10	47071511	-:A>G	S10_47071511	-			
rs162483914	2	63192891	-:G>C	S2_63192891	-			
rs162484812	4	52935808	-:G>A	S4_52935808	-			
rs162484965	10	53018027	-:G>A	S10_53018027	-			
rs162485405	4	63788767	-:G>A	S4_63788767	-			
rs162485439	2	62095767	-:T>G	S2_62095767	-			
rs162485473	9	52554672	-:G>A	S9_52554672	-			
rs162486429	5	44815496	-:A>G	S5_44815496	-			
rs162486863	6	60398529	-:T>G	S6_60398529	-			
rs162487163	6	59456620	-:A>T	S6_59456620	-			
rs162487227	3	73239278	-:G>C	S3_73239278	-			
rs162487288	2	62216434	-:G>A	S2_62216434	-			
rs162487599	2	12203631	-:A>T	S2_12203631	-			
rs162488593	2	11656286	-:C>T	S2_11656286	-			
rs162488645	2	8715502	-:A>G	S2_8715502	-			
rs162488652	7	60758224	-:C>G	S7_60758224	-			
rs162488720	10	56744957	-:C>G	S10_56744957	-			
rs162489963	3	62421152	-:A>G	S3_62421152	-			
rs162490580	3	7121906	-:T>C	S3_7121906	-			
rs162490912	6	3223724	-:T>C	S6_3223724	-			
rs162491269	5	71606153	-:A>T	S5_71606153	-			
rs162491399	8	4142831	-:C>A	S8_4142831	-			
rs162491678	1	17790489	-:A>C	S1_17790489	-			
rs162491703	8	3193897	-:A>G	S8_3193897	-			
rs162491848	1	68245228	-:G>T	S1_68245228	-			
rs162492091	8	55589108	-:A>C	S8_55589108	-			
rs162492660	3	19140853	-:T>G	S3_19140853	-			
rs162492869	7	64424595	-:A>G	S7_64424595	-			
rs162494124	7	63713076	-:C>T	S7_63713076	-			
rs162494491	1	78944590	-:G>A	S1_78944590	-			
rs162494734	7	164687	-:C>T	S7_164687	-			

rs162495217	1	10687530	-:C>G	S1_10687530	-			
rs162495283	5	69923151	-:C>G	S5_69923151	-			
rs162495372	8	10795485	-:G>T	S8_10795485	-			
rs162495966	1	25467252	-:G>A	S1_25467252	-			
rs162496744	4	46929793	-:G>T	S4_46929793	-			
rs162497160	5	9430686	-:A>T	S5_9430686	-			
rs162497515	1	55109970	-:T>G	S1_55109970	-			
rs162497888	3	2073442	-:A>T	S3_2073442	-			
rs162498176	6	46071845	-:C>T	S6_46071845	-			
rs162498426	1	69919377	-:T>C	S1_69919377	-			
rs162498927	3	70886462	-:A>G	S3_70886462	-			
rs162498929	4	62878310	-:G>A	S4_62878310	-			
rs162501116	3	60937459	-:A>G	S3_60937459	-			
rs162501471	2	63061898	-:A>C	S2_63061898	-			
rs162501621	5	59447744	-:G>C	S5_59447744	-			
rs162501781	3	58933359	-:A>G	S3_58933359	-			
rs162502019	8	56501119	-:C>A	S8_56501119	-			
rs162503355	9	3177825	-:A>G	S9_3177825	-			
rs162503373	1	76258479	-:A>G	S1_76258479	-			
rs162505273	9	50181475	-:T>C	S9_50181475	-			
rs162505632	5	64920286	-:G>C	S5_64920286	-			
rs162505885	9	6165976	-:G>C	S9_6165976	-			
rs162506681	10	6132236	-:A>G	S10_6132236	-			
rs162508279	5	61168342	-:C>G	S5_61168342	-			
rs162508684	6	53399440	-:C>T	S6_53399440	-			
rs162508938	2	62230708	-:A>G	S2_62230708	-			
rs162509158	10	52025946	-:C>T	S10_52025946	-			
rs162509953	7	59056668	-:A>C	S7_59056668	-			
rs162511571	1	79472365	-:C>T	S1_79472365	-			
rs162511574	2	60875810	-:C>A	S2_60875810	-			
rs162511771	10	2395657	-:C>A	S10_2395657	-			
rs162511966	2	16648922	-:G>T	S2_16648922	-			
rs162512025	7	57226099	-:G>A	S7_57226099	-			
rs162512044	2	69970686	-:A>G	S2_69970686	-			
rs162512177	2	59792649	-:G>A	S2_59792649	-			
rs162512897	6	43671858	-:C>G	S6_43671858	-			
rs162513473	1	50298049	-:T>G	S1_50298049	-			
rs162514315	4	12028146	-:C>G	S4_12028146	-			
rs162514554	6	49569979	-:C>T	S6_49569979	-			
rs162514814	4	3714894	-:T>C	S4_3714894	-			
rs162514902	6	47101054	-:T>A	S6_47101054	-			
rs162515395	3	58653318	-:A>G	S3_58653318	-			
rs162516098	3	8081313	-:A>G	S3_8081313	-			

rs162516110	2	8702160	-:A>G	S2_8702160	-		
rs162516254	2	56966078	-:T>G	S2_56966078	-		
rs162516336	6	50307500	-:G>A	S6_50307500	-		
rs162516495	7	60853629	-:T>C	S7_60853629	-		
rs162518149	2	5384574	-:C>T	S2_5384574	-		
rs162518288	9	46060760	-:T>C	S9_46060760	-		
rs162518328	5	40774458	-:A>G	S5_40774458	-		
rs162518604	2	64144783	-:T>C	S2_64144783	-		
rs162518967	2	3147710	-:G>T	S2_3147710	-		
rs162519430	5	67433963	-:C>G	S5_67433963	-		
rs162519645	2	61118384	-:T>G	S2_61118384	-		
rs162520263	9	21251419	-:C>T	S9_21251419	-		
rs162520322	1	67303950	-:G>C	S1_67303950	-		
rs162521461	6	57643404	-:A>G	S6_57643404	-		
rs162521618	5	17753373	-:C>T	S5_17753373	-		
rs162522115	6	52826386	-:A>G	S6_52826386	-		
rs162522283	3	13716353	-:A>T	S3_13716353	-		
rs162522922	1	6131258	-:G>A	S1_6131258	-		
rs162523116	9	15337777	-:T>C	S9_15337777	-		
rs162523742	1	73835707	-:C>G	S1_73835707	-		
rs162523971	6	1925880	-:C>T	S6_1925880	-		
rs162523997	7	7826308	-:T>C	S7_7826308	-		
rs162525107	9	52824004	-:G>C	S9_52824004	-		
rs162526021	9	3558565	-:A>G	S9_3558565	-		
rs162526368	4	63211680	-:A>C	S4_63211680	-		
rs162526627	10	54283242	-:G>C	S10_54283242	-		
rs162527186	3	15615228	-:G>A	S3_15615228	-		
rs162527301	4	60890518	-:C>T	S4_60890518	-		
rs162527673	2	6458993	-:A>G	S2_6458993	-		
rs162527768	3	5151643	-:A>T	S3_5151643	-		
rs162528542	2	66011792	-:C>T	S2_66011792	-		
rs162528753	4	59168954	-:G>A	S4_59168954	-		
rs162529216	10	2640803	-:C>T	S10_2640803	-		
rs162530821	3	55304645	-:T>C	S3_55304645	-		
rs162530852	10	4811956	-:T>C	S10_4811956	-		
rs162530899	9	53113844	-:G>T	S9_53113844	-		
rs162531036	4	1608134	-:A>C	S4_1608134	-		
rs162531402	10	51415321	-:G>A	S10_51415321	-		
rs162531555	9	8568106	-:G>C	S9_8568106	-		
rs162531606	5	17553897	-:T>A	S5_17553897	-		
rs162532016	6	46045129	-:G>A	S6_46045129	-		
rs162532541	3	59352048	-:T>C	S3_59352048	-		
rs162533894	2	70591486	-:C>T	S2_70591486	-		

rs162534617	10	8575442	-:T>C	S10_8575442	-		
rs162536419	8	59673167	-:G>A	S8_59673167	-		
rs162537358	8	15936480	-:T>G	S8_15936480	-		
rs162537734	5	7076566	-:T>A	S5_7076566	-		
rs162538136	6	47562464	-:C>T	S6_47562464	-		
rs162539272	9	52381116	-:C>T	S9_52381116	-		
rs162540393	3	70935842	-:G>C	S3_70935842	-		
rs162540715	9	56197293	-:G>A	S9_56197293	-		
rs162541093	2	69309355	-:G>A	S2_69309355	-		
rs162541156	10	3628821	-:T>C	S10_3628821	-		
rs162541751	5	11114869	-:G>C	S5_11114869	-		
rs162542259	7	62836873	-:T>C	S7_62836873	-		
rs162543131	2	71654434	-:C>T	S2_71654434	-		
rs162543377	2	62691832	-:A>G	S2_62691832	-		
rs162543477	1	12921125	-:A>G	S1_12921125	-		
rs162543624	6	58661066	-:T>A	S6_58661066	-		
rs162543874	6	47104819	-:C>A	S6_47104819	-		
rs162544592	5	62530405	-:C>T	S5_62530405	-		
rs162544667	7	63646257	-:A>G	S7_63646257	-		
rs162544744	9	546216	-:T>G	S9_546216	-		
rs162545388	5	64058943	-:C>G	S5_64058943	-		
rs162545645	7	64331637	-:A>C	S7_64331637	-		
rs162546290	3	8677807	-:T>G	S3_8677807	-		
rs162546348	8	59643388	-:A>G	S8_59643388	-		
rs162546684	10	60330859	-:T>A	S10_60330859	-		
rs162547535	8	60501912	-:A>C	S8_60501912	-		
rs162547808	5	5040994	-:C>G	S5_5040994	-		
rs162548132	2	62132487	-:G>A	S2_62132487	-		
rs162548226	10	9523311	-:T>C	S10_9523311	-		
rs162548491	10	7944484	-:T>G	S10_7944484	-		
rs162548985	9	46312010	-:C>A	S9_46312010	-		
rs162549444	2	10074229	-:G>A	S2_10074229	-		
rs162549609	10	52234429	-:A>G	S10_52234429	-		
rs162549987	7	54307590	-:G>A	S7_54307590	-		
rs162550035	9	51493692	-:C>T	S9_51493692	-		
rs162550510	1	56504908	-:C>T	S1_56504908	-		
rs162551251	4	60979638	-:G>T	S4_60979638	-		
rs162552064	6	54718400	-:A>G	S6_54718400	-		
rs162552227	3	59803663	-:A>G	S3_59803663	-		
rs162552994	8	58185874	-:A>G	S8_58185874	-		
rs162553426	7	14021752	-:T>A	S7_14021752	-		
rs162554347	3	9841973	-:G>A	S3_9841973	-		
rs162554452	9	49414540	-:A>G	S9_49414540	-		

rs162554548	10	1280555	-:G>A	S10_1280555	-		
rs162555262	2	76853498	-:G>A	S2_76853498	-		
rs162555543	8	2535992	-:C>T	S8_2535992	-		
rs162555614	6	49407775	-:A>T	S6_49407775	-		
rs162556295	2	6027251	-:C>G	S2_6027251	-		
rs162557373	2	20700849	-:G>C	S2_20700849	-		
rs162557666	5	66622578	-:A>G	S5_66622578	-		
rs162558370	10	57232334	-:T>C	S10_57232334	-		
rs162558787	10	57750536	-:A>T	S10_57750536	-		
rs162558852	1	53472226	-:G>A	S1_53472226	-		
rs162559548	6	49545675	-:G>A	S6_49545675	-		
rs162559749	2	61802625	-:T>C	S2_61802625	-		
rs162559978	9	50014845	-:G>T	S9_50014845	-		
rs162561500	6	47572337	-:C>G	S6_47572337	-		
rs162561884	9	56345091	-:C>G	S9_56345091	-		
rs162562325	9	41088398	-:C>T	S9_41088398	-		
rs162563167	7	58580597	-:G>T	S7_58580597	-		
rs162564191	3	60400141	-:A>G	S3_60400141	-		
rs162564498	6	30982177	-:C>T	S6_30982177	-		
rs162564506	7	5931984	-:T>C	S7_5931984	-		
rs162565217	5	63876151	-:A>G	S5_63876151	-		
rs162565488	10	40201117	-:T>C	S10_40201117	-		
rs162566293	5	68963850	-:T>A	S5_68963850	-		
rs162566408	8	7766092	-:G>T	S8_7766092	-		
rs162567295	6	256323	-:G>C	S6_256323	-		
rs162567471	8	60627561	-:C>T	S8_60627561	-		
rs162567475	2	17760736	-:G>A	S2_17760736	-		
rs162568851	6	42122367	-:A>T	S6_42122367	-		
rs162569730	10	51937712	-:G>C	S10_51937712	-		
rs162570006	5	2340804	-:A>G	S5_2340804	-		
rs162570134	3	69847330	-:C>A	S3_69847330	-		
rs162570704	6	55041743	-:T>A	S6_55041743	-		
rs162571090	1	328467	-:A>C	S1_328467	-		
rs162571109	4	67712021	-:A>G	S4_67712021	-		
rs162571244	8	59005120	-:A>G	S8_59005120	-		
rs162571394	6	5575659	-:T>C	S6_5575659	-		
rs162571722	1	3751888	-:C>T	S1_3751888	-		
rs162573547	2	59718245	-:G>C	S2_59718245	-		
rs162573948	3	45554013	-:A>G	S3_45554013	-		
rs162574182	9	55093999	-:T>C	S9_55093999	-		
rs162574539	4	54041657	-:G>A	S4_54041657	-		
rs162574954	2	11786205	-:A>G	S2_11786205	-		
rs162574974	4	61508017	-:G>A	S4_61508017	-		

rs162575230	6	44604637	-:C>T	S6_44604637	-			
rs162575348	3	4997348	-:T>C	S3_4997348	-			
rs162575416	9	21314485	-:A>G	S9_21314485	-			
rs162575697	9	47970834	-:C>A	S9_47970834	-			
rs162575780	4	60843537	-:G>A	S4_60843537	-			
rs162575872	2	71325290	-:C>T	S2_71325290	-			
rs162576970	2	46861490	-:T>C	S2_46861490	-			
rs162577107	3	68956518	-:C>T	S3_68956518	-			
rs162577171	7	8646573	-:A>G	S7_8646573	-			
rs162577374	8	4279660	-:G>A	S8_4279660	-			
rs162577392	4	55800255	-:A>C	S4_55800255	-			
rs162577412	6	45675310	-:C>T	S6_45675310	-			
rs162577989	2	61654770	-:T>A	S2_61654770	-			
rs162578523	4	53281321	-:G>T	S4_53281321	-			
rs162578563	10	5628553	-:A>G	S10_5628553	-			
rs162578799	1	60644333	-:G>A	S1_60644333	-			
rs162578951	4	2210241	-:C>G	S4_2210241	-			
rs162579879	10	8032074	-:A>C	S10_8032074	-			
rs162580032	3	3087844	-:T>C	S3_3087844	-			
rs162580677	3	47848764	-:C>T	S3_47848764	-			
rs162582194	1	7026140	-:G>C	S1_7026140	-			
rs162583181	2	62996588	-:T>G	S2_62996588	-			
rs162583262	1	17712302	-:C>G	S1_17712302	-			
rs162584023	6	46128631	-:C>G	S6_46128631	-			
rs162584079	5	69804013	-:A>T	S5_69804013	-			
rs162584578	3	69405187	-:G>T	S3_69405187	-			
rs162585305	10	55090047	-:C>T	S10_55090047	-			
rs162586083	10	46020547	-:G>A	S10_46020547	-			
rs162586087	6	29347909	-:C>T	S6_29347909	-			
rs162586128	5	68185691	-:A>G	S5_68185691	-			
rs162587049	3	68367370	-:A>C	S3_68367370	-			
rs162587257	10	18443591	-:C>G	S10_18443591	-			
rs162587423	2	59708474	-:C>G	S2_59708474	-			
rs162588041	3	65906476	-:C>A	S3_65906476	-			
rs162588145	4	10628604	-:T>G	S4_10628604	-			
rs162588368	10	9992998	-:C>T	S10_9992998	-			
rs162588751	2	73041735	-:G>T	S2_73041735	-			
rs162589126	3	70517701	-:G>A	S3_70517701	-			
rs162589436	1	65255277	-:G>C	S1_65255277	-			
rs162589474	1	7375009	-:C>T	S1_7375009	-			
rs162589856	7	39095683	-:C>T	S7_39095683	-			
rs162590332	8	60241674	-:A>G	S8_60241674	-			
rs162590351	4	53008957	-:T>C	S4_53008957	-			

rs162590710	1	80524874	-:C>G	S1_80524874	-		
rs162590917	4	55048481	-:C>G	S4_55048481	-		
rs162591408	8	3369623	-:G>A	S8_3369623	-		
rs162591445	4	5330422	-:A>C	S4_5330422	-		
rs162591511	7	62232054	-:T>A	S7_62232054	-		
rs162591902	5	2200635	-:C>T	S5_2200635	-		
rs162592309	2	17710088	-:T>A	S2_17710088	-		
rs162592504	6	60560878	-:T>G	S6_60560878	-		
rs162592564	2	2728844	-:T>A	S2_2728844	-		
rs162592976	3	6692752	-:G>T	S3_6692752	-		
rs162593337	9	51454603	-:A>G	S9_51454603	-		
rs162593342	8	60860587	-:C>T	S8_60860587	-		
rs162593519	1	66827763	-:A>G	S1_66827763	-		
rs162593585	1	825853	-:C>A	S1_825853	-		
rs162594287	7	147787	-:C>T	S7_147787	-		
rs162594527	10	59672314	-:A>T	S10_59672314	-		
rs162595028	9	1646345	-:A>G	S9_1646345	-		
rs162595886	10	56885983	-:G>T	S10_56885983	-		
rs162595936	1	51888194	-:G>T	S1_51888194	-		
rs162596453	2	62972749	-:G>A	S2_62972749	-		
rs162596460	4	67362524	-:G>A	S4_67362524	-		
rs162596845	9	4817855	-:G>C	S9_4817855	-		
rs162596966	4	58929013	-:T>G	S4_58929013	-		
rs162597231	5	64101389	-:G>A	S5_64101389	-		
rs162597322	3	71458504	-:A>T	S3_71458504	-		
rs162597386	8	57569054	-:G>A	S8_57569054	-		
rs162597612	5	41431134	-:A>C	S5_41431134	-		
rs162597788	8	55934571	-:A>G	S8_55934571	-		
rs162597817	7	59404098	-:A>C	S7_59404098	-		
rs162597959	4	6054210	-:G>C	S4_6054210	-		
rs162598362	1	29776420	-:T>G	S1_29776420	-		
rs162598482	2	75456079	-:A>G	S2_75456079	-		
rs162598574	1	59687975	-:A>G	S1_59687975	-		
rs162598829	6	44050445	-:G>A	S6_44050445	-		
rs162598893	5	62386671	-:C>T	S5_62386671	-		
rs162598985	5	50727386	-:A>T	S5_50727386	-		
rs162600355	6	51538707	-:T>C	S6_51538707	-		
rs162600428	8	61563181	-:G>A	S8_61563181	-		
rs162600987	5	6003124	-:A>G	S5_6003124	-		
rs162601008	10	58739997	-:G>C	S10_58739997	-		
rs162601885	1	78496212	-:G>T	S1_78496212	-		
rs162601932	5	68814273	-:T>G	S5_68814273	-		
rs162601983	2	8708044	-:C>T	S2_8708044	-		

rs162602128	9	9647879	-:A>G	S9_9647879	-		
rs162602315	1	61289282	-:G>C	S1_61289282	-		
rs162602702	7	65177282	-:A>G	S7_65177282	-		
rs162603319	9	6773974	-:G>C	S9_6773974	-		
rs162603545	8	3046448	-:C>G	S8_3046448	-		
rs162603633	7	54789016	-:C>G	S7_54789016	-		
rs162603643	8	52227363	-:T>C	S8_52227363	-		
rs162604468	3	60581898	-:G>A	S3_60581898	-		
rs162604505	2	56332220	-:C>G	S2_56332220	-		
rs162604937	1	17285880	-:C>T	S1_17285880	-		
rs162605044	2	3042598	-:G>C	S2_3042598	-		
rs162605344	10	50794228	-:A>G	S10_50794228	-		
rs162605413	2	14749096	-:G>C	S2_14749096	-		
rs162605595	8	61278748	-:G>A	S8_61278748	-		
rs162605597	10	7262590	-:T>C	S10_7262590	-		
rs162606261	10	45995646	-:T>C	S10_45995646	-		
rs162606822	7	38852322	-:T>C	S7_38852322	-		
rs162607114	6	47959714	-:T>C	S6_47959714	-		
rs162607465	2	71570208	-:A>G	S2_71570208	-		
rs162607719	10	49923377	-:G>T	S10_49923377	-		
rs162608594	1	75620010	-:C>T	S1_75620010	-		
rs162609689	5	39115252	-:T>A	S5_39115252	-		
rs162609805	2	14172689	-:C>G	S2_14172689	-		
rs162611219	5	66935973	-:G>C	S5_66935973	-		
rs162611481	6	43637982	-:C>T	S6_43637982	-		
rs162612356	2	66955986	-:C>T	S2_66955986	-		
rs162613534	8	2182521	-:C>T	S8_2182521	-		
rs162613556	2	72412281	-:G>A	S2_72412281	-		
rs162613866	4	60173722	-:C>A	S4_60173722	-		
rs162613896	5	57167284	-:C>T	S5_57167284	-		
rs162614215	10	17467837	-:A>G	S10_17467837	-		
rs162614728	8	62357171	-:G>A	S8_62357171	-		
rs162615380	3	60783841	-:A>G	S3_60783841	-		
rs162615888	10	52084581	-:C>T	S10_52084581	-		
rs162616028	3	69210380	-:A>T	S3_69210380	-		
rs162616041	9	49367419	-:C>A	S9_49367419	-		
rs162616262	10	45829247	-:G>A	S10_45829247	-		
rs162616277	1	12247789	-:A>C	S1_12247789	-		
rs162616612	3	70581271	-:G>C	S3_70581271	-		
rs162618363	7	9684645	-:G>A	S7_9684645	-		
rs162618898	2	74592550	-:G>A	S2_74592550	-		
rs162618924	3	64967486	-:G>C	S3_64967486	-		
rs162619632	1	19688299	-:A>G	S1_19688299	-		

rs162620058	2	60853828	-:A>G	S2_60853828	-		
rs162620376	6	47055810	-:C>T	S6_47055810	-		
rs162620458	2	57728127	-:C>A	S2_57728127	-		
rs162620779	7	54708458	-:C>T	S7_54708458	-		
rs162620922	6	47693927	-:A>C	S6_47693927	-		
rs162621965	7	60248577	-:C>G	S7_60248577	-		
rs162622089	10	55586987	-:C>A	S10_55586987	-		
rs162622829	4	27290507	-:A>G	S4_27290507	-		
rs162623467	4	8919303	-:T>G	S4_8919303	-		
rs162623522	10	58070085	-:C>T	S10_58070085	-		
rs162623803	1	52747335	-:C>T	S1_52747335	-		
rs162624310	4	4083807	-:C>A	S4_4083807	-		
rs162625917	3	13359317	-:G>T	S3_13359317	-		
rs162626556	6	6450331	-:G>A	S6_6450331	-		
rs162626650	7	60356421	-:T>C	S7_60356421	-		
rs162627367	3	70798551	-:C>T	S3_70798551	-		
rs162628527	10	12294048	-:T>G	S10_12294048	-		
rs162629299	2	10821575	-:G>A	S2_10821575	-		
rs162629335	3	9703273	-:A>C	S3_9703273	-		
rs162630333	7	13257987	-:G>A	S7_13257987	-		
rs162630454	1	79576288	-:T>C	S1_79576288	-		
rs162632752	4	1099100	-:C>T	S4_1099100	-		
rs162633148	1	11562582	-:A>G	S1_11562582	-		
rs162633470	9	51633653	-:C>A	S9_51633653	-		
rs162633989	3	55432695	-:G>A	S3_55432695	-		
rs162634421	8	39466000	-:G>C	S8_39466000	-		
rs162634663	6	51520792	-:T>G	S6_51520792	-		
rs162635451	6	51449251	-:T>C	S6_51449251	-		
rs162635939	7	53013212	-:T>C	S7_53013212	-		
rs162636427	4	53381294	-:C>G	S4_53381294	-		
rs162637250	1	5073589	-:A>G	S1_5073589	-		
rs162637976	10	21129436	-:A>G	S10_21129436	-		
rs162638196	6	60086312	-:A>C	S6_60086312	-		
rs162638433	3	61669173	-:G>A	S3_61669173	-		
rs162638519	5	5294544	-:T>C	S5_5294544	-		
rs162638712	8	2938859	-:A>G	S8_2938859	-		
rs162638835	4	50735967	-:C>T	S4_50735967	-		
rs162640566	8	615054	-:C>G	S8_615054	-		
rs162641104	2	63162315	-:A>G	S2_63162315	-		
rs162641258	6	59705847	-:C>G	S6_59705847	-		
rs162641641	9	12575744	-:G>A	S9_12575744	-		
rs162642289	2	75430273	-:T>G	S2_75430273	-		
rs162642835	10	7264843	-:A>G	S10_7264843	-		

rs162642881	1	13775623	-:A>G	S1_13775623	-		
rs162642921	10	53951438	-:C>A	S10_53951438	-		
rs162643040	6	61062942	-:T>C	S6_61062942	-		
rs162643131	6	50690547	-:G>T	S6_50690547	-		
rs162643572	4	2082864	-:C>T	S4_2082864	-		
rs162643936	2	8423718	-:G>T	S2_8423718	-		
rs162643992	4	50906156	-:C>G	S4_50906156	-		
rs162644092	1	58766380	-:C>T	S1_58766380	-		
rs162644612	3	73334633	-:C>T	S3_73334633	-		
rs162644748	8	59389073	-:A>G	S8_59389073	-		
rs162645233	10	53069219	-:T>C	S10_53069219	-		
rs162645306	8	3133512	-:A>G	S8_3133512	-		
rs162645647	4	56363457	-:A>C	S4_56363457	-		
rs162645959	2	16852931	-:T>G	S2_16852931	-		
rs162647486	9	7342632	-:C>T	S9_7342632	-		
rs162647639	10	58553403	-:A>T	S10_58553403	-		
rs162647800	6	54715540	-:G>A	S6_54715540	-		
rs162648151	6	45670817	-:C>G	S6_45670817	-		
rs162649080	1	4412676	-:A>T	S1_4412676	-		
rs162649925	3	62407264	-:A>G	S3_62407264	-		
rs162650396	1	58037903	-:A>G	S1_58037903	-		
rs162650752	8	17983641	-:G>C	S8_17983641	-		
rs162650759	6	52835444	-:G>A	S6_52835444	-		
rs162651223	9	50751693	-:C>T	S9_50751693	-		
rs162651511	3	13769864	-:T>G	S3_13769864	-		
rs162651593	1	77252048	-:T>G	S1_77252048	-		
rs162651615	2	63216470	-:A>G	S2_63216470	-		
rs162651933	6	50213600	-:A>C	S6_50213600	-		
rs162652404	1	65670435	-:A>G	S1_65670435	-		
rs162652667	6	42578668	-:T>C	S6_42578668	-		
rs162653110	6	47959762	-:C>G	S6_47959762	-		
rs162653140	7	55566988	-:T>C	S7_55566988	-		
rs162653218	9	361240	-:G>A	S9_361240	-		
rs162653279	8	57539701	-:T>C	S8_57539701	-		
rs162653340	5	71729971	-:G>A	S5_71729971	-		
rs162653684	3	58666607	-:G>A	S3_58666607	-		
rs162654038	9	8145089	-:A>G	S9_8145089	-		
rs162654371	7	3195842	-:C>G	S7_3195842	-		
rs162654738	1	13463791	-:C>T	S1_13463791	-		
rs162655200	4	56539735	-:C>T	S4_56539735	-		
rs162655772	1	21838914	-:T>C	S1_21838914	-		
rs162655834	3	70105659	-:C>T	S3_70105659	-		
rs162656487	1	59458791	-:A>G	S1_59458791	-		

rs162657302	4	4642875	-:T>C	S4_4642875	-		
rs162657464	3	1739133	-:G>A	S3_1739133	-		
rs162657564	3	53901112	-:A>G	S3_53901112	-		
rs162657903	6	42344102	-:G>C	S6_42344102	-		
rs162658489	6	47073850	-:G>A	S6_47073850	-		
rs162658849	9	56521102	-:G>A	S9_56521102	-		
rs162658931	3	69704028	-:T>C	S3_69704028	-		
rs162659572	1	79621658	-:T>C	S1_79621658	-		
rs162659908	2	63059485	-:T>G	S2_63059485	-		
rs162660002	2	23048781	-:G>C	S2_23048781	-		
rs162660237	2	73160854	-:G>C	S2_73160854	-		
rs162660662	7	2748578	-:T>C	S7_2748578	-		
rs162660918	1	76994951	-:G>C	S1_76994951	-		
rs162661201	1	68089858	-:C>T	S1_68089858	-		
rs162661553	3	70989914	-:G>A	S3_70989914	-		
rs162661591	1	75978543	-:T>C	S1_75978543	-		
rs162661800	4	9881140	-:A>G	S4_9881140	-		
rs162662488	9	18426157	-:T>C	S9_18426157	-		
rs162662595	3	6089452	-:T>G	S3_6089452	-		
rs162663082	1	72952996	-:C>T	S1_72952996	-		
rs162663453	1	25687179	-:G>A	S1_25687179	-		
rs162665465	3	11682525	-:G>T	S3_11682525	-		
rs162665469	4	62188563	-:C>A	S4_62188563	-		
rs162666041	3	70640369	-:C>G	S3_70640369	-		
rs162666872	3	59618632	-:A>G	S3_59618632	-		
rs162667192	10	17629879	-:C>A	S10_17629879	-		
rs162667542	10	53091752	-:G>A	S10_53091752	-		
rs162667591	5	61407903	-:G>C	S5_61407903	-		
rs162667708	10	57295952	-:A>T	S10_57295952	-		
rs162667725	1	67610072	-:T>G	S1_67610072	-		
rs162667838	8	59352296	-:C>T	S8_59352296	-		
rs162668138	1	9225461	-:A>C	S1_9225461	-		
rs162668896	8	54223569	-:A>G	S8_54223569	-		
rs162669197	3	70146945	-:A>G	S3_70146945	-		
rs162669960	9	11940753	-:A>C	S9_11940753	-		
rs162669968	8	57569318	-:A>G	S8_57569318	-		
rs162670015	2	1055876	-:T>G	S2_1055876	-		
rs162670875	5	17172975	-:C>G	S5_17172975	-		
rs162671573	3	8579079	-:G>A	S3_8579079	-		
rs162671798	2	69122029	-:T>G	S2_69122029	-		
rs162672206	5	71519452	-:C>G	S5_71519452	-		
rs162672537	1	14281325	-:C>G	S1_14281325	-		
rs162672961	10	5550715	-:T>C	S10_5550715	-		

rs162673001	8	1839095	-:T>C	S8_1839095	-		
rs162673255	1	77966174	-:C>T	S1_77966174	-		
rs162673362	4	67970776	-:C>T	S4_67970776	-		
rs162673589	8	1699232	-:T>C	S8_1699232	-		
rs162673820	9	6277941	-:C>T	S9_6277941	-		
rs162673936	1	55830089	-:T>C	S1_55830089	-		
rs162674108	2	60775899	-:G>A	S2_60775899	-		
rs162674138	8	57442354	-:C>T	S8_57442354	-		
rs162674180	8	2472931	-:C>G	S8_2472931	-		
rs162675158	7	5275814	-:T>A	S7_5275814	-		
rs162675179	1	8130800	-:T>A	S1_8130800	-		
rs162675294	8	53861070	-:G>C	S8_53861070	-		
rs162675328	3	67939360	-:A>G	S3_67939360	-		
rs162675643	6	46064034	-:A>T	S6_46064034	-		
rs162676450	7	3637517	-:G>A	S7_3637517	-		
rs162676848	2	75994648	-:G>C	S2_75994648	-		
rs162676997	10	15778385	-:A>G	S10_15778385	-		
rs162677353	3	57291467	-:C>T	S3_57291467	-		
rs162677550	4	2084214	-:C>T	S4_2084214	-		
rs162678308	7	58747355	-:A>T	S7_58747355	-		
rs162678871	4	56916270	-:C>G	S4_56916270	-		
rs162679123	4	2652172	-:A>G	S4_2652172	-		
rs162680158	8	5652481	-:C>G	S8_5652481	-		
rs162681898	1	2723780	-:G>A	S1_2723780	-		
rs162681932	10	55375263	-:C>T	S10_55375263	-		
rs162681972	3	10392390	-:C>G	S3_10392390	-		
rs162682152	5	1375985	-:G>A	S5_1375985	-		
rs162682393	6	42047270	-:T>A	S6_42047270	-		
rs162682804	3	59501629	-:T>C	S3_59501629	-		
rs162682829	6	55480761	-:G>T	S6_55480761	-		
rs162683791	9	59323860	-:T>A	S9_59323860	-		
rs162685212	4	2241435	-:A>G	S4_2241435	-		
rs162685424	3	1486771	-:A>G	S3_1486771	-		
rs162685638	1	58169716	-:C>T	S1_58169716	-		
rs162687064	5	71726777	-:T>A	S5_71726777	-		
rs162687484	4	66204568	-:G>A	S4_66204568	-		
rs162688785	9	15601316	-:T>C	S9_15601316	-		
rs162688941	7	6530847	-:T>C	S7_6530847	-		
rs162689514	9	58540112	-:T>G	S9_58540112	-		
rs162689524	2	8432703	-:G>A	S2_8432703	-		
rs162689527	8	10850798	-:G>C	S8_10850798	-		
rs162690186	7	64618839	-:G>A	S7_64618839	-		
rs162690346	2	20573567	-:A>T	S2_20573567	-		

rs266259710	1	76002582	-:C>A	S1_76002582	-		
rs266260016	2	72659075	-:G>A	S2_72659075	-		
rs266264950	1	17391798	-:A>G	S1_17391798	-		
rs266266496	3	64849395	-:T>C	S3_64849395	-		
rs266266857	5	19512281	-:C>A	S5_19512281	-		
rs266268749	7	6584892	-:G>C	S7_6584892	-		
rs266269642	2	68829674	-:G>T	S2_68829674	-		
rs266269833	5	21004861	-:C>T	S5_21004861	-		
rs266270537	4	61636634	-:A>G	S4_61636634	-		
rs266271594	2	60059537	-:A>G	S2_60059537	-		
rs266272348	3	61585959	-:A>G	S3_61585959	-		
rs266273126	5	64801769	-:T>C	S5_64801769	-		
rs266275672	3	56526523	-:T>C	S3_56526523	-		
rs266276203	3	6081428	-:T>C	S3_6081428	-		
rs266277441	6	59264648	-:A>G	S6_59264648	-		
rs266278933	1	50829052	-:C>T	S1_50829052	-		
rs266279528	3	8739702	-:A>G	S3_8739702	-		
rs266281327	9	44684778	-:A>G	S9_44684778	-		
rs266283444	5	9537077	-:T>C	S5_9537077	-		
rs266283503	3	66560521	-:A>T	S3_66560521	-		
rs266286232	1	67493247	-:T>A	S1_67493247	-		
rs266287587	10	18068593	-:G>T	S10_18068593	-		
rs266287701	10	38891445	-:A>C	S10_38891445	-		
rs266288772	3	58939558	-:C>A	S3_58939558	-		
rs266288961	6	47235150	-:C>A	S6_47235150	-		
rs266289626	10	57794335	-:C>T	S10_57794335	-		
rs266291381	4	61421187	-:C>G	S4_61421187	-		
rs266292668	3	15947278	-:T>C	S3_15947278	-		
rs266292741	9	20928365	-:T>G	S9_20928365	-		
rs266294780	9	56463758	-:A>C	S9_56463758	-		
rs266296441	10	9810491	-:C>G	S10_9810491	-		
rs266297198	9	42349231	-:G>A	S9_42349231	-		
rs266297305	9	2984357	-:A>G	S9_2984357	-		
rs266297848	1	6666029	-:T>G	S1_6666029	-		
rs266299040	1	6889520	-:T>C	S1_6889520	-		
rs266299854	5	11366861	-:G>C	S5_11366861	-		
rs266300675	4	3692100	-:A>C	S4_3692100	-		
rs266301105	4	4638445	-:T>C	S4_4638445	-		
rs266302852	7	326599	-:A>G	S7_326599	-		
rs266303102	1	67400001	-:G>C	S1_67400001	-		
rs266303409	9	49032281	-:C>G	S9_49032281	-		
rs266306626	5	14529506	-:C>T	S5_14529506	-		
rs266308728	6	6110410	-:T>C	S6_6110410	-		

rs266309526	3	52787725	-:T>G	S3_52787725	-		
rs266309813	10	9280539	-:T>A	S10_9280539	-		
rs266311946	2	11317066	-:T>C	S2_11317066	-		
rs266312041	2	57680557	-:T>G	S2_57680557	-		
rs266313708	5	38427281	-:A>G	S5_38427281	-		
rs266316255	3	1441117	-:A>G	S3_1441117	-		
rs266317759	7	62752351	-:G>T	S7_62752351	-		
rs266317830	2	75563691	-:G>A	S2_75563691	-		
rs266318981	9	54723632	-:T>C	S9_54723632	-		
rs266321064	7	64827187	-:A>G	S7_64827187	-		
rs266322772	1	50829957	-:T>C	S1_50829957	-		
rs266325892	6	40465470	-:T>G	S6_40465470	-		
rs266325944	2	61091644	-:G>C	S2_61091644	-		
rs266327298	3	56950540	-:T>G	S3_56950540	-		
rs266329348	3	51347860	-:G>A	S3_51347860	-		
rs266329875	2	46939184	-:A>G	S2_46939184	-		
rs266331292	10	18073035	-:A>T	S10_18073035	-		
rs266332156	2	8262851	-:G>C	S2_8262851	-		
rs266333052	8	51761985	-:T>G	S8_51761985	-		
rs266337535	4	2674426	-:G>A	S4_2674426	-		
rs266341942	4	1778425	-:C>T	S4_1778425	-		
rs266342299	7	60573720	-:A>C	S7_60573720	-		
rs266342456	8	58903816	-:G>C	S8_58903816	-		
rs266346455	4	6417758	-:G>T	S4_6417758	-		
rs266348857	5	12084207	-:A>G	S5_12084207	-		
rs266349831	7	12015926	-:C>G	S7_12015926	-		
rs266350138	5	56744127	-:G>A	S5_56744127	-		
rs266351445	4	8721827	-:C>G	S4_8721827	-		
rs266354046	5	62916140	-:A>G	S5_62916140	-		
rs266354296	2	62993237	-:C>T	S2_62993237	-		
rs266354795	1	64361916	-:G>A	S1_64361916	-		
rs266354981	4	44067909	-:A>G	S4_44067909	-		
rs266355988	3	71573506	-:A>C	S3_71573506	-		
rs266357864	9	16272219	-:C>T	S9_16272219	-		
rs266358298	1	16701274	-:A>G	S1_16701274	-		
rs266359419	4	50575672	-:A>T	S4_50575672	-		
rs266360833	9	12575258	-:T>G	S9_12575258	-		
rs266361362	9	5519208	-:A>T	S9_5519208	-		
rs266361862	8	5480369	-:T>G	S8_5480369	-		
rs266363499	3	71042084	-:G>A	S3_71042084	-		
rs266363759	8	60488372	-:A>G	S8_60488372	-		
rs266364004	6	26390356	-:C>T	S6_26390356	-		
rs266366460	8	60819122	-:T>C	S8_60819122	-		

rs266367873	9	51973123	-:C>T	S9_51973123	-		
rs266369887	3	58661379	-:G>A	S3_58661379	-		
rs266369904	6	49765295	-:G>A	S6_49765295	-		
rs266370364	10	57850287	-:C>T	S10_57850287	-		
rs266370910	5	52058372	-:C>G	S5_52058372	-		
rs266373238	1	59823957	-:G>C	S1_59823957	-		
rs266374300	4	61204247	-:A>T	S4_61204247	-		
rs266376048	2	9778303	-:C>G	S2_9778303	-		
rs266376286	1	64159639	-:G>T	S1_64159639	-		
rs266377791	6	60726944	-:G>A	S6_60726944	-		
rs266377849	8	4051690	-:A>T	S8_4051690	-		
rs266378106	7	65355397	-:A>G	S7_65355397	-		
rs266378493	3	61484178	-:G>T	S3_61484178	-		
rs266381101	8	6118586	-:G>A	S8_6118586	-		
rs266382586	3	7933165	-:T>C	S3_7933165	-		
rs266382793	7	2907701	-:A>G	S7_2907701	-		
rs266383491	4	54086420	-:T>C	S4_54086420	-		
rs266383531	10	59035419	-:G>A	S10_59035419	-		
rs266384717	9	6074633	-:C>A	S9_6074633	-		
rs266384911	7	8742420	-:G>A	S7_8742420	-		
rs266385805	3	52794964	-:C>T	S3_52794964	-		
rs266387324	9	2886889	-:T>C	S9_2886889	-		
rs266388157	7	63643873	-:A>G	S7_63643873	-		
rs266390108	4	53209576	-:G>A	S4_53209576	-		
rs266390578	6	43657157	-:C>T	S6_43657157	-		
rs266391004	3	65626321	-:T>C	S3_65626321	-		
rs266391246	9	47596511	-:T>C	S9_47596511	-		
rs266391521	2	58861091	-:C>A	S2_58861091	-		
rs266391542	4	59507349	-:T>C	S4_59507349	-		
rs266391597	7	50460454	-:A>G	S7_50460454	-		
rs266392235	9	4377589	-:A>C	S9_4377589	-		
rs266392486	4	66431272	-:C>T	S4_66431272	-		
rs266392599	7	39914624	-:C>A	S7_39914624	-		
rs266393596	3	58661467	-:T>G	S3_58661467	-		
rs266394011	2	76427244	-:G>A	S2_76427244	-		
rs266394133	8	62367132	-:T>A	S8_62367132	-		
rs266395890	7	56493308	-:A>G	S7_56493308	-		
rs266396918	1	3718398	-:G>C	S1_3718398	-		
rs266397021	4	6830932	-:T>C	S4_6830932	-		
rs266397415	7	2468906	-:T>C	S7_2468906	-		
rs266398120	8	55571192	-:T>C	S8_55571192	-		
rs266400688	2	58331085	-:T>C	S2_58331085	-		
rs266400822	9	55076439	-:T>C	S9_55076439	-		

rs266400835	6	2396650	-:G>A	S6_2396650	-		
rs266401317	5	3658502	-:G>T	S5_3658502	-		
rs266403147	9	3905123	-:G>A	S9_3905123	-		
rs266403217	3	69698464	-:T>C	S3_69698464	-		
rs266403863	10	58729267	-:G>A	S10_58729267	-		
rs266404034	6	30441715	-:C>A	S6_30441715	-		
rs266407989	8	7284400	-:G>C	S8_7284400	-		
rs266408854	1	66753557	-:G>A	S1_66753557	-		
rs266410158	1	15517837	-:A>C	S1_15517837	-		
rs266410345	8	1865498	-:T>G	S8_1865498	-		
rs266410388	4	5055249	-:A>G	S4_5055249	-		
rs266411679	1	13596912	-:G>A	S1_13596912	-		
rs266412455	2	2382977	-:C>A	S2_2382977	-		
rs266413358	5	67329778	-:C>T	S5_67329778	-		
rs266414703	1	1218846	-:T>C	S1_1218846	-		
rs266416969	7	9795378	-:A>G	S7_9795378	-		
rs266420232	6	10566902	-:G>A	S6_10566902	-		
rs266420737	5	65038525	-:A>G	S5_65038525	-		
rs266423786	3	73485371	-:A>G	S3_73485371	-		
rs266424945	1	71607735	-:G>A	S1_71607735	-		
rs266425304	8	1471082	-:T>G	S8_1471082	-		
rs266428897	10	20239343	-:A>C	S10_20239343	-		
rs266429231	1	78319943	-:C>T	S1_78319943	-		
rs266429288	3	52169120	-:A>T	S3_52169120	-		
rs266431144	1	57949163	-:G>A	S1_57949163	-		
rs266432571	1	60150466	-:A>G	S1_60150466	-		
rs266435334	3	6054184	-:A>G	S3_6054184	-		
rs266435561	3	2966074	-:C>G	S3_2966074	-		
rs266437113	6	43991698	-:A>G	S6_43991698	-		
rs266437269	6	5573833	-:G>A	S6_5573833	-		
rs266443639	3	61379084	-:T>C	S3_61379084	-		
rs266446879	3	6018709	-:T>A	S3_6018709	-		
rs266447351	9	1545573	-:T>G	S9_1545573	-		
rs266447912	8	60185207	-:G>A	S8_60185207	-		
rs266450208	9	8621925	-:C>T	S9_8621925	-		
rs266452755	3	8740753	-:T>A	S3_8740753	-		
rs266452864	6	48605625	-:C>G	S6_48605625	-		
rs266454114	3	9291754	-:C>A	S3_9291754	-		
rs266455468	8	60819058	-:A>G	S8_60819058	-		
rs266456450	1	78063951	-:G>A	S1_78063951	-		
rs266457688	3	62308359	-:T>C	S3_62308359	-		
rs266458088	4	61190110	-:G>A	S4_61190110	-		
rs266459114	3	57615735	-:C>G	S3_57615735	-		

rs266459493	9	15361515	-:G>A	S9_15361515	-		
rs266460349	5	11309581	-:A>T	S5_11309581	-		
rs266462121	5	64643182	-:G>A	S5_64643182	-		
rs266462890	2	56924165	-:G>C	S2_56924165	-		
rs266462956	9	3346746	-:T>G	S9_3346746	-		
rs266464988	5	50895751	-:T>G	S5_50895751	-		
rs266466446	4	52459904	-:T>A	S4_52459904	-		
rs266467027	9	3618727	-:C>G	S9_3618727	-		
rs266467411	3	57258203	-:T>C	S3_57258203	-		
rs266469350	2	5644126	-:T>C	S2_5644126	-		
rs266470889	2	4635532	-:T>A	S2_4635532	-		
rs266471960	3	6793696	-:C>T	S3_6793696	-		
rs266472246	4	12230031	-:T>G	S4_12230031	-		
rs266472804	2	2521455	-:T>C	S2_2521455	-		
rs266473260	3	61654196	-:G>A	S3_61654196	-		
rs266474822	8	1037980	-:T>C	S8_1037980	-		
rs266478177	9	54820637	-:T>C	S9_54820637	-		
rs266479409	5	8331800	-:A>C	S5_8331800	-		
rs266479416	3	61490500	-:A>G	S3_61490500	-		
rs266480617	3	56188180	-:C>G	S3_56188180	-		
rs266480818	3	11870179	-:G>A	S3_11870179	-		
rs266482247	6	50473457	-:A>G	S6_50473457	-		
rs266482428	10	47393530	-:A>C	S10_47393530	-		
rs266482464	5	4223170	-:A>C	S5_4223170	-		
rs266482945	7	539327	-:C>A	S7_539327	-		
rs266483600	7	12011818	-:A>G	S7_12011818	-		
rs266485860	5	8831267	-:T>C	S5_8831267	-		
rs266486091	1	74788741	-:A>G	S1_74788741	-		
rs266486435	2	388603	-:C>T	S2_388603	-		
rs266487292	6	49841826	-:G>A	S6_49841826	-		
rs266488919	2	60383083	-:T>C	S2_60383083	-		
rs266489012	7	5099616	-:A>G	S7_5099616	-		
rs266489562	9	3177907	-:G>C	S9_3177907	-		
rs266489631	2	18316316	-:A>G	S2_18316316	-		
rs266490180	3	52003891	-:A>G	S3_52003891	-		
rs266492853	5	14898462	-:T>C	S5_14898462	-		
rs266495531	10	7171689	-:T>G	S10_7171689	-		
rs266495616	2	3467457	-:T>C	S2_3467457	-		
rs266495805	8	55814710	-:T>A	S8_55814710	-		
rs266495876	8	55522055	-:G>A	S8_55522055	-		
rs266497398	1	19067184	-:G>T	S1_19067184	-		
rs266508005	5	10490547	-:C>T	S5_10490547	-		
rs266508393	1	14620577	-:C>T	S1_14620577	-		

rs266509185	7	53916335	-:G>T	S7_53916335	-		
rs266509346	4	52333778	-:T>C	S4_52333778	-		
rs266509386	4	3886408	-:C>G	S4_3886408	-		
rs266510497	3	45675466	-:T>C	S3_45675466	-		
rs266510553	5	8674350	-:A>G	S5_8674350	-		
rs266511625	8	60947885	-:G>C	S8_60947885	-		
rs266513398	4	52630990	-:C>G	S4_52630990	-		
rs266516371	4	55493852	-:G>A	S4_55493852	-		
rs266516737	3	14132758	-:T>A	S3_14132758	-		
rs266516952	3	60352954	-:G>A	S3_60352954	-		
rs266517147	9	113865	-:G>A	S9_113865	-		
rs266517879	8	2626842	-:A>G	S8_2626842	-		
rs266521106	7	6639284	-:T>G	S7_6639284	-		
rs266522038	1	6518631	-:A>C	S1_6518631	-		
rs266522648	10	18671648	-:C>T	S10_18671648	-		
rs266524349	7	62596766	-:C>G	S7_62596766	-		
rs266524622	10	1981147	-:C>T	S10_1981147	-		
rs266525041	3	55899476	-:C>T	S3_55899476	-		
rs266525657	4	57741537	-:T>C	S4_57741537	-		
rs266527892	2	73941818	-:G>A	S2_73941818	-		
rs266527895	8	46410361	-:G>A	S8_46410361	-		
rs266527981	4	61700918	-:G>T	S4_61700918	-		
rs266529151	1	58989013	-:C>T	S1_58989013	-		
rs266529785	4	53038156	-:C>T	S4_53038156	-		
rs266530431	10	15045057	-:G>C	S10_15045057	-		
rs266531061	10	53616305	-:C>A	S10_53616305	-		
rs266531760	1	17812597	-:C>T	S1_17812597	-		
rs266533744	8	53211227	-:A>T	S8_53211227	-		
rs266535586	4	57035942	-:G>A	S4_57035942	-		
rs266536330	6	49691842	-:G>C	S6_49691842	-		
rs266537739	3	68031538	-:T>C	S3_68031538	-		
rs266537958	10	12258891	-:G>C	S10_12258891	-		
rs266538344	3	11040115	-:A>G	S3_11040115	-		
rs266538884	2	62480636	-:T>C	S2_62480636	-		
rs266539654	9	5959086	-:A>T	S9_5959086	-		
rs266540062	10	11001368	-:C>T	S10_11001368	-		
rs266540298	9	3059895	-:T>A	S9_3059895	-		
rs266541140	8	61052642	-:C>G	S8_61052642	-		
rs266541196	9	1698328	-:C>T	S9_1698328	-		
rs266541215	7	62794520	-:A>G	S7_62794520	-		
rs266542954	8	4414063	-:T>C	S8_4414063	-		
rs266543822	2	8263749	-:C>T	S2_8263749	-		
rs266548488	1	1613105	-:G>A	S1_1613105	-		

rs266551264	3	45681977	-:A>G	S3_45681977	-		
rs266551579	10	21876054	-:T>C	S10_21876054	-		
rs266552836	3	52779518	-:G>A	S3_52779518	-		
rs266553432	5	11064554	-:C>T	S5_11064554	-		
rs266554186	6	59971769	-:G>C	S6_59971769	-		
rs266554193	4	11287103	-:C>T	S4_11287103	-		
rs266554746	6	55815377	-:C>T	S6_55815377	-		
rs266558147	2	14776073	-:G>A	S2_14776073	-		
rs266558779	4	6415464	-:A>C	S4_6415464	-		
rs266558946	9	57771040	-:A>G	S9_57771040	-		
rs266560008	9	25842592	-:T>C	S9_25842592	-		
rs266561628	4	38925815	-:G>A	S4_38925815	-		
rs266564177	7	63998483	-:A>G	S7_63998483	-		
rs266566822	5	54646834	-:G>A	S5_54646834	-		
rs266567728	8	60452395	-:G>C	S8_60452395	-		
rs266569117	1	70812416	-:T>C	S1_70812416	-		
rs266570909	6	6594827	-:A>G	S6_6594827	-		
rs266572066	3	54474091	-:G>A	S3_54474091	-		
rs266572313	7	2922655	-:C>A	S7_2922655	-		
rs266572352	6	27450292	-:T>C	S6_27450292	-		
rs266573066	9	2238328	-:A>G	S9_2238328	-		
rs266573471	7	2354537	-:C>T	S7_2354537	-		
rs266576781	3	55545771	-:A>G	S3_55545771	-		
rs266578171	3	57149191	-:T>C	S3_57149191	-		
rs266579080	8	62084754	-:T>C	S8_62084754	-		
rs266582324	3	60263000	-:G>T	S3_60263000	-		
rs266583208	1	61158547	-:T>C	S1_61158547	-		
rs266584424	3	72027865	-:A>G	S3_72027865	-		
rs266584456	9	404903	-:T>C	S9_404903	-		
rs266584665	9	53224619	-:C>T	S9_53224619	-		
rs266585973	4	10305203	-:G>A	S4_10305203	-		
rs266586484	9	53998046	-:T>C	S9_53998046	-		
rs266587806	7	14135566	-:T>A	S7_14135566	-		
rs266593500	2	11765687	-:A>T	S2_11765687	-		
rs266594826	5	65697673	-:C>A	S5_65697673	-		
rs266595905	4	7138677	-:T>C	S4_7138677	-		
rs266596502	1	3220409	-:C>G	S1_3220409	-		
rs266597668	5	67433894	-:C>G	S5_67433894	-		
rs266600010	4	64403295	-:C>T	S4_64403295	-		
rs266600680	9	3314960	-:T>C	S9_3314960	-		
rs266603623	3	53295639	-:G>A	S3_53295639	-		
rs266606245	7	60752447	-:A>G	S7_60752447	-		
rs266607553	2	68595249	-:G>C	S2_68595249	-		

rs266608665	2	8672470	-:G>C	S2_8672470	-		
rs266608871	3	9953141	-:G>A	S3_9953141	-		
rs266609788	1	15870946	-:T>C	S1_15870946	-		
rs266609847	1	60147635	-:A>G	S1_60147635	-		
rs266611439	1	73062334	-:A>G	S1_73062334	-		
rs266612125	7	60881347	-:G>A	S7_60881347	-		
rs266614562	1	1727150	-:A>G	S1_1727150	-		
rs266616333	10	2186456	-:G>C	S10_2186456	-		
rs266617355	5	4754454	-:G>C	S5_4754454	-		
rs266617592	9	58906485	-:A>C	S9_58906485	-		
rs266618195	1	9881464	-:C>T	S1_9881464	-		
rs266618304	9	12573652	-:A>C	S9_12573652	-		
rs266621378	1	20120607	-:C>G	S1_20120607	-		
rs266621868	10	58946084	-:A>C	S10_58946084	-		
rs266621914	1	72171167	-:C>T	S1_72171167	-		
rs266621941	9	50227800	-:C>G	S9_50227800	-		
rs266621996	1	19407750	-:T>C	S1_19407750	-		
rs266622383	10	55971740	-:T>C	S10_55971740	-		
rs266623367	6	55263696	-:A>G	S6_55263696	-		
rs266624752	9	44646827	-:T>C	S9_44646827	-		
rs266625042	7	7630164	-:C>G	S7_7630164	-		
rs266625260	3	58935213	-:T>A	S3_58935213	-		
rs266626043	4	8815121	-:C>T	S4_8815121	-		
rs266627344	7	60936898	-:C>G	S7_60936898	-		
rs266627457	2	46793058	-:A>T	S2_46793058	-		
rs266630402	5	68337776	-:G>A	S5_68337776	-		
rs266631626	9	51847940	-:G>C	S9_51847940	-		
rs266633223	6	47759792	-:C>G	S6_47759792	-		
rs266636974	10	18312546	-:C>A	S10_18312546	-		
rs266638833	4	64684360	-:T>C	S4_64684360	-		
rs266639540	6	3310278	-:G>A	S6_3310278	-		
rs266641751	5	3584452	-:T>C	S5_3584452	-		
rs266641777	10	3233078	-:T>G	S10_3233078	-		
rs266644279	2	7767275	-:C>A	S2_7767275	-		
rs266645133	2	47294140	-:G>A	S2_47294140	-		
rs266645260	3	63822154	-:G>T	S3_63822154	-		
rs266645694	7	62478858	-:A>G	S7_62478858	-		
rs266646474	5	66081206	-:C>A	S5_66081206	-		
rs266646728	8	59587265	-:C>T	S8_59587265	-		
rs266649021	6	60349036	-:C>G	S6_60349036	-		
rs266650477	10	56885748	-:A>G	S10_56885748	-		
rs266651103	9	48117565	-:G>A	S9_48117565	-		
rs266651905	10	11007660	-:C>T	S10_11007660	-		

rs266652659	8	2631057	-:A>G	S8_2631057	-			
rs266653760	9	52122945	-:T>C	S9_52122945	-			
rs266654398	6	60441494	-:T>G	S6_60441494	-			
rs266654583	9	461218	-:A>C	S9_461218	-			
rs266656309	4	56998548	-:G>A	S4_56998548	-			
rs266657125	4	54095533	-:A>G	S4_54095533	-			
rs266657235	2	59611033	-:A>G	S2_59611033	-			
rs266659100	9	44665579	-:G>A	S9_44665579	-			
rs266660566	1	402592	-:C>A	S1_402592	-			
rs266660842	3	64266888	-:T>G	S3_64266888	-			
rs266661302	10	47188252	-:C>G	S10_47188252	-			
rs266662775	3	58848045	-:T>G	S3_58848045	-			
rs266663206	3	63564113	-:C>T	S3_63564113	-			
rs266664848	1	20685959	-:G>C	S1_20685959	-			
rs266664991	3	50721829	-:G>C	S3_50721829	-			
rs266665597	7	62167754	-:C>G	S7_62167754	-			
rs266668458	9	1315761	-:T>C	S9_1315761	-			
rs266669377	9	13375820	-:C>G	S9_13375820	-			
rs266670768	7	2771499	-:C>T	S7_2771499	-			
rs266671433	9	15251394	-:C>T	S9_15251394	-			
rs266671462	5	70224804	-:A>C	S5_70224804	-			
rs266672109	5	10915646	-:A>T	S5_10915646	-			
rs266676417	4	55592509	-:C>T	S4_55592509	-			
rs266677433	6	53592346	-:A>C	S6_53592346	-			
rs266679388	10	7557386	-:G>T	S10_7557386	-			
rs266679941	3	64341310	-:T>C	S3_64341310	-			
rs266682288	10	7769111	-:T>C	S10_7769111	-			
rs266684470	4	63956221	-:A>G	S4_63956221	-			
rs266685005	5	69512674	-:T>G	S5_69512674	-			
rs266685289	1	71822175	-:T>C	S1_71822175	-			
rs266686343	1	16406843	-:A>G	S1_16406843	-			
rs266687202	9	25819683	-:G>C	S9_25819683	-			
rs266689547	9	42430311	-:A>G	S9_42430311	-			
rs266690673	5	68228905	-:C>G	S5_68228905	-			
rs266695065	1	13444316	-:C>T	S1_13444316	-			
rs266695982	1	45968653	-:T>C	S1_45968653	-			
rs266696290	5	11539181	-:T>C	S5_11539181	-			
rs266697573	5	69852503	-:G>A	S5_69852503	-			
rs266698038	8	61570299	-:T>C	S8_61570299	-			
rs266699093	3	10971047	-:A>G	S3_10971047	-			
rs266699247	9	52806170	-:A>T	S9_52806170	-			
rs266699350	6	47179645	-:T>A	S6_47179645	-			
rs266700946	6	42653400	-:C>T	S6_42653400	-			

rs266702123	5	17626079	-:T>C	S5_17626079	-		
rs266703100	4	57943614	-:G>A	S4_57943614	-		
rs266703954	5	11931276	-:C>G	S5_11931276	-		
rs266706292	1	51093816	-:G>T	S1_51093816	-		
rs266707002	6	49197173	-:G>A	S6_49197173	-		
rs266707762	2	75800159	-:G>T	S2_75800159	-		
rs266708133	4	9387982	-:C>T	S4_9387982	-		
rs266709060	8	10694795	-:G>A	S8_10694795	-		
rs266709817	2	76427948	-:A>G	S2_76427948	-		
rs266710222	4	61552577	-:A>T	S4_61552577	-		
rs266711536	10	57740844	-:C>T	S10_57740844	-		
rs266711725	9	56101938	-:T>C	S9_56101938	-		
rs266713225	3	9722025	-:G>A	S3_9722025	-		
rs266716301	8	60828310	-:T>G	S8_60828310	-		
rs266716365	6	48041380	-:G>A	S6_48041380	-		
rs266716440	10	59773490	-:G>A	S10_59773490	-		
rs266716458	4	8930790	-:A>G	S4_8930790	-		
rs266716959	10	53235767	-:A>G	S10_53235767	-		
rs266718068	10	1744527	-:G>C	S10_1744527	-		
rs266718832	5	69998956	-:G>A	S5_69998956	-		
rs266719487	8	57039879	-:T>A	S8_57039879	-		
rs266721767	10	10654517	-:A>G	S10_10654517	-		
rs266722041	7	64472877	-:C>T	S7_64472877	-		
rs266722844	8	2879089	-:C>G	S8_2879089	-		
rs266724175	2	70563552	-:A>T	S2_70563552	-		
rs266724960	5	3566738	-:T>G	S5_3566738	-		
rs266725542	9	54316399	-:C>G	S9_54316399	-		
rs266727054	7	1838966	-:A>T	S7_1838966	-		
rs266728158	4	6480661	-:A>G	S4_6480661	-		
rs266733053	1	74580234	-:C>A	S1_74580234	-		
rs266734431	3	63353066	-:C>T	S3_63353066	-		
rs266734962	1	59700201	-:T>C	S1_59700201	-		
rs266735134	7	62640808	-:A>T	S7_62640808	-		
rs266735712	2	58992066	-:C>T	S2_58992066	-		
rs266736622	4	65553259	-:A>G	S4_65553259	-		
rs266736939	4	59508746	-:C>G	S4_59508746	-		
rs266737321	5	3768802	-:G>C	S5_3768802	-		
rs266738124	10	670699	-:C>T	S10_670699	-		
rs266741826	8	51868231	-:G>A	S8_51868231	-		
rs266741984	8	49673676	-:C>T	S8_49673676	-		
rs266743977	3	10430104	-:C>A	S3_10430104	-		
rs266748617	5	58554352	-:T>G	S5_58554352	-		
rs266748874	10	6011948	-:G>C	S10_6011948	-		

rs266749559	5	5814004	-:C>G	S5_5814004	-		
rs266749923	5	68881899	-:A>G	S5_68881899	-		
rs266750421	6	32142574	-:A>G	S6_32142574	-		
rs266751236	9	50594866	-:T>G	S9_50594866	-		
rs266751993	5	66622383	-:G>T	S5_66622383	-		
rs266753698	2	74017412	-:C>G	S2_74017412	-		
rs266754260	7	54307379	-:C>T	S7_54307379	-		
rs266755456	1	15084897	-:T>C	S1_15084897	-		
rs266756041	7	2543093	-:G>A	S7_2543093	-		
rs266756334	10	46046605	-:T>C	S10_46046605	-		
rs266758738	2	71468022	-:G>T	S2_71468022	-		
rs266760267	5	9051425	-:G>A	S5_9051425	-		
rs266761451	8	55843085	-:A>G	S8_55843085	-		
rs266761500	4	48069338	-:T>A	S4_48069338	-		
rs266763023	5	64669495	-:G>T	S5_64669495	-		
rs266764695	1	80660369	-:T>C	S1_80660369	-		
rs266766243	3	54724099	-:C>G	S3_54724099	-		
rs266766566	3	2190998	-:T>G	S3_2190998	-		
rs266768651	5	16116728	-:C>T	S5_16116728	-		
rs266773076	1	72623579	-:T>C	S1_72623579	-		
rs266773712	3	68831857	-:A>C	S3_68831857	-		
rs266774352	6	39929198	-:T>C	S6_39929198	-		
rs266774658	3	62561559	-:T>C	S3_62561559	-		
rs266776548	3	10151012	-:A>C	S3_10151012	-		
rs266777257	6	37902123	-:A>G	S6_37902123	-		
rs266777492	5	12857271	-:C>T	S5_12857271	-		
rs266777696	6	51177337	-:T>C	S6_51177337	-		
rs266778835	9	49940632	-:G>T	S9_49940632	-		
rs266778915	9	48070479	-:A>T	S9_48070479	-		
rs266779128	6	59592835	-:C>G	S6_59592835	-		
rs266779743	8	2205697	-:C>A	S8_2205697	-		
rs266780114	3	9318294	-:A>G	S3_9318294	-		
rs266782689	2	10687296	-:A>T	S2_10687296	-		
rs266783848	8	57450092	-:A>G	S8_57450092	-		
rs266783881	2	64234684	-:A>T	S2_64234684	-		
rs266784893	7	3487314	-:C>T	S7_3487314	-		
rs266787632	6	46545495	-:T>C	S6_46545495	-		
rs266787945	10	53260155	-:G>A	S10_53260155	-		
rs266788367	5	69163820	-:T>C	S5_69163820	-		
rs266790577	10	13903186	-:G>C	S10_13903186	-		
rs266793048	3	58246494	-:T>A	S3_58246494	-		
rs266793218	6	51256111	-:C>T	S6_51256111	-		
rs266794173	8	60240792	-:G>C	S8_60240792	-		

rs266796516	9	9815344	-:G>T	S9_9815344	-		
rs266796886	1	11674191	-:T>C	S1_11674191	-		
rs266798853	6	50704210	-:A>G	S6_50704210	-		
rs266799727	8	50444392	-:A>G	S8_50444392	-		
rs266801310	4	51882076	-:A>G	S4_51882076	-		
rs266802230	6	47672893	-:G>C	S6_47672893	-		
rs266803307	1	61874941	-:G>A	S1_61874941	-		
rs266804946	7	64516206	-:C>A	S7_64516206	-		
rs266806305	3	12821290	-:G>C	S3_12821290	-		
rs266807484	9	44009459	-:G>C	S9_44009459	-		
rs266808418	1	58263019	-:G>C	S1_58263019	-		
rs266809142	10	52577265	-:T>A	S10_52577265	-		
rs266809982	2	74972257	-:C>A	S2_74972257	-		
rs266813773	8	43931182	-:A>G	S8_43931182	-		
rs266816923	1	62465759	-:A>T	S1_62465759	-		
rs266817966	10	53963219	-:C>T	S10_53963219	-		
rs266821670	9	759789	-:G>C	S9_759789	-		
rs266821985	5	8437589	-:T>C	S5_8437589	-		
rs266823550	10	54883896	-:G>A	S10_54883896	-		
rs266823751	6	3974137	-:T>C	S6_3974137	-		
rs266825158	3	8673368	-:A>G	S3_8673368	-		
rs266827652	6	54263744	-:G>T	S6_54263744	-		
rs266829542	2	49314529	-:A>G	S2_49314529	-		
rs266830083	6	49163626	-:G>A	S6_49163626	-		
rs266830876	10	8233027	-:A>G	S10_8233027	-		
rs266832940	9	3164030	-:G>C	S9_3164030	-		
rs266833271	2	76734948	-:G>A	S2_76734948	-		
rs266833542	10	47361152	-:A>G	S10_47361152	-		
rs266837389	10	18388467	-:T>C	S10_18388467	-		
rs266838136	9	56707143	-:A>G	S9_56707143	-		
rs266838866	6	19232571	-:C>T	S6_19232571	-		
rs266841705	4	68260182	-:G>C	S4_68260182	-		
rs266843091	3	62355808	-:C>G	S3_62355808	-		
rs266843104	1	15905614	-:A>G	S1_15905614	-		
rs266843185	5	6175972	-:C>G	S5_6175972	-		
rs266845897	6	57265137	-:C>T	S6_57265137	-		
rs266847519	1	67094005	-:T>A	S1_67094005	-		
rs266851838	2	63583671	-:G>T	S2_63583671	-		
rs266852946	4	51493120	-:G>A	S4_51493120	-		
rs266853402	10	7917193	-:A>G	S10_7917193	-		
rs266854128	10	21830917	-:C>G	S10_21830917	-		
rs266854753	1	66385266	-:T>C	S1_66385266	-		
rs266855255	10	60770004	-:T>C	S10_60770004	-		

rs266855534	3	54259972	-:A>G	S3_54259972	-		
rs266855828	3	7811641	-:C>T	S3_7811641	-		
rs266857851	3	51905588	-:C>T	S3_51905588	-		
rs266857901	6	50818299	-:A>G	S6_50818299	-		
rs266860102	3	11962320	-:G>C	S3_11962320	-		
rs266861689	2	72283640	-:A>G	S2_72283640	-		
rs266863756	8	57753017	-:A>G	S8_57753017	-		
rs266864685	7	8094044	-:C>T	S7_8094044	-		
rs266864950	8	55063096	-:A>T	S8_55063096	-		
rs266865523	2	55351187	-:T>C	S2_55351187	-		
rs266865674	10	10481751	-:T>A	S10_10481751	-		
rs266866094	7	2113119	-:C>A	S7_2113119	-		
rs266866735	2	62350741	-:G>C	S2_62350741	-		
rs266867094	3	54789197	-:C>T	S3_54789197	-		
rs266868778	9	42794266	-:T>C	S9_42794266	-		
rs266871610	4	10208312	-:G>C	S4_10208312	-		
rs266871997	1	58615333	-:A>G	S1_58615333	-		
rs266872335	2	59012234	-:T>C	S2_59012234	-		
rs266872441	8	5262953	-:T>C	S8_5262953	-		
rs266874124	1	55305415	-:T>C	S1_55305415	-		
rs266874287	2	4135291	-:T>C	S2_4135291	-		
rs266875122	7	2543271	-:T>C	S7_2543271	-		
rs266875995	1	72692082	-:T>A	S1_72692082	-		
rs266876219	4	57661575	-:T>G	S4_57661575	-		
rs266878020	2	72113870	-:T>C	S2_72113870	-		
rs266879106	10	46110504	-:A>G	S10_46110504	-		
rs266879313	3	10955478	-:G>A	S3_10955478	-		
rs266881575	7	6631546	-:A>G	S7_6631546	-		
rs266887512	4	64901386	-:A>G	S4_64901386	-		
rs266889965	3	51320664	-:C>G	S3_51320664	-		
rs266890856	3	7724286	-:C>A	S3_7724286	-		
rs266892204	1	2496611	-:T>G	S1_2496611	-		
rs266892242	9	46053421	-:T>A	S9_46053421	-		
rs266892373	5	21089776	-:T>G	S5_21089776	-		
rs266898278	8	2356445	-:A>G	S8_2356445	-		
rs266898408	8	57285918	-:G>T	S8_57285918	-		
rs266898484	6	3610699	-:C>A	S6_3610699	-		
rs266899247	6	53187340	-:A>T	S6_53187340	-		
rs266899353	9	49803067	-:G>T	S9_49803067	-		
rs266899838	5	5608090	-:A>T	S5_5608090	-		
rs266900712	3	20013825	-:G>T	S3_20013825	-		
rs266902035	2	76919277	-:A>C	S2_76919277	-		
rs266902319	10	59598154	-:G>A	S10_59598154	-		

rs266902818	6	45484367	-:A>G	S6_45484367	-		
rs266903751	3	71744039	-:C>T	S3_71744039	-		
rs266903991	6	14498115	-:A>T	S6_14498115	-		
rs266904798	1	20295564	-:A>G	S1_20295564	-		
rs266905524	1	71004786	-:C>G	S1_71004786	-		
rs266906054	4	63620549	-:C>G	S4_63620549	-		
rs266908593	10	5865655	-:A>C	S10_5865655	-		
rs266909525	5	12453114	-:A>G	S5_12453114	-		
rs266909573	9	55104545	-:T>C	S9_55104545	-		
rs266910354	6	46735281	-:G>C	S6_46735281	-		
rs266911122	9	54113929	-:G>A	S9_54113929	-		
rs266914278	1	68188211	-:A>G	S1_68188211	-		
rs266915954	8	55843231	-:T>A	S8_55843231	-		
rs266916595	5	36544381	-:G>A	S5_36544381	-		
rs266916989	2	76796292	-:G>C	S2_76796292	-		
rs266918724	3	12619687	-:C>T	S3_12619687	-		
rs266919981	4	65330689	-:C>T	S4_65330689	-		
rs266920724	7	63965915	-:A>C	S7_63965915	-		
rs266921979	4	61204129	-:T>C	S4_61204129	-		
rs266922964	9	21318081	-:G>A	S9_21318081	-		
rs266923093	7	2774881	-:T>A	S7_2774881	-		
rs266923840	2	8593095	-:G>C	S2_8593095	-		
rs266924405	6	49446667	-:G>C	S6_49446667	-		
rs266924975	4	65150530	-:A>G	S4_65150530	-		
rs266925630	1	51015562	-:T>C	S1_51015562	-		
rs266925906	7	61946475	-:G>C	S7_61946475	-		
rs266926657	4	62832956	-:G>A	S4_62832956	-		
rs266927082	1	68096207	-:C>A	S1_68096207	-		
rs266927265	2	768663	-:A>G	S2_768663	-		
rs266929839	2	8383750	-:C>T	S2_8383750	-		
rs266930656	1	12137838	-:C>T	S1_12137838	-		
rs266931005	8	7159623	-:T>C	S8_7159623	-		
rs266932317	4	49525828	-:C>T	S4_49525828	-		
rs266932825	8	7012709	-:C>T	S8_7012709	-		
rs266933595	3	48913745	-:G>A	S3_48913745	-		
rs266934329	7	254017	-:A>G	S7_254017	-		
rs266934625	3	16632442	-:A>C	S3_16632442	-		
rs266934826	9	1812472	-:G>A	S9_1812472	-		
rs266936242	1	66996364	-:C>T	S1_66996364	-		
rs266936741	5	37911590	-:T>G	S5_37911590	-		
rs266936826	10	21661770	-:A>G	S10_21661770	-		
rs266936918	5	21261244	-:G>A	S5_21261244	-		
rs266937078	10	54918679	-:G>C	S10_54918679	-		

rs266939360	2	10801330	-:A>G	S2_10801330	-			
rs266939813	3	58682533	-:A>G	S3_58682533	-			
rs266947807	8	1913441	-:C>T	S8_1913441	-			
rs266948058	1	1954298	-:T>A	S1_1954298	-			
rs266948788	2	24074931	-:G>C	S2_24074931	-			
rs266949169	7	64967149	-:T>C	S7_64967149	-			
rs266949898	10	50362913	-:C>T	S10_50362913	-			
rs266950036	10	20044781	-:A>T	S10_20044781	-			
rs266950184	9	55701949	-:C>T	S9_55701949	-			
rs266952440	2	61543650	-:G>A	S2_61543650	-			
rs266952761	8	62078794	-:G>C	S8_62078794	-			
rs266955885	5	21004410	-:G>A	S5_21004410	-			
rs266956314	8	57569203	-:G>A	S8_57569203	-			
rs266957433	6	17558400	-:G>A	S6_17558400	-			
rs266957588	10	6052501	-:C>T	S10_6052501	-			
rs266958688	3	63730177	-:T>G	S3_63730177	-			
rs266959322	7	53155596	-:G>A	S7_53155596	-			
rs266962862	5	59444708	-:G>C	S5_59444708	-			
rs266962984	1	68817204	-:C>T	S1_68817204	-			
rs266964695	7	8583649	-:G>A	S7_8583649	-			
rs266964707	9	5067309	-:G>A	S9_5067309	-			
rs266964770	5	68658674	-:G>A	S5_68658674	-			
rs266964910	3	57770470	-:C>T	S3_57770470	-			
rs266966357	8	902973	-:C>T	S8_902973	-			
rs266967018	8	49663979	-:A>G	S8_49663979	-			
rs266967471	10	7855143	-:C>T	S10_7855143	-			
rs266968676	8	62470927	-:T>C	S8_62470927	-			
rs266969055	7	65354901	-:G>C	S7_65354901	-			
rs266969539	10	52969580	-:A>C	S10_52969580	-			
rs266969616	2	77184288	-:G>T	S2_77184288	-			
rs266971532	6	50583878	-:G>C	S6_50583878	-			
rs266973810	9	4867656	-:T>C	S9_4867656	-			
rs266974646	8	6114303	-:T>C	S8_6114303	-			
rs266974826	10	8753539	-:C>G	S10_8753539	-			
rs266975535	5	57995949	-:C>G	S5_57995949	-			
rs266976147	4	9599898	-:G>A	S4_9599898	-			
rs266976837	10	52527172	-:G>A	S10_52527172	-			
rs266979437	8	9195558	-:T>G	S8_9195558	-			
rs266981326	6	50647247	-:A>G	S6_50647247	-			
rs266983270	3	59421327	-:G>C	S3_59421327	-			
rs266984010	2	62422104	-:G>C	S2_62422104	-			
rs266986344	10	1493293	-:G>A	S10_1493293	-			
rs266986423	8	3195426	-:C>G	S8_3195426	-			

rs266986581	1	7640553	-:A>C	S1_7640553	-		
rs266987061	4	61402064	-:A>G	S4_61402064	-		
rs266987256	8	41501132	-:A>G	S8_41501132	-		
rs266988081	1	15724248	-:A>T	S1_15724248	-		
rs266988176	7	42752212	-:T>C	S7_42752212	-		
rs266988325	9	54317411	-:C>G	S9_54317411	-		
rs266992131	9	6694393	-:T>C	S9_6694393	-		
rs266993205	7	57819725	-:G>A	S7_57819725	-		
rs266993562	3	7365183	-:C>T	S3_7365183	-		
rs266994276	8	9299620	-:G>A	S8_9299620	-		
rs266994321	10	19641192	-:C>T	S10_19641192	-		
rs266994372	2	72882731	-:G>T	S2_72882731	-		
rs266995002	4	3755712	-:C>G	S4_3755712	-		
rs266997120	5	60791687	-:A>G	S5_60791687	-		
rs266997847	2	72138198	-:G>T	S2_72138198	-		
rs266997898	4	1237723	-:T>C	S4_1237723	-		
rs266998054	5	68299624	-:A>G	S5_68299624	-		
rs266998459	3	70457916	-:A>G	S3_70457916	-		
rs266998844	3	62321042	-:C>A	S3_62321042	-		
rs267000083	1	3558947	-:C>T	S1_3558947	-		
rs267000181	7	61070995	-:A>C	S7_61070995	-		
rs267001666	2	70670834	-:A>C	S2_70670834	-		
rs267003405	6	7765103	-:C>T	S6_7765103	-		
rs267003578	6	47443837	-:A>T	S6_47443837	-		
rs267006164	2	583083	-:A>G	S2_583083	-		
rs267006772	8	3347441	-:T>A	S8_3347441	-		
rs267007831	8	504185	-:G>C	S8_504185	-		
rs267007998	5	19688110	-:A>T	S5_19688110	-		
rs267008250	3	8412256	-:T>C	S3_8412256	-		
rs267009331	9	50907272	-:T>G	S9_50907272	-		
rs267010143	5	69997060	-:T>C	S5_69997060	-		
rs267010214	10	58947252	-:C>T	S10_58947252	-		
rs267011331	10	57870372	-:G>T	S10_57870372	-		
rs267011397	3	57959805	-:G>A	S3_57959805	-		
rs267011581	6	51734742	-:T>G	S6_51734742	-		
rs267014693	1	64013631	-:A>T	S1_64013631	-		
rs267014756	9	51976526	-:G>T	S9_51976526	-		
rs267017854	7	15404555	-:C>G	S7_15404555	-		
rs267019346	3	10338829	-:A>G	S3_10338829	-		
rs267019771	7	41050515	-:C>T	S7_41050515	-		
rs267021392	1	78619135	-:G>A	S1_78619135	-		
rs267023982	8	1240725	-:C>T	S8_1240725	-		
rs267026757	4	52988537	-:A>G	S4_52988537	-		

rs267027482	9	1676038	-:C>T	S9_1676038	-		
rs267027593	10	6938695	-:C>T	S10_6938695	-		
rs267028610	5	1315969	-:G>A	S5_1315969	-		
rs267029433	10	3316533	-:A>G	S10_3316533	-		
rs267029727	1	79657462	-:A>G	S1_79657462	-		
rs267030337	5	8222692	-:C>T	S5_8222692	-		
rs267033942	6	60532241	-:G>A	S6_60532241	-		
rs267034984	9	53562099	-:T>C	S9_53562099	-		
rs267035560	10	21052619	-:T>A	S10_21052619	-		
rs267036283	2	55520157	-:C>T	S2_55520157	-		
rs267037621	2	17641207	-:T>A	S2_17641207	-		
rs267037627	8	58756290	-:G>C	S8_58756290	-		
rs267037768	5	55985835	-:G>C	S5_55985835	-		
rs267038893	8	57539625	-:T>G	S8_57539625	-		
rs267041720	6	28158855	-:A>T	S6_28158855	-		
rs267041810	8	4920840	-:C>A	S8_4920840	-		
rs267042690	3	54139465	-:A>C	S3_54139465	-		
rs267044391	6	39938146	-:A>G	S6_39938146	-		
rs267044805	4	55031314	-:A>G	S4_55031314	-		
rs267045429	10	46073766	-:T>G	S10_46073766	-		
rs267045586	6	60847862	-:C>G	S6_60847862	-		
rs267045989	1	66235048	-:T>C	S1_66235048	-		
rs267046940	2	59264062	-:G>C	S2_59264062	-		
rs267047332	2	77018405	-:A>G	S2_77018405	-		
rs267049693	7	13913109	-:T>A	S7_13913109	-		
rs267051311	2	59577465	-:A>C	S2_59577465	-		
rs267052223	7	60850373	-:A>G	S7_60850373	-		
rs267052427	3	9939593	-:T>C	S3_9939593	-		
rs267054924	4	62990016	-:A>G	S4_62990016	-		
rs267055156	6	58508740	-:T>G	S6_58508740	-		
rs267055678	1	65144480	-:G>T	S1_65144480	-		
rs267056035	1	2184106	-:A>C	S1_2184106	-		
rs267056699	5	67736587	-:G>A	S5_67736587	-		
rs267056730	9	47156087	-:T>C	S9_47156087	-		
rs267056901	10	52878053	-:C>T	S10_52878053	-		
rs267058525	8	49285354	-:C>T	S8_49285354	-		
rs267058635	3	64858480	-:C>G	S3_64858480	-		
rs267058662	4	54682676	-:A>G	S4_54682676	-		
rs267059473	1	19118158	-:C>T	S1_19118158	-		
rs267060492	8	60130799	-:T>C	S8_60130799	-		
rs267060573	1	69080916	-:C>T	S1_69080916	-		
rs267066612	9	485150	-:T>A	S9_485150	-		
rs267066939	8	5379958	-:T>G	S8_5379958	-		

rs267069053	1	71823019	-:G>A	S1_71823019	-		
rs267073882	8	61837648	-:A>G	S8_61837648	-		
rs267075309	7	56629979	-:C>T	S7_56629979	-		
rs267075797	4	59807941	-:G>A	S4_59807941	-		
rs267076727	2	59734197	-:G>C	S2_59734197	-		
rs267077086	8	47530952	-:A>G	S8_47530952	-		
rs267081641	2	71496345	-:T>C	S2_71496345	-		
rs267081855	2	23857220	-:T>A	S2_23857220	-		
rs267082632	6	53456518	-:C>T	S6_53456518	-		
rs267083124	5	8039872	-:G>T	S5_8039872	-		
rs267084046	9	47264711	-:T>G	S9_47264711	-		
rs267084096	3	8122262	-:G>C	S3_8122262	-		
rs267084375	1	59433026	-:G>T	S1_59433026	-		
rs267086529	8	53132494	-:G>T	S8_53132494	-		
rs267087465	4	4230338	-:T>G	S4_4230338	-		
rs267088585	5	63404087	-:T>C	S5_63404087	-		
rs267095305	3	73912511	-:G>A	S3_73912511	-		
rs267099265	10	10690326	-:C>G	S10_10690326	-		
rs267100942	6	9536906	-:G>C	S6_9536906	-		
rs267101774	1	9142676	-:T>C	S1_9142676	-		
rs267102233	7	2065778	-:C>T	S7_2065778	-		
rs267103304	1	30172622	-:C>G	S1_30172622	-		
rs267104221	4	58438072	-:G>A	S4_58438072	-		
rs267105283	2	10289495	-:T>A	S2_10289495	-		
rs267105757	7	7619279	-:A>G	S7_7619279	-		
rs267105991	8	54098382	-:C>T	S8_54098382	-		
rs267106337	4	6610538	-:A>G	S4_6610538	-		
rs267106645	2	72138932	-:G>C	S2_72138932	-		
rs267107037	10	58057862	-:T>C	S10_58057862	-		
rs267107315	1	4119014	-:A>G	S1_4119014	-		
rs267110078	6	4707249	-:A>G	S6_4707249	-		
rs267110392	1	7960014	-:G>A	S1_7960014	-		
rs267111217	7	64061268	-:A>G	S7_64061268	-		
rs267111598	2	11090424	-:C>A	S2_11090424	-		
rs267113621	9	54733896	-:A>G	S9_54733896	-		
rs267115414	5	21004695	-:C>G	S5_21004695	-		
rs267117954	1	53106539	-:T>C	S1_53106539	-		
rs267118019	2	65894368	-:G>A	S2_65894368	-		
rs267118911	2	65660828	-:C>A	S2_65660828	-		
rs267119711	2	75155295	-:A>C	S2_75155295	-		
rs267120696	8	7872297	-:C>T	S8_7872297	-		
rs267122902	6	54193169	-:A>G	S6_54193169	-		
rs267126181	2	9277982	-:C>T	S2_9277982	-		

rs267128728	7	57380586	-:G>T	S7_57380586	-		
rs267129883	7	7610042	-:G>C	S7_7610042	-		
rs267132512	5	3566148	-:T>C	S5_3566148	-		
rs267133976	5	70651344	-:A>T	S5_70651344	-		
rs267135547	3	1095824	-:T>C	S3_1095824	-		
rs267139240	4	2891258	-:C>G	S4_2891258	-		
rs267141304	8	56098244	-:T>A	S8_56098244	-		
rs267143135	2	7347374	-:A>G	S2_7347374	-		
rs267145480	5	70870208	-:T>G	S5_70870208	-		
rs267145864	4	2871791	-:G>C	S4_2871791	-		
rs267147517	2	59726888	-:A>T	S2_59726888	-		
rs267147831	10	56540746	-:G>A	S10_56540746	-		
rs267148440	3	71732278	-:C>T	S3_71732278	-		
rs267148744	7	4684475	-:A>G	S7_4684475	-		
rs267148876	3	10722262	-:C>G	S3_10722262	-		
rs267150904	7	8120290	-:G>T	S7_8120290	-		
rs267151359	9	14472373	-:T>C	S9_14472373	-		
rs267151383	10	25842062	-:A>G	S10_25842062	-		
rs267152406	5	63531462	-:C>T	S5_63531462	-		
rs267157978	6	29590198	-:A>G	S6_29590198	-		
rs267160141	10	9282679	-:G>C	S10_9282679	-		
rs267160214	7	54506420	-:C>A	S7_54506420	-		
rs267161325	10	59708582	-:T>G	S10_59708582	-		
rs267162864	10	59476916	-:C>T	S10_59476916	-		
rs267163530	2	73488546	-:A>C	S2_73488546	-		
rs267164054	10	24402884	-:A>G	S10_24402884	-		
rs267164150	2	65540021	-:C>A	S2_65540021	-		
rs267164387	5	19798041	-:C>T	S5_19798041	-		
rs267165408	1	64361145	-:T>C	S1_64361145	-		
rs267166231	9	58176710	-:C>T	S9_58176710	-		
rs267166292	7	61860237	-:G>C	S7_61860237	-		
rs267166370	5	71600716	-:G>C	S5_71600716	-		
rs267167261	5	63334951	-:G>A	S5_63334951	-		
rs267167278	1	62758221	-:G>T	S1_62758221	-		
rs267168725	1	53456591	-:C>G	S1_53456591	-		
rs267170743	6	48555416	-:C>T	S6_48555416	-		
rs267171188	7	61598346	-:T>C	S7_61598346	-		
rs267172725	2	17293213	-:T>G	S2_17293213	-		
rs267173372	8	5987676	-:A>C	S8_5987676	-		
rs267174059	1	3156917	-:G>A	S1_3156917	-		
rs267174331	1	18954741	-:C>T	S1_18954741	-		
rs267175466	4	61204000	-:T>C	S4_61204000	-		
rs267175573	9	33820065	-:A>G	S9_33820065	-		

rs267175583	10	53394524	-:A>C	S10_53394524	-		
rs267175822	3	69879206	-:T>C	S3_69879206	-		
rs267178623	7	10065673	-:A>T	S7_10065673	-		
rs267179239	1	60885424	-:C>T	S1_60885424	-		
rs267179356	2	66465051	-:G>C	S2_66465051	-		
rs267180796	6	42183773	-:A>G	S6_42183773	-		
rs267181252	9	9620062	-:C>G	S9_9620062	-		
rs267181539	9	51779030	-:T>C	S9_51779030	-		
rs267183196	1	9038904	-:C>T	S1_9038904	-		
rs267183574	3	5137603	-:G>A	S3_5137603	-		
rs267186683	10	53287916	-:A>G	S10_53287916	-		
rs267186691	5	8063027	-:A>G	S5_8063027	-		
rs267188595	10	3291260	-:C>G	S10_3291260	-		
rs267190534	2	10144841	-:C>G	S2_10144841	-		
rs267190829	7	15926392	-:T>C	S7_15926392	-		
rs267191558	1	78275181	-:C>T	S1_78275181	-		
rs267193996	4	54794920	-:G>A	S4_54794920	-		
rs267195038	7	2509153	-:A>G	S7_2509153	-		
rs267195719	9	14137987	-:A>G	S9_14137987	-		
rs267196139	5	5814548	-:C>A	S5_5814548	-		
rs267196206	2	70202085	-:T>C	S2_70202085	-		
rs267198315	3	12301649	-:A>G	S3_12301649	-		
rs267198880	3	3256114	-:G>A	S3_3256114	-		
rs267199546	8	56595318	-:G>A	S8_56595318	-		
rs267201471	6	43672535	-:G>C	S6_43672535	-		
rs267203224	3	9897400	-:G>T	S3_9897400	-		
rs267203676	3	11649027	-:T>G	S3_11649027	-		
rs267203857	9	24253940	-:A>G	S9_24253940	-		
rs267204178	2	59822114	-:A>G	S2_59822114	-		
rs267204688	5	3214952	-:A>T	S5_3214952	-		
rs267205527	6	50791904	-:T>C	S6_50791904	-		
rs267207781	4	63457188	-:C>G	S4_63457188	-		
rs267209381	7	8434105	-:C>G	S7_8434105	-		
rs267209453	9	4110620	-:C>T	S9_4110620	-		
rs267209701	2	9809961	-:A>G	S2_9809961	-		
rs267211394	9	55826560	-:C>A	S9_55826560	-		
rs267212054	10	49639804	-:G>C	S10_49639804	-		
rs267213189	2	10517472	-:A>T	S2_10517472	-		
rs267214112	5	62196166	-:A>G	S5_62196166	-		
rs267216513	1	57883020	-:C>A	S1_57883020	-		
rs267220463	9	1812682	-:G>A	S9_1812682	-		
rs267220546	1	55219762	-:G>A	S1_55219762	-		
rs267221886	5	67567058	-:C>T	S5_67567058	-		

rs267223115	7	60598717	-:T>C	S7_60598717	-		
rs267224057	6	3201106	-:A>G	S6_3201106	-		
rs267230042	9	640765	-:G>C	S9_640765	-		
rs267230599	8	4278542	-:A>G	S8_4278542	-		
rs267231036	4	56534949	-:A>C	S4_56534949	-		
rs267231717	3	5202296	-:A>G	S3_5202296	-		
rs267231889	5	50600981	-:A>G	S5_50600981	-		
rs267232914	2	70106486	-:C>T	S2_70106486	-		
rs3379576414	4	66691004	-:C>T	S4_66691004	-		
rs3379594892	10	60691171	-:C>A	S10_60691171	-		
rs3379672383	1	2883982	-:T>G	S1_2883982	-		
rs3379703808	1	26239948	-:G>C	S1_26239948	-		
rs3379744930	1	66828431	-:G>A	S1_66828431	-		
rs3379748634	1	61255078	-:G>A	S1_61255078	-		
rs3379759614	1	65756223	-:T>G	S1_65756223	-		
rs3379759649	1	66504156	-:G>A	S1_66504156	-		
rs3379762396	1	66565171	-:G>A	S1_66565171	-		
rs3379775208	1	74280158	-:T>G	S1_74280158	-		
rs3379841683	2	61272138	-:C>T	S2_61272138	-		
rs3379856713	2	52558089	-:G>A	S2_52558089	-		
rs3379857029	2	68179736	-:T>G	S2_68179736	-		
rs3379861054	2	55268019	-:A>G	S2_55268019	-		
rs3379863982	2	61130174	-:C>T	S2_61130174	-		
rs3379867095	2	60921531	-:T>A	S2_60921531	-		
rs3379878025	2	70398364	-:A>T	S2_70398364	-		
rs3379878975	2	68414902	-:T>A	S2_68414902	-		
rs3379899066	3	3702083	-:G>T	S3_3702083	-		
rs3379908420	3	5537819	-:C>G	S3_5537819	-		
rs3379922642	3	4555653	-:C>A	S3_4555653	-		
rs3379930823	3	15675914	-:T>G	S3_15675914	-		
rs3379939533	3	57291336	-:G>C	S3_57291336	-		
rs3379939586	3	57545093	-:C>G	S3_57545093	-		
rs3379941950	3	58950239	-:A>G	S3_58950239	-		
rs3379945007	3	30249826	-:T>C	S3_30249826	-		
rs3379962938	3	52629214	-:A>G	S3_52629214	-		
rs3379963406	3	54150222	-:C>T	S3_54150222	-		
rs3379969571	3	52314137	-:G>A	S3_52314137	-		
rs3379971557	3	56294726	-:C>G	S3_56294726	-		
rs3379977485	3	57544976	-:G>T	S3_57544976	-		
rs3379979529	3	58266487	-:A>G	S3_58266487	-		
rs3379981048	3	60582529	-:A>C	S3_60582529	-		
rs3379982935	3	64843167	-:A>G	S3_64843167	-		
rs3379985960	3	60718995	-:C>T	S3_60718995	-		

rs3379986375	3	61918842	-:A>G	S3_61918842	-		
rs3379998124	3	69833512	-:A>G	S3_69833512	-		
rs3380005430	3	72465954	-:T>C	S3_72465954	-		
rs3380015870	5	3534549	-:G>C	S5_3534549	-		
rs3380023835	5	4616776	-:C>G	S5_4616776	-		
rs3380035512	5	7295750	-:T>C	S5_7295750	-		
rs3380043993	5	33883219	-:C>T	S5_33883219	-		
rs3380047312	5	26922359	-:G>A	S5_26922359	-		
rs3380048455	5	27577195	-:C>T	S5_27577195	-		
rs3380053692	5	33882609	-:G>A	S5_33882609	-		
rs3380057420	5	33942089	-:C>T	S5_33942089	-		
rs3380065040	5	34231061	-:T>G	S5_34231061	-		
rs3380089157	5	70610574	-:C>G	S5_70610574	-		
rs3380105495	4	50587650	-:A>C	S4_50587650	-		
rs3380122782	4	13431645	-:G>A	S4_13431645	-		
rs3380129414	4	18137154	-:C>T	S4_18137154	-		
rs3380130792	4	13905476	-:C>T	S4_13905476	-		
rs3380141422	4	57041480	-:C>G	S4_57041480	-		
rs3380149389	7	2554349	-:C>T	S7_2554349	-		
rs3380156636	4	54826449	-:G>A	S4_54826449	-		
rs3380162904	4	52726600	-:C>G	S4_52726600	-		
rs3380166772	4	52472706	-:C>T	S4_52472706	-		
rs3380175251	4	61449809	-:C>G	S4_61449809	-		
rs3380188441	4	66419457	-:C>G	S4_66419457	-		
rs3380191402	4	59526825	-:C>A	S4_59526825	-		
rs3380223482	7	58155181	-:T>C	S7_58155181	-		
rs3380248840	8	1070295	-:C>A	S8_1070295	-		
rs3380254104	7	59033882	-:A>G	S7_59033882	-		
rs3380257868	7	53994045	-:C>G	S7_53994045	-		
rs3380258701	8	1238786	-:C>A	S8_1238786	-		
rs3380265067	7	64163902	-:T>C	S7_64163902	-		
rs3380271485	8	1234813	-:T>G	S8_1234813	-		
rs3380281001	8	3429289	-:C>T	S8_3429289	-		
rs3380293374	8	3429420	-:T>C	S8_3429420	-		
rs3380303792	8	56474994	-:G>T	S8_56474994	-		
rs3380344209	8	56639142	-:C>T	S8_56639142	-		
rs3380363187	6	1912562	-:G>C	S6_1912562	-		
rs3380366316	6	48896330	-:G>T	S6_48896330	-		
rs3380367026	6	42793946	-:A>T	S6_42793946	-		
rs3380372724	6	43322397	-:C>A	S6_43322397	-		
rs3380382837	6	43657028	-:G>C	S6_43657028	-		
rs3380387616	6	45203134	-:A>T	S6_45203134	-		
rs3380389621	6	60104480	-:A>C	S6_60104480	-		

rs3380389633	6	60125869	-:G>A	S6_60125869	-		
rs3380391190	10	23206881	-:C>G	S10_23206881	-		
rs3380392650	6	46019257	-:C>T	S6_46019257	-		
rs3380396735	6	42206494	-:G>C	S6_42206494	-		
rs3380424235	6	56335798	-:C>T	S6_56335798	-		
rs3380427342	10	8964377	-:T>G	S10_8964377	-		
rs3380430894	6	61073629	-:G>T	S6_61073629	-		
rs3380431089	10	4328072	-:C>T	S10_4328072	-		
rs3380436480	9	4313950	-:T>C	S9_4313950	-		
rs3380442845	10	4602556	-:A>G	S10_4602556	-		
rs3380465566	10	8265302	-:A>G	S10_8265302	-		
rs3380467318	10	59293078	-:T>C	S10_59293078	-		
rs3380482443	9	3163806	-:A>G	S9_3163806	-		
rs3380486010	9	54573745	-:A>G	S9_54573745	-		
rs3380521636	9	41088470	-:G>A	S9_41088470	-		
rs3380534562	9	1863325	-:T>G	S9_1863325	-		
rs3380552811	9	47120193	-:G>A	S9_47120193	-		
rs3380557663	9	52364649	-:G>A	S9_52364649	-		
rs3380557827	9	57463227	-:C>T	S9_57463227	-		
rs3380566669	9	53383648	-:G>A	S9_53383648	-		
rs3380567217	9	53384220	-:G>T	S9_53384220	-		
rs3380573961	9	59090384	-:C>T	S9_59090384	-		
rs3380577859	7	62716960	-:A>C	S7_62716960	-		
rs3380582471	9	56217107	-:T>G	S9_56217107	-		
rs5415687646	1	35051215	-:T>C	S1_35051215	-		
rs5415726976	1	35278816	-:A>G	S1_35278816	-		
rs5416381887	1	51357274	-:G>A	S1_51357274	-		
rs5416431764	1	51320909	-:G>C	S1_51320909	-		
rs5417434874	1	71516550	-:T>C	S1_71516550	-		
rs5418435084	2	11431335	-:C>A	S2_11431335	-		
rs5418825858	2	17975913	-:G>A	S2_17975913	-		
rs5421263358	2	57444453	-:G>A	S2_57444453	-		
rs5421837114	2	62726243	-:C>T	S2_62726243	-		
rs5422114429	2	72569287	-:G>A	S2_72569287	-		
rs5422152906	2	73366315	-:G>C	S2_73366315	-		
rs5422157866	2	72180200	-:C>T	S2_72180200	-		
rs5422208875	2	72217463	-:G>C	S2_72217463	-		
rs5422217442	2	75649815	-:T>C	S2_75649815	-		
rs5422415243	3	3441687	-:C>T	S3_3441687	-		
rs5425399898	3	52047978	-:G>A	S3_52047978	-		
rs5425852866	3	69988007	-:G>C	S3_69988007	-		
rs5426199547	3	71936671	-:G>A	S3_71936671	-		
rs5426378355	4	930255	-:C>G	S4_930255	-		

rs5426707460	4	6703433	-:G>C	S4_6703433	-		
rs5429436891	4	55659061	-:A>G	S4_55659061	-		
rs5429655168	4	56251228	-:A>G	S4_56251228	-		
rs5430430946	5	3865160	-:G>C	S5_3865160	-		
rs5432188525	5	27269045	-:G>A	S5_27269045	-		
rs5432611660	5	33946205	-:A>C	S5_33946205	-		
rs5432692569	5	33883107	-:G>T	S5_33883107	-		
rs5433703959	5	52009469	-:C>G	S5_52009469	-		
rs5434912329	5	71686329	-:C>G	S5_71686329	-		
rs5434944925	5	68691842	-:T>G	S5_68691842	-		
rs5435311199	6	6738864	-:C>A	S6_6738864	-		
rs5435344089	6	3463592	-:T>C	S6_3463592	-		
rs5437181743	6	38113163	-:G>T	S6_38113163	-		
rs5437447101	6	43305355	-:G>C	S6_43305355	-		
rs5437690646	6	51538260	-:C>G	S6_51538260	-		
rs5437742009	6	49170717	-:C>T	S6_49170717	-		
rs5437864440	7	3451743	-:T>G	S7_3451743	-		
rs5438105884	6	54462665	-:G>A	S6_54462665	-		
rs5438266186	6	58602944	-:T>C	S6_58602944	-		
rs5441420958	7	57847909	-:C>G	S7_57847909	-		
rs5441711654	7	64745742	-:C>A	S7_64745742	-		
rs5442858869	8	15405996	-:A>G	S8_15405996	-		
rs5445061905	8	62193730	-:C>A	S8_62193730	-		
rs5445476566	8	57974123	-:T>A	S8_57974123	-		
rs5445624760	8	54938492	-:G>A	S8_54938492	-		
rs5446139869	9	7350298	-:C>T	S9_7350298	-		
rs5447446839	9	47624024	-:T>C	S9_47624024	-		
rs5448480070	9	51718677	-:C>T	S9_51718677	-		
rs5448506912	9	45012279	-:G>A	S9_45012279	-		
rs5448981119	10	11113367	-:A>C	S10_11113367	-		
rs5449311927	10	9044465	-:A>G	S10_9044465	-		
rs5449723070	10	6620386	-:G>C	S10_6620386	-		
rs5451400681	10	56387382	-:C>G	S10_56387382	-		
rs5452523365	10	56873383	-:G>A	S10_56873383	-		
rs5452829412	10	57873298	-:C>T	S10_57873298	-		
rs5452917392	5	33895729	-:C>T	S5_33895729	-		
rs5980786897	4	68044860	-:G>T	S4_68044860	-		EVA_fast_track
rs5980786908	9	40780000	-:G>T	S9_40780000	-		EVA_fast_track
rs5980786920	3	62167961	-:G>T	S3_62167961	-		EVA_fast_track
rs5980786950	7	64137002	-:T>G	S7_64137002	-		EVA_fast_track
rs5980786971	7	9684456	-:G>T	S7_9684456	-		EVA_fast_track
rs5980786972	9	56636586	-:G>A	S9_56636586	-		EVA_fast_track
rs5980786974	4	3667965	-:C>A	S4_3667965	-		EVA_fast_track

rs5980786975	5	71386421	-:C>A	S5_71386421	-			EVA_fast_track
rs5980786978	9	54983384	-:T>A	S9_54983384	-			EVA_fast_track
rs5980786979	3	72569827	-:G>T	S3_72569827	-			EVA_fast_track
rs5980786981	8	56115321	-:A>G	S8_56115321	-			EVA_fast_track
rs5980786982	7	43227525	-:A>G	S7_43227525	-			EVA_fast_track
rs795910454	3	72511821	-:T>C	S3_72511821	-			
rs795911092	9	53139314	-:A>G	S9_53139314	-			
rs795916885	5	6092084	-:C>T	S5_6092084	-			
rs795917608	9	50020188	-:A>C	S9_50020188	-			
rs795918373	3	73278322	-:A>G	S3_73278322	-			
rs795918834	3	72444136	-:C>T	S3_72444136	-			
rs795919512	9	46820433	-:G>C	S9_46820433	-			
rs795919797	2	67454195	-:G>T	S2_67454195	-			
rs795922044	2	62014331	-:G>T	S2_62014331	-			
rs795922256	1	11121964	-:C>G	S1_11121964	-			
rs795923362	4	50107217	-:G>T	S4_50107217	-			
rs795924875	7	62056620	-:T>G	S7_62056620	-			
rs795926158	8	56724683	-:G>T	S8_56724683	-			
rs795937348	4	62227618	-:G>C	S4_62227618	-			
rs870219962	4	12354641	-:A>G	S4_12354641	-			
rs870248923	10	4109725	-:C>T	S10_4109725	-			
rs870251731	2	62112649	-:A>G	S2_62112649	-			
rs870255777	6	2454378	-:C>T	S6_2454378	-			
rs870259849	1	53059558	-:T>C	S1_53059558	-			
rs870260901	9	10594827	-:C>G	S9_10594827	-			
rs870271175	4	55765917	-:C>A	S4_55765917	-			
rs870283927	4	51805812	-:T>C	S4_51805812	-			
rs870291207	3	57352586	-:G>T	S3_57352586	-			
rs870291269	9	54778366	-:A>C	S9_54778366	-			
rs870297505	1	60838090	-:T>C	S1_60838090	-			
rs870305003	9	50164880	-:A>G	S9_50164880	-			
rs870313371	1	17716760	-:G>C	S1_17716760	-			
rs870313658	5	68205140	-:G>C	S5_68205140	-			
rs870314141	6	4677150	-:C>T	S6_4677150	-			
rs870314313	5	8838314	-:G>C	S5_8838314	-			
rs870326034	3	66330611	-:G>A	S3_66330611	-			
rs870352731	1	25290010	-:T>A	S1_25290010	-			
rs870356249	4	12071073	-:G>T	S4_12071073	-			
rs870356647	4	60183779	-:T>C	S4_60183779	-			
rs870357523	6	48402568	-:C>T	S6_48402568	-			
rs870358954	1	67257998	-:C>T	S1_67257998	-			
rs870365534	1	70890586	-:C>T	S1_70890586	-			
rs870369698	2	71429368	-:G>T	S2_71429368	-			

rs870394029	10	4030750	-:C>T	S10_4030750	-		
rs870401680	9	47257153	-:A>T	S9_47257153	-		
rs870404533	3	51042434	-:G>A	S3_51042434	-		
rs870428443	3	116624	-:G>C	S3_116624	-		
rs870429006	8	56745629	-:C>A	S8_56745629	-		
rs870442998	2	52127520	-:G>A	S2_52127520	-		
rs870449537	4	43486097	-:C>T	S4_43486097	-		
rs870456344	3	72455728	-:T>G	S3_72455728	-		
rs870459135	5	9025495	-:A>G	S5_9025495	-		
rs870471971	3	57238323	-:A>G	S3_57238323	-		
rs870485391	10	56998184	-:T>A	S10_56998184	-		
rs870488426	6	47937092	-:A>C	S6_47937092	-		
rs870493612	2	20171454	-:C>G	S2_20171454	-		
rs870498312	3	11276698	-:A>G	S3_11276698	-		
rs870502230	4	52474061	-:G>A	S4_52474061	-		
rs870506679	3	8974634	-:A>T	S3_8974634	-		
rs870514501	4	54762420	-:A>G	S4_54762420	-		
rs870515360	2	19788757	-:C>T	S2_19788757	-		
rs870526058	1	1019896	-:C>G	S1_1019896	-		
rs870528734	8	58556292	-:C>T	S8_58556292	-		
rs870540843	3	4733040	-:G>T	S3_4733040	-		
rs870564772	1	32486630	-:C>T	S1_32486630	-		
rs870582173	9	45599314	-:T>C	S9_45599314	-		
rs870594460	8	59681050	-:C>T	S8_59681050	-		
rs870604387	9	56499865	-:C>A	S9_56499865	-		
rs870606415	3	72441614	-:G>A	S3_72441614	-		
rs870606797	2	6518297	-:G>T	S2_6518297	-		
rs870628711	4	3791907	-:G>A	S4_3791907	-		
rs870636270	4	56566643	-:T>G	S4_56566643	-		
rs870636560	6	51098542	-:T>G	S6_51098542	-		
rs870659558	4	58482630	-:A>G	S4_58482630	-		
rs870683851	6	47793719	-:C>G	S6_47793719	-		
rs870694790	2	60406949	-:C>A	S2_60406949	-		
rs870708343	7	62915731	-:G>C	S7_62915731	-		
rs870720976	1	28959235	-:G>A	S1_28959235	-		
rs870722029	7	7921806	-:C>T	S7_7921806	-		
rs870726317	4	60975078	-:C>T	S4_60975078	-		
rs870750379	5	62015333	-:G>A	S5_62015333	-		
rs870752192	6	47419484	-:G>A	S6_47419484	-		
rs870753673	6	61250660	-:T>C	S6_61250660	-		
rs870754616	7	62167567	-:A>G	S7_62167567	-		
rs870761679	3	70245187	-:C>T	S3_70245187	-		
rs870767686	6	49460970	-:G>A	S6_49460970	-		

rs870795325	7	57110137	-:G>A	S7_57110137	-		
rs870796876	5	62738479	-:A>G	S5_62738479	-		
rs870798740	6	1839684	-:G>A	S6_1839684	-		
rs870805126	9	55621577	-:C>G	S9_55621577	-		
rs870823050	8	62018224	-:T>A	S8_62018224	-		
rs870828549	8	12106607	-:C>G	S8_12106607	-		
rs870828815	7	6978411	-:C>T	S7_6978411	-		
rs870835696	7	56568292	-:A>C	S7_56568292	-		
rs870836224	7	57216307	-:T>A	S7_57216307	-		
rs870839059	9	49976288	-:T>A	S9_49976288	-		
rs870843033	9	3983203	-:G>T	S9_3983203	-		
rs870861061	6	47698186	-:T>C	S6_47698186	-		
rs870878740	4	48058365	-:G>A	S4_48058365	-		
rs870883017	4	47966844	-:G>C	S4_47966844	-		
rs870891111	5	16130299	-:T>G	S5_16130299	-		
rs870892719	9	56611795	-:T>A	S9_56611795	-		
rs870922853	6	48609960	-:C>T	S6_48609960	-		
rs870924284	3	59634905	-:C>T	S3_59634905	-		
rs870931910	3	59004261	-:G>A	S3_59004261	-		
rs870932578	2	61698343	-:T>G	S2_61698343	-		
rs870933330	4	57435707	-:C>T	S4_57435707	-		
rs870937824	6	50391547	-:A>G	S6_50391547	-		
rs870948751	2	74755655	-:A>G	S2_74755655	-		
rs870959378	10	54440902	-:T>C	S10_54440902	-		
rs870961406	3	71645716	-:A>G	S3_71645716	-		
rs870962929	7	60741519	-:G>C	S7_60741519	-		
rs870976823	10	58806668	-:A>C	S10_58806668	-		
rs870977593	9	48288282	-:A>C	S9_48288282	-		
rs871017519	9	52930421	-:T>C	S9_52930421	-		
rs871023497	4	5406793	-:A>C	S4_5406793	-		
rs871031296	8	3380758	-:T>G	S8_3380758	-		
rs871031973	8	57430174	-:A>G	S8_57430174	-		
rs871033546	1	7532439	-:C>T	S1_7532439	-		
rs871034881	1	18046915	-:T>A	S1_18046915	-		
rs871039843	9	6801473	-:A>G	S9_6801473	-		
rs871043018	5	16221873	-:G>C	S5_16221873	-		
rs871052251	3	70463489	-:A>T	S3_70463489	-		
rs871069237	9	971733	-:C>T	S9_971733	-		
rs871078346	2	75294037	-:A>G	S2_75294037	-		
rs871080427	2	5261280	-:T>C	S2_5261280	-		
rs871116896	4	9866471	-:C>G	S4_9866471	-		
rs871128512	4	54998196	-:G>C	S4_54998196	-		
rs871134885	3	13649684	-:G>A	S3_13649684	-		

rs871140283	7	57679482	-:G>A	S7_57679482	-		
rs871143136	4	67850119	-:G>A	S4_67850119	-		
rs871143546	4	52608924	-:C>T	S4_52608924	-		
rs871143873	7	807393	-:A>T	S7_807393	-		
rs871149140	2	58220594	-:A>C	S2_58220594	-		
rs871160066	1	78805128	-:A>G	S1_78805128	-		
rs871169466	9	8932682	-:C>T	S9_8932682	-		
rs871176286	7	57224513	-:G>T	S7_57224513	-		
rs871189660	10	767828	-:G>C	S10_767828	-		
rs871200362	10	4058551	-:C>T	S10_4058551	-		
rs871206170	9	54111208	-:C>T	S9_54111208	-		
rs871208179	2	70348871	-:A>G	S2_70348871	-		
rs871223606	9	49940810	-:C>T	S9_49940810	-		
rs871227305	6	50932807	-:A>C	S6_50932807	-		
rs871236114	2	8445210	-:C>G	S2_8445210	-		
rs871237226	1	4043395	-:G>C	S1_4043395	-		
rs871241441	7	4015206	-:A>G	S7_4015206	-		
rs871242819	4	10304304	-:T>C	S4_10304304	-		
rs871243261	4	7389005	-:T>C	S4_7389005	-		
rs871270068	2	73437505	-:C>T	S2_73437505	-		
rs871270379	10	7044477	-:C>G	S10_7044477	-		
rs871271026	6	29539381	-:C>G	S6_29539381	-		
rs871274401	10	13680629	-:T>C	S10_13680629	-		
rs871275920	1	80353845	-:T>A	S1_80353845	-		
rs871276799	6	48366168	-:T>C	S6_48366168	-		
rs871293426	2	59151234	-:T>C	S2_59151234	-		
rs871295859	3	74135699	-:A>T	S3_74135699	-		
rs871300149	1	17450931	-:G>A	S1_17450931	-		
rs871307567	6	47468021	-:C>T	S6_47468021	-		
rs871327197	2	58614110	-:A>G	S2_58614110	-		
rs871337725	3	58926088	-:C>T	S3_58926088	-		
rs871352442	7	3016449	-:G>A	S7_3016449	-		
rs871368746	4	33326536	-:C>T	S4_33326536	-		
rs871374156	2	67571688	-:C>T	S2_67571688	-		
rs871400256	8	54796806	-:T>C	S8_54796806	-		
rs871407955	3	58442689	-:C>A	S3_58442689	-		
rs871411679	3	6139314	-:T>C	S3_6139314	-		
rs871421800	9	51628361	-:T>G	S9_51628361	-		
rs871432418	9	53037477	-:G>C	S9_53037477	-		
rs871444914	10	6453266	-:T>C	S10_6453266	-		
rs871444995	9	1774195	-:G>A	S9_1774195	-		
rs871453894	5	71672875	-:T>A	S5_71672875	-		
rs871454013	6	53003446	-:C>T	S6_53003446	-		

rs871478664	3	71605396	-:A>G	S3_71605396	-		
rs871484742	10	56183874	-:T>G	S10_56183874	-		
rs871487192	5	69861647	-:C>T	S5_69861647	-		
rs871494076	3	3386887	-:A>T	S3_3386887	-		
rs871503681	2	75749557	-:A>T	S2_75749557	-		
rs871511791	2	60131028	-:C>T	S2_60131028	-		
rs871514189	2	70331106	-:A>G	S2_70331106	-		
rs871520418	10	11562266	-:C>T	S10_11562266	-		
rs871522648	6	47601938	-:G>A	S6_47601938	-		
rs871540871	2	9829260	-:A>G	S2_9829260	-		
rs871544849	10	33271371	-:T>A	S10_33271371	-		
rs871546694	2	20563273	-:T>G	S2_20563273	-		
rs871550816	4	8776939	-:C>G	S4_8776939	-		
rs871557726	6	45810611	-:C>T	S6_45810611	-		
rs871557971	2	73452781	-:G>T	S2_73452781	-		
rs871560411	1	61639729	-:C>T	S1_61639729	-		
rs871580649	9	8702778	-:G>C	S9_8702778	-		
rs871600104	9	58838486	-:C>A	S9_58838486	-		
rs871604769	3	66095714	-:A>G	S3_66095714	-		
rs871613090	7	60766424	-:A>G	S7_60766424	-		
rs871652757	2	62878377	-:T>G	S2_62878377	-		
rs871666991	10	55843962	-:A>T	S10_55843962	-		
rs871678329	3	58593101	-:A>G	S3_58593101	-		
rs871682442	9	7110041	-:A>C	S9_7110041	-		
rs871704096	9	7342893	-:G>T	S9_7342893	-		
rs871722742	10	4789680	-:C>T	S10_4789680	-		
rs871723163	4	7965849	-:G>A	S4_7965849	-		
rs871724736	5	2948618	-:A>C	S5_2948618	-		
rs871739727	1	63672106	-:A>G	S1_63672106	-		
rs871742634	3	59262459	-:G>C	S3_59262459	-		
rs871743365	2	62692570	-:A>T	S2_62692570	-		
rs871745619	4	10062194	-:C>G	S4_10062194	-		
rs871751770	1	29690189	-:C>T	S1_29690189	-		
rs871757522	5	62918887	-:T>G	S5_62918887	-		
rs871760962	7	6969051	-:A>G	S7_6969051	-		
rs871760968	3	11642347	-:G>A	S3_11642347	-		
rs871772754	4	6903254	-:C>T	S4_6903254	-		
rs871773385	2	1991240	-:T>C	S2_1991240	-		
rs871786988	2	62062296	-:C>G	S2_62062296	-		
rs871809871	4	9770414	-:T>A	S4_9770414	-		
rs871819753	4	42197033	-:A>G	S4_42197033	-		
rs871822598	3	73184124	-:C>G	S3_73184124	-		
rs871825759	3	7846440	-:T>C	S3_7846440	-		

rs871830250	3	6492287	-:C>T	S3_6492287	-			
rs871835135	9	53720743	-:C>G	S9_53720743	-			
rs871876608	10	57104374	-:C>A	S10_57104374	-			
rs871885324	1	64962315	-:A>G	S1_64962315	-			
rs871885924	4	3886862	-:A>G	S4_3886862	-			
rs871890862	3	72125603	-:A>G	S3_72125603	-			
rs871926500	6	44610491	-:G>A	S6_44610491	-			
rs871927211	8	53944743	-:A>G	S8_53944743	-			
rs871931595	2	8263879	-:G>A	S2_8263879	-			
rs871973865	4	9020496	-:G>A	S4_9020496	-			
rs871974108	3	69905950	-:A>G	S3_69905950	-			
rs871990902	10	48208212	-:A>C	S10_48208212	-			
rs872019742	1	27162632	-:C>A	S1_27162632	-			
rs872028422	1	21473392	-:G>A	S1_21473392	-			
rs872040000	2	58219774	-:G>A	S2_58219774	-			
rs872040956	2	62973742	-:A>C	S2_62973742	-			
rs872046983	2	58880705	-:A>G	S2_58880705	-			
rs872083808	6	50508544	-:A>T	S6_50508544	-			
rs872088664	10	753930	-:G>A	S10_753930	-			
rs872123464	6	56654425	-:C>T	S6_56654425	-			
rs872132323	4	48852813	-:G>C	S4_48852813	-			
rs872144946	2	76876734	-:A>G	S2_76876734	-			
rs872147293	2	59948117	-:T>C	S2_59948117	-			
rs872192986	3	70244855	-:T>G	S3_70244855	-			
rs872215150	10	60053791	-:C>T	S10_60053791	-			
rs872233941	2	10295078	-:T>C	S2_10295078	-			
rs872237985	6	35658116	-:G>A	S6_35658116	-			
rs872240170	3	57319325	-:A>G	S3_57319325	-			
rs872266512	9	53218580	-:C>G	S9_53218580	-			
rs872270872	6	51096872	-:A>G	S6_51096872	-			
rs872273895	6	49616321	-:C>G	S6_49616321	-			
rs872274999	8	44200101	-:C>G	S8_44200101	-			
rs872276264	10	635046	-:A>T	S10_635046	-			
rs872283340	1	22275691	-:G>C	S1_22275691	-			
rs872286683	4	51448955	-:A>G	S4_51448955	-			
rs872295764	4	57558567	-:G>C	S4_57558567	-			
rs872315665	2	6916717	-:A>G	S2_6916717	-			
rs872326133	3	13426527	-:G>T	S3_13426527	-			
rs872327452	3	8021574	-:G>A	S3_8021574	-			
rs872347862	9	363408	-:G>A	S9_363408	-			
rs872370344	2	64314129	-:G>A	S2_64314129	-			
rs872384845	6	60051100	-:T>G	S6_60051100	-			
rs872399247	6	58913126	-:T>C	S6_58913126	-			

rs872404761	7	56473815	-:G>C	S7_56473815	-		
rs872412203	10	5055017	-:A>G	S10_5055017	-		
rs872414427	10	24702539	-:C>T	S10_24702539	-		
rs872425339	9	52377560	-:A>G	S9_52377560	-		
rs872428187	4	4772304	-:C>T	S4_4772304	-		
rs872446758	3	67938556	-:T>C	S3_67938556	-		
rs872450939	1	55376924	-:G>A	S1_55376924	-		
rs872453789	8	58556466	-:T>C	S8_58556466	-		
rs872488790	8	3231972	-:G>A	S8_3231972	-		
rs872499351	6	33541147	-:A>C	S6_33541147	-		
rs872503709	2	62829762	-:G>C	S2_62829762	-		
rs872511680	3	56125216	-:T>C	S3_56125216	-		
rs872521944	3	67584727	-:G>A	S3_67584727	-		
rs872536182	2	76465347	-:C>T	S2_76465347	-		
rs872546000	10	59121067	-:C>A	S10_59121067	-		
rs872549886	1	19645395	-:A>G	S1_19645395	-		
rs872557676	10	6236371	-:C>T	S10_6236371	-		
rs872558914	1	18104582	-:A>G	S1_18104582	-		
rs872575676	2	27766598	-:G>A	S2_27766598	-		
rs872595119	3	70538008	-:C>T	S3_70538008	-		
rs872610905	4	2598709	-:G>A	S4_2598709	-		
rs872614612	5	62383594	-:T>C	S5_62383594	-		
rs872617820	2	73437297	-:G>C	S2_73437297	-		
rs872629963	2	23010733	-:G>A	S2_23010733	-		
rs872639684	9	42811586	-:A>G	S9_42811586	-		
rs872640340	2	61999176	-:T>A	S2_61999176	-		
rs872647530	7	62963950	-:C>T	S7_62963950	-		
rs872678259	9	51277755	-:T>G	S9_51277755	-		
rs872679650	10	8233615	-:G>T	S10_8233615	-		
rs872682716	1	18708936	-:C>T	S1_18708936	-		
rs872694567	4	16122579	-:C>T	S4_16122579	-		
rs872695121	4	57477945	-:G>T	S4_57477945	-		
rs872696743	2	59030845	-:G>A	S2_59030845	-		
rs872714016	2	64391996	-:A>G	S2_64391996	-		
rs872718138	7	62025593	-:G>A	S7_62025593	-		
rs872734854	5	59436380	-:G>A	S5_59436380	-		
rs872776417	3	59293895	-:T>C	S3_59293895	-		
rs872784130	2	12411222	-:T>C	S2_12411222	-		
rs872784490	7	63002461	-:C>T	S7_63002461	-		
rs872788435	9	50873131	-:A>T	S9_50873131	-		
rs872798174	1	66406564	-:A>T	S1_66406564	-		
rs872807089	5	9183937	-:G>A	S5_9183937	-		
rs872811050	3	70976162	-:A>G	S3_70976162	-		

rs872813494	3	71275540	-:C>T	S3_71275540	-		
rs872832214	1	24697600	-:T>G	S1_24697600	-		
rs872834626	10	60572192	-:T>C	S10_60572192	-		
rs872844718	7	17092963	-:T>C	S7_17092963	-		
rs872903827	6	47420922	-:A>G	S6_47420922	-		
rs872918858	5	9535556	-:C>T	S5_9535556	-		
rs872920487	9	49965065	-:A>C	S9_49965065	-		
rs872932934	5	65934261	-:G>C	S5_65934261	-		
rs872933677	6	30771060	-:A>C	S6_30771060	-		
rs872941138	1	63860025	-:A>G	S1_63860025	-		
rs872958918	2	75271667	-:T>G	S2_75271667	-		
rs872967659	4	4239262	-:T>C	S4_4239262	-		
rs872973320	2	64589710	-:A>T	S2_64589710	-		
rs872978284	5	10194503	-:A>G	S5_10194503	-		
rs872979980	3	59201948	-:C>G	S3_59201948	-		
rs872983200	4	56869166	-:A>G	S4_56869166	-		
rs873010314	3	3816915	-:T>G	S3_3816915	-		
rs873018961	2	1408287	-:T>C	S2_1408287	-		
rs873024845	9	584547	-:G>T	S9_584547	-		
rs873032437	8	9721263	-:T>G	S8_9721263	-		
rs873043795	3	8440493	-:T>C	S3_8440493	-		
rs873050799	5	66627710	-:G>C	S5_66627710	-		
rs873052524	5	68811663	-:C>T	S5_68811663	-		
rs873054338	5	639064	-:C>G	S5_639064	-		
rs873055144	4	12331532	-:A>G	S4_12331532	-		
rs873077366	4	37656059	-:G>A	S4_37656059	-		
rs873084114	1	63399217	-:C>A	S1_63399217	-		
rs873089185	3	54444829	-:A>G	S3_54444829	-		
rs873095133	9	45637088	-:C>G	S9_45637088	-		
rs873102246	2	18990434	-:A>G	S2_18990434	-		
rs873106350	7	12049713	-:G>A	S7_12049713	-		
rs873113321	7	59045787	-:G>C	S7_59045787	-		
rs873116379	1	57491270	-:G>T	S1_57491270	-		
rs873120301	7	58325570	-:T>C	S7_58325570	-		
rs873127975	5	2696571	-:A>T	S5_2696571	-		
rs873141820	5	5702314	-:C>G	S5_5702314	-		
rs873146630	9	55511609	-:C>G	S9_55511609	-		
rs873179773	4	10367165	-:A>G	S4_10367165	-		
rs873182223	3	73046867	-:T>C	S3_73046867	-		
rs873185491	8	4462441	-:T>A	S8_4462441	-		
rs873189267	6	45215330	-:C>T	S6_45215330	-		
rs873193839	2	1415713	-:C>T	S2_1415713	-		
rs873194706	9	56646884	-:C>A	S9_56646884	-		

rs873195853	7	50318042	-:T>A	S7_50318042	-		
rs873213553	6	51443231	-:G>A	S6_51443231	-		
rs873215122	3	58678154	-:A>G	S3_58678154	-		
rs873217316	1	55816748	-:C>G	S1_55816748	-		
rs873225295	2	57981108	-:G>T	S2_57981108	-		
rs873240013	1	24703668	-:G>A	S1_24703668	-		
rs873245356	3	11734679	-:G>C	S3_11734679	-		
rs873249698	4	10767260	-:G>T	S4_10767260	-		
rs873252741	10	7463503	-:G>A	S10_7463503	-		
rs873260805	9	54904001	-:A>G	S9_54904001	-		
rs873271604	3	66788731	-:T>C	S3_66788731	-		
rs873280854	9	41600666	-:C>T	S9_41600666	-		
rs873282932	7	55105367	-:T>C	S7_55105367	-		
rs873289736	4	38494454	-:C>T	S4_38494454	-		
rs873290562	4	59009985	-:G>T	S4_59009985	-		
rs873295307	9	52777268	-:G>C	S9_52777268	-		
rs873300662	2	63927796	-:A>T	S2_63927796	-		
rs873301005	2	45977261	-:A>G	S2_45977261	-		
rs873328394	3	52637590	-:C>T	S3_52637590	-		
rs873334166	1	19645514	-:T>G	S1_19645514	-		
rs873336873	9	1615860	-:A>G	S9_1615860	-		
rs873342635	7	60297748	-:T>C	S7_60297748	-		
rs873346720	8	1641762	-:A>G	S8_1641762	-		
rs873352936	2	56904578	-:C>A	S2_56904578	-		
rs873356901	2	74725744	-:G>A	S2_74725744	-		
rs873358030	9	1190784	-:C>A	S9_1190784	-		
rs873386214	6	48799922	-:A>G	S6_48799922	-		
rs873417659	4	53075595	-:G>A	S4_53075595	-		
rs873447684	7	62904122	-:C>G	S7_62904122	-		
rs873472296	4	51083862	-:C>T	S4_51083862	-		
rs873477343	7	64913073	-:A>G	S7_64913073	-		
rs873487115	9	2672406	-:T>C	S9_2672406	-		
rs873491030	1	50780871	-:C>T	S1_50780871	-		
rs873511802	6	56896888	-:G>T	S6_56896888	-		
rs873526818	2	56563796	-:A>C	S2_56563796	-		
rs873530985	5	5249161	-:G>C	S5_5249161	-		
rs873543095	6	55870408	-:T>C	S6_55870408	-		
rs873549217	3	70276670	-:T>G	S3_70276670	-		
rs873581071	3	2124787	-:T>C	S3_2124787	-		
rs873581582	1	75797128	-:G>C	S1_75797128	-		
rs873581730	1	4778713	-:G>A	S1_4778713	-		
rs873584321	2	13177508	-:T>A	S2_13177508	-		
rs873592494	2	72181200	-:A>C	S2_72181200	-		

rs873594095	6	45491360	-:A>C	S6_45491360	-			
rs873598206	5	71688634	-:G>C	S5_71688634	-			
rs873613835	9	10639975	-:G>A	S9_10639975	-			
rs873614720	7	55519166	-:A>G	S7_55519166	-			
rs873618828	9	57233591	-:G>A	S9_57233591	-			
rs873637432	4	8677731	-:G>C	S4_8677731	-			
rs873645052	8	42230584	-:C>T	S8_42230584	-			
rs873665900	8	57601724	-:A>G	S8_57601724	-			
rs873686315	7	63018383	-:A>G	S7_63018383	-			
rs873687281	4	52349971	-:T>C	S4_52349971	-			
rs873689496	2	55790962	-:T>A	S2_55790962	-			
rs873691998	4	56026698	-:G>A	S4_56026698	-			
rs873729773	9	51248457	-:T>G	S9_51248457	-			
rs873736425	5	64281775	-:C>G	S5_64281775	-			
rs873748849	6	56759309	-:G>A	S6_56759309	-			
rs873753645	5	60680549	-:T>C	S5_60680549	-			
rs873768240	8	58509620	-:T>C	S8_58509620	-			
rs873768367	6	3312978	-:A>G	S6_3312978	-			
rs873771177	7	59597652	-:G>A	S7_59597652	-			
rs873777633	4	6425729	-:T>G	S4_6425729	-			
rs873784196	7	63075893	-:T>A	S7_63075893	-			
rs873785644	1	25465803	-:T>A	S1_25465803	-			
rs873792071	1	62670578	-:C>T	S1_62670578	-			
rs873810589	9	56441806	-:C>A	S9_56441806	-			
rs873824092	9	4947142	-:G>C	S9_4947142	-			
rs873828862	4	56086267	-:T>A	S4_56086267	-			
rs873829172	6	41827772	-:A>G	S6_41827772	-			
rs873832150	3	66003612	-:C>T	S3_66003612	-			
rs873835018	10	60106142	-:C>G	S10_60106142	-			
rs873839773	2	64562615	-:G>T	S2_64562615	-			
rs873840156	3	65353632	-:G>T	S3_65353632	-			
rs873848252	3	9057564	-:A>G	S3_9057564	-			
rs873867871	2	60969241	-:T>G	S2_60969241	-			
rs873869392	3	5443727	-:C>T	S3_5443727	-			
rs873872655	10	52924958	-:G>C	S10_52924958	-			
rs873873936	10	7522564	-:T>C	S10_7522564	-			
rs873877281	5	64424670	-:G>A	S5_64424670	-			
rs873879001	2	2781003	-:G>C	S2_2781003	-			
rs873883616	1	11972472	-:C>T	S1_11972472	-			
rs873885228	2	59985245	-:T>G	S2_59985245	-			
rs873889158	1	53393766	-:T>C	S1_53393766	-			
rs873912659	2	56651906	-:C>T	S2_56651906	-			
rs873913560	1	25122403	-:A>G	S1_25122403	-			

rs873919813	4	59019567	-:C>T	S4_59019567	-		
rs873929829	3	10279790	-:G>C	S3_10279790	-		
rs873943014	7	65418956	-:A>G	S7_65418956	-		
rs873944133	5	64411841	-:G>C	S5_64411841	-		
rs873947707	4	57039886	-:T>G	S4_57039886	-		
rs873968862	10	1099668	-:C>T	S10_1099668	-		
rs873985063	2	22756856	-:T>C	S2_22756856	-		
rs873988741	2	58752330	-:T>A	S2_58752330	-		
rs873992822	7	11866233	-:G>A	S7_11866233	-		
rs874000593	3	73128685	-:G>C	S3_73128685	-		
rs874012247	5	9396890	-:G>T	S5_9396890	-		
rs874028818	3	70553794	-:C>T	S3_70553794	-		
rs874030268	8	54085198	-:T>C	S8_54085198	-		
rs874032128	2	60973403	-:C>T	S2_60973403	-		
rs874059611	2	4286577	-:A>G	S2_4286577	-		
rs874071182	3	53034689	-:C>T	S3_53034689	-		
rs874080981	4	1603553	-:A>T	S4_1603553	-		
rs874091938	2	21781371	-:C>T	S2_21781371	-		
rs874120910	10	666136	-:G>C	S10_666136	-		
rs874124107	7	56973244	-:A>C	S7_56973244	-		
rs874142904	4	56083846	-:C>G	S4_56083846	-		
rs874157543	2	57875126	-:C>T	S2_57875126	-		
rs874168380	5	4616881	-:T>G	S5_4616881	-		
rs874183170	4	58923566	-:A>G	S4_58923566	-		
rs874186141	5	61615958	-:T>A	S5_61615958	-		
rs874200755	7	62656349	-:C>T	S7_62656349	-		
rs874229195	6	45715649	-:T>C	S6_45715649	-		
rs874245216	6	47185463	-:A>C	S6_47185463	-		
rs874246420	7	61947225	-:A>G	S7_61947225	-		
rs874251344	2	6542125	-:A>G	S2_6542125	-		
rs874253950	7	61710185	-:C>A	S7_61710185	-		
rs874254254	1	1985365	-:T>G	S1_1985365	-		
rs874257585	7	10885094	-:C>G	S7_10885094	-		
rs874260708	2	6784211	-:C>T	S2_6784211	-		
rs874267316	2	20099761	-:C>T	S2_20099761	-		
rs874271268	1	56200183	-:G>A	S1_56200183	-		
rs874272873	2	12509675	-:T>C	S2_12509675	-		
rs874279973	3	7871837	-:C>G	S3_7871837	-		
rs874282279	8	792065	-:C>A	S8_792065	-		
rs874291341	9	9886468	-:T>G	S9_9886468	-		
rs874299705	4	1312635	-:C>A	S4_1312635	-		
rs874309712	3	69068844	-:T>G	S3_69068844	-		
rs874310716	8	45237438	-:G>A	S8_45237438	-		

rs874311894	2	8715911	-:T>G	S2_8715911	-		
rs874358086	7	58732819	-:T>G	S7_58732819	-		
rs874359764	2	52186362	-:A>G	S2_52186362	-		
rs874377260	1	21945653	-:G>T	S1_21945653	-		
rs874387586	2	73375802	-:A>T	S2_73375802	-		
rs874416420	2	63356162	-:A>G	S2_63356162	-		
rs874423584	9	10332587	-:A>G	S9_10332587	-		
rs874445109	2	56957125	-:C>T	S2_56957125	-		
rs874451487	8	50606728	-:G>A	S8_50606728	-		
rs874455668	3	8077286	-:G>C	S3_8077286	-		
rs874462166	5	14864088	-:G>C	S5_14864088	-		
rs874465859	3	70559049	-:C>T	S3_70559049	-		
rs874481872	6	59030350	-:C>A	S6_59030350	-		
rs874493093	3	70147980	-:G>C	S3_70147980	-		
rs874494323	9	200938	-:C>T	S9_200938	-		
rs874496051	2	77145283	-:G>T	S2_77145283	-		
rs874496226	9	3979525	-:T>C	S9_3979525	-		
rs874504467	1	52747860	-:A>G	S1_52747860	-		
rs874510433	5	55294626	-:T>C	S5_55294626	-		
rs874522511	9	7404092	-:T>A	S9_7404092	-		
rs874526754	1	24819224	-:G>T	S1_24819224	-		
rs874526839	4	62060478	-:C>A	S4_62060478	-		
rs874535647	9	56507394	-:A>G	S9_56507394	-		
rs874555784	10	7858623	-:G>A	S10_7858623	-		
rs874589956	1	31179618	-:G>A	S1_31179618	-		
rs874591277	4	41906870	-:G>A	S4_41906870	-		
rs874593798	5	69042550	-:T>A	S5_69042550	-		
rs874605349	2	70250459	-:G>T	S2_70250459	-		
rs874644014	2	65456299	-:C>G	S2_65456299	-		
rs874663901	2	49535651	-:G>A	S2_49535651	-		
rs874669554	10	59627499	-:G>C	S10_59627499	-		
rs874693418	2	70339174	-:C>A	S2_70339174	-		
rs874708357	10	55990186	-:G>C	S10_55990186	-		
rs874736435	7	62895081	-:T>C	S7_62895081	-		
rs874737912	3	56161768	-:G>T	S3_56161768	-		
rs874739740	6	48811095	-:T>C	S6_48811095	-		
rs874766572	1	778962	-:A>G	S1_778962	-		
rs874766810	7	56398552	-:C>T	S7_56398552	-		
rs874778284	7	14397195	-:A>G	S7_14397195	-		
rs874812939	3	71018229	-:A>G	S3_71018229	-		
rs874815885	1	11107581	-:A>T	S1_11107581	-		
rs874824530	8	51549715	-:G>A	S8_51549715	-		
rs874839880	6	52515226	-:G>T	S6_52515226	-		

rs874846597	2	73638393	-:T>C	S2_73638393	-		
rs874853606	3	4244979	-:C>A	S3_4244979	-		
rs874861065	8	7071341	-:T>C	S8_7071341	-		
rs874863694	4	14234536	-:G>C	S4_14234536	-		
rs874863964	7	62907311	-:C>T	S7_62907311	-		
rs874865986	10	51779766	-:C>A	S10_51779766	-		
rs874867561	1	56463015	-:G>T	S1_56463015	-		
rs874875679	8	58332517	-:T>G	S8_58332517	-		
rs874893650	2	74442614	-:G>A	S2_74442614	-		
rs874902785	4	56236474	-:G>T	S4_56236474	-		
rs874920528	1	72766589	-:G>C	S1_72766589	-		
rs874932595	3	73010845	-:G>A	S3_73010845	-		
rs874941162	6	50285828	-:T>G	S6_50285828	-		
rs874944171	1	51456072	-:T>C	S1_51456072	-		
rs874960651	3	116883	-:C>T	S3_116883	-		
rs874970617	2	53886993	-:C>T	S2_53886993	-		
rs874971207	4	3763523	-:G>A	S4_3763523	-		
rs874972902	3	59637582	-:T>A	S3_59637582	-		
rs874980126	3	3954113	-:C>T	S3_3954113	-		
rs875000124	9	50609301	-:A>G	S9_50609301	-		
rs875001482	1	72507787	-:T>A	S1_72507787	-		
rs875009345	8	58571701	-:C>G	S8_58571701	-		
rs875011537	2	62009910	-:C>G	S2_62009910	-		
rs875014386	10	4466700	-:C>T	S10_4466700	-		
rs875045348	9	50101834	-:A>G	S9_50101834	-		
rs875053040	6	50077855	-:C>A	S6_50077855	-		
rs875058290	3	7843617	-:C>T	S3_7843617	-		
rs875061793	4	56086908	-:A>C	S4_56086908	-		
rs875067780	5	61616018	-:C>T	S5_61616018	-		
rs875081447	3	7046327	-:G>A	S3_7046327	-		
rs875083660	9	57899538	-:C>T	S9_57899538	-		
rs875092863	3	69803838	-:G>T	S3_69803838	-		
rs875135914	10	6027541	-:A>G	S10_6027541	-		
rs875138694	1	14325095	-:C>T	S1_14325095	-		
rs875139480	3	61301443	-:C>G	S3_61301443	-		
rs875154235	5	2915387	-:A>C	S5_2915387	-		
rs875154787	2	50018400	-:G>A	S2_50018400	-		
rs875157462	6	49470085	-:A>G	S6_49470085	-		
rs875164634	7	4014254	-:G>T	S7_4014254	-		
rs875170723	6	46381381	-:G>C	S6_46381381	-		
rs875177013	5	8789731	-:G>A	S5_8789731	-		
rs875179300	4	50962212	-:T>C	S4_50962212	-		
rs875180360	10	5199326	-:T>A	S10_5199326	-		

rs875203989	1	4943775	-:G>A	S1_4943775	-		
rs875231184	2	56068692	-:A>C	S2_56068692	-		
rs875256651	2	18921102	-:T>C	S2_18921102	-		
rs875267355	2	62918770	-:G>C	S2_62918770	-		
rs875272872	6	246208	-:C>A	S6_246208	-		
rs875302298	6	632992	-:T>C	S6_632992	-		
rs875311862	4	51780389	-:T>G	S4_51780389	-		
rs875312243	4	4189597	-:A>G	S4_4189597	-		
rs875319296	10	49621752	-:G>A	S10_49621752	-		
rs875356748	5	59574508	-:G>A	S5_59574508	-		
rs875361951	10	9676386	-:C>A	S10_9676386	-		
rs875368334	7	3698780	-:G>C	S7_3698780	-		
rs875383793	1	65451869	-:G>T	S1_65451869	-		
rs875397820	10	57306778	-:C>G	S10_57306778	-		
rs875400081	4	37913379	-:T>A	S4_37913379	-		
rs875440055	8	62221039	-:A>G	S8_62221039	-		
rs875444294	3	60925537	-:C>T	S3_60925537	-		
rs875456380	6	36778682	-:G>C	S6_36778682	-		
rs875467659	8	3230406	-:G>C	S8_3230406	-		
rs875473180	4	66606618	-:G>T	S4_66606618	-		
rs875481707	5	55269947	-:G>T	S5_55269947	-		
rs875482196	4	5002524	-:G>T	S4_5002524	-		
rs875496313	1	55513841	-:C>A	S1_55513841	-		
rs875501555	3	7845949	-:T>C	S3_7845949	-		
rs875501798	4	61442775	-:A>C	S4_61442775	-		
rs875522506	2	73108734	-:C>T	S2_73108734	-		
rs875525094	8	60385385	-:T>G	S8_60385385	-		
rs875525308	9	2498309	-:A>G	S9_2498309	-		
rs875535711	9	20409022	-:C>T	S9_20409022	-		
rs875535838	2	51634878	-:A>G	S2_51634878	-		
rs875548580	10	18070625	-:G>A	S10_18070625	-		
rs875550965	2	3937345	-:G>A	S2_3937345	-		
rs875588188	6	51034813	-:G>A	S6_51034813	-		
rs875592630	3	73842973	-:G>C	S3_73842973	-		
rs875594898	2	6921543	-:T>A	S2_6921543	-		
rs875596678	7	54718460	-:T>C	S7_54718460	-		
rs875600565	4	56534234	-:C>G	S4_56534234	-		
rs875616638	2	55203289	-:C>G	S2_55203289	-		
rs875630273	6	46386473	-:T>C	S6_46386473	-		
rs875631783	4	10117207	-:T>C	S4_10117207	-		
rs875644050	1	11764852	-:T>C	S1_11764852	-		
rs875650245	3	71866553	-:G>A	S3_71866553	-		
rs875651274	8	4289984	-:G>C	S8_4289984	-		

rs875661912	9	8888816	-:A>G	S9_8888816	-		
rs875664905	6	43776021	-:C>T	S6_43776021	-		
rs875666189	1	10080152	-:G>A	S1_10080152	-		
rs875667568	3	8071735	-:T>C	S3_8071735	-		
rs875668266	2	10331565	-:T>C	S2_10331565	-		
rs875676669	7	807355	-:C>A	S7_807355	-		
rs875705288	8	55843562	-:A>T	S8_55843562	-		
rs875718767	10	7878447	-:T>C	S10_7878447	-		
rs875736463	7	63628557	-:A>C	S7_63628557	-		
rs875761898	3	59723404	-:C>A	S3_59723404	-		
rs875774664	8	58556214	-:G>T	S8_58556214	-		
rs875778433	6	50944419	-:C>A	S6_50944419	-		
rs875778903	7	54307640	-:A>G	S7_54307640	-		
rs875780045	8	5158604	-:C>G	S8_5158604	-		
rs875782342	1	24621721	-:C>G	S1_24621721	-		
rs875792586	2	53326465	-:A>G	S2_53326465	-		
rs875800729	7	39089736	-:G>C	S7_39089736	-		
rs875802705	1	30825641	-:T>G	S1_30825641	-		
rs875807591	3	922016	-:G>T	S3_922016	-		
rs875818796	6	48849974	-:T>C	S6_48849974	-		
rs875820750	5	55912013	-:T>G	S5_55912013	-		
rs875827531	6	49451440	-:C>T	S6_49451440	-		
rs875831409	1	61245742	-:A>T	S1_61245742	-		
rs875832690	6	38341725	-:G>C	S6_38341725	-		
rs875832782	6	35572916	-:T>C	S6_35572916	-		
rs875837325	5	12296954	-:T>C	S5_12296954	-		
rs875840069	1	62433561	-:C>G	S1_62433561	-		
rs875840989	3	46805444	-:T>C	S3_46805444	-		
rs875846283	6	57816533	-:C>G	S6_57816533	-		
rs875853953	7	23337292	-:T>C	S7_23337292	-		
rs875863490	2	75431116	-:G>C	S2_75431116	-		
rs875868095	1	75386873	-:C>T	S1_75386873	-		
rs875870844	2	4950883	-:A>G	S2_4950883	-		
rs875871274	1	57941991	-:A>G	S1_57941991	-		
rs875903406	10	52091517	-:C>A	S10_52091517	-		
rs875907883	7	5023381	-:C>T	S7_5023381	-		
rs875916727	1	61250358	-:A>G	S1_61250358	-		
rs875949906	8	6280523	-:A>G	S8_6280523	-		
rs875973530	8	56617626	-:G>C	S8_56617626	-		
rs875973668	2	64909129	-:T>C	S2_64909129	-		
rs875983648	5	20970097	-:T>C	S5_20970097	-		
rs875985334	1	20178914	-:C>G	S1_20178914	-		
rs875986947	2	6232488	-:C>T	S2_6232488	-		

rs162525488	1	9430698	-:C>G	S1_9430698	-		not_in_MDP
rs266949908	2	60162505	-:G>C	S2_60162505	-		not_in_MDP
rs266701451	2	9031226	-:A>T	S2_9031226	-		not_in_MDP
rs162546603	3	6340783	-:T>G	S3_6340783	-		not_in_MDP
rs162440256	4	51654300	-:G>A	S4_51654300	-		not_in_MDP
rs872819886	4	52474062	-:T>A	S4_52474062	-		not_in_MDP
rs266549630	5	36685877	-:T>C	S5_36685877	-		not_in_MDP
rs5980786980	5	45458043	-:T>C	S5_45458043	-		not_in_MDP
rs5980786890	5	45568161	-:T>A	S5_45568161	-		not_in_MDP
rs162441891	7	55105368	-:G>C	S7_55105368	-		not_in_MDP
rs3380207620	7	6284501	-:A>G	S7_6284501	-		not_in_MDP
rs162619317	8	39495	-:A>C	S8_39495	-		not_in_MDP
rs3379589362	8	47586773	-:G>A	S8_47586773	-		not_in_MDP
rs875346562	8	4912128	-:A>T	S8_4912128	-		not_in_MDP
rs266943495	8	59009354	-:C>T	S8_59009354	-		not_in_MDP
rs266781317	8	59009517	-:A>G	S8_59009517	-		not_in_MDP
rs3380483233	10	60186404	-:G>C	S10_60186404	-		not_in_MDP

Implementing Reference SNP Identifiers (rsIDs) to Foster Adoption of FAIR Genetic Variation Data Standards in Agriculture

Objective

FAIR-compliant genetic variation data is essential for accelerating progress in plant breeding, genomics and achieving sustainable agriculture. This guide enables researchers in adopting the standardized practices for generating, archiving and sharing FAIR (Findable, Accessible, Interoperable, and Reusable)-compliant genetic variant data.

The need for standardization of plant variation data

Despite growing volumes of genotyping and sequencing data, the lack of standardized identifiers for genetic variants especially in plants presents significant challenges, including limited data interoperability, inconsistent functional annotation, and difficulty in comparing genetic information across individuals within the same species. Such fragmentation hinders the generation of meaningful biological insights and reusability of data across platforms and research efforts.

To address this gap, the European Variation Archive (EVA) has enabled the assignment of reference SNP identifiers (rsIDs) to non-human variation data, including crops and livestock. An rsID represents a reference SNP cluster ID that uniquely identifies a variable genomic locus. It should be unique, persistent, and stable across genome assemblies and crop varieties. These identifiers serve as essential anchors, connecting variant data to other valuable resources such as population allele and genotype frequencies, Genome-Wide Association Study (GWAS) and Quantitative Trait Locus (QTL) datasets, and trait associations, including both clinical and agronomic traits. The adoption of rsIDs not only enhances the utility of genetic variation data in plants but also lays the foundation for their broader implementation across agricultural research and breeding programs.

Purpose of this guide

This guide enables researchers in adopting the best practices to produce FAIR-compliant variant datasets that are reusable, traceable, interoperable and ready for integration in global genomic resources. This guide answers key questions encountered during SNP data preparation and submission, including:

- How to verify whether existing variants already have assigned rsIDs in the EVA
- How to submit new SNP data to EVA, including scenarios where an accessioned reference genome exists for the species in the International Nucleotide Sequence Database Collaboration (INSDC), and how to submit one if it does not.
- How to ensure that the variant data is provided in a standardized, validated format such as Variant Call Format (VCF) including guidance on VCF metadata requirements (e.g. ##fileformat, ##reference_ac, ##contig) and the use of globally recognized sample

identifiers from major germplasm repositories e.g. BioSamples or GRIN-Global to ensure traceability and interoperability.

By following this recipe, researchers can adopt best practices for producing high quality, FAIR-compliant annotated variation datasets that support sustainable reuse, enable global interoperability and contribute to the growing ecosystem of open and accessible genomic data.

Who should use this guide?

- Agriculture sciences researchers
- Bioinformaticians handling variant data
- Data curators and repository submitters
- Genomics researchers

Inputs Required

- *VCF file*: Contains genotyping results; should include or be annotated with rsIDs
- *Reference genome*: Accessioned at one of the INSDC databases (*i.e.*, the National Center for Biotechnology Information (NCBI), the European Nucleotide Archive (ENA) or the DNA Data Bank of Japan (DDBJ) with GenBank/RefSeq (GCA/GCF) genome assembly ID); chromosome names must match those in the VCF
- *Sample metadata*: MIAPPE-compliant, registered via BioSamples
- *Sequencing data*: Registered and submitted to ENA
- *Project metadata*: Submission metadata (Excel template) for EVA

Tools & Resources

- *EVA Submission Portal*: A web-based interface for submitting the variant data and metadata to EVA. Suitable for users handling small scale datasets.
- *EVA Submission CLI* (Command-Line Interface): Tool for the validation and submission of large datasets to EVA via command-line offers users automated batch processing and scripting capabilities.
- *Annotation Tools:*
    - *SnpEff:* A fast and versatile tool to annotate and predict the effects of genetic variants (e.g., missense, nonsense, synonymous).
    - *bcftools*: A command-line toolkit for manipulating VCF and BCF files, used for filtering, indexing, merging, and basic variant annotation.
    - *Ensembl Variant Effect Predictor (VEP)*: A comprehensive tool for annotating variants with information such as consequence type, gene name, transcript impact, regulatory features, and known variant databases. Often used for large-scale or highly detailed variant annotation.

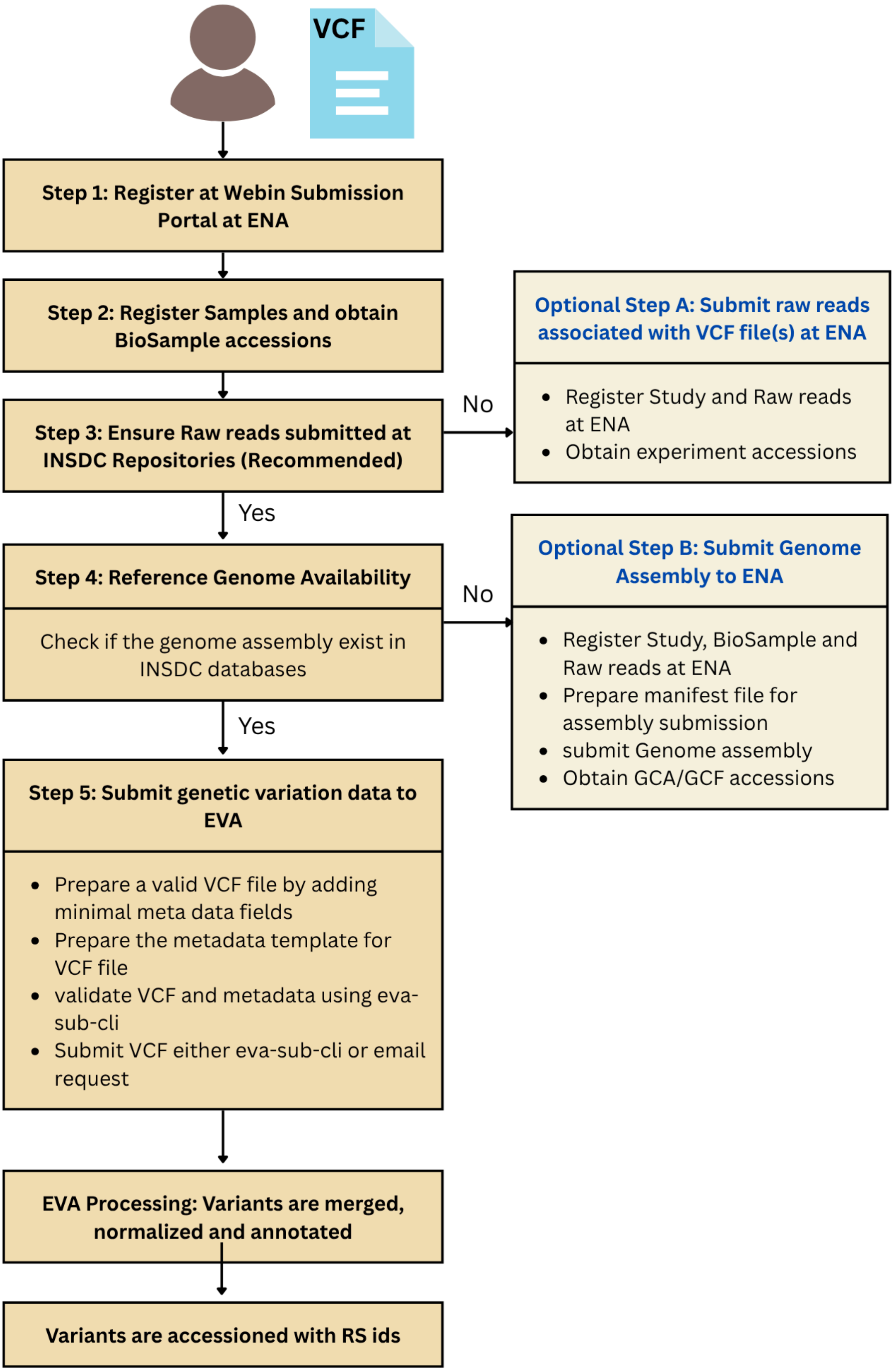

Figure 1. Overview of submission process

Step-by-Step Guide

The European Variation Archive (EVA) is the 'one-stop-shop' for sharing all types of genetic variation data including SNPs, indels and structural variants and responsible for issuing and sustainable maintenance of variant accessions for all non-human species. Users can submit, access and download the genetic variation data at EVA. The EVA is based on direct user submission and variants must be described in the Variant Call Format (VCF; Versions v4.1, v4.2, & v4.3 accepted). Submissions to EVA consist of VCF file(s), associated data file(s) and detailed metadata describing samples(s), experiments(s) and analysis. Follow the steps below to submit your genetic variation data to EVA and associated data at ENA:

Step 1: Register and create your user account at Webin Submission Portal

Webin Submission portal offers a range of submission services as well as reports at ENA for researchers to submit biological sequence data and associated metadata. Register and create your account at Webin Submission Portal by filling a simple registration form. After approval you can login to your account with Webin credentials.

Step 2: Register a Sample

Samples represent the biological source of your sequencing data and are essential for organizing your reads, assemblies, or analyses. Here's how to register them easily using the Webin Submission Portal.

- Log in to your Webin Submission Portal and click on 'Register Samples'.
- Select "Download spreadsheet to register samples" to open the checklist menu.
- Browse and pick the appropriate checklist (e.g., "Plant and Fungi Sample Checklist").
- Customize your checklist by adding relevant fields and click 'Next' followed by downloading the TSV template spreadsheet.
- Fill out the template spreadsheet in excel or google sheets.

See the example below:

scientific_name	Pennisetum glaucum
collection_date	2025-01-15
geographic_location	India: Rajasthan
isolate	ICMB 123

host	*None* (if not applicable)
environment_biome	*agricultural field*
description	*Pearl millet sample used for genome assembly*

- To submit the spreadsheet, go back to the 'Register Sample' section and click on 'Upload filled spreadsheet' to register samples. Upload the file and click 'Submit Completed Spreadsheet'. Upon successful upload you'll receive sample accession numbers (e.g., ERS####### and SAMEA#######).

Step 3: Ensure the submission of associated raw reads to ENA (Recommended)

It is recommended that any associated raw reads should be submitted to INSDC repositories (ENA, NCBI or DDBJ) to ensure traceability, reproducibility, and compliance with FAIR data principles. Submitting raw reads allows downstream users to verify and re-analyze variant calls, enhances the visibility of your dataset, and enables EVA to link variant accessions to primary experimental data. Refer to *optional step A* for submitting raw reads to ENA.

Step 4: Check if the reference genome assembly is available at ENA (or another INSDC database).

The foremost requirement to submit a variant data to EVA is that all variants are submitted with an asserted position on an INSDC accessioned reference sequence. An INSDC sequence refers to any assembled biological sequence, including but not limited to: genome assemblies, transcriptomes, and gene sequences.

Search for your genome assembly at ENA: https://www.ebi.ac.uk/ena/browser.

If the genome assembly is available, proceed to *Step 5*: VCF files to EVA . If the reference genome is not available at ENA, you must submit your genome assembly to an INSDC repository (ENA, GenBank, or DDBJ) as described in Optional S*tep B*: Submit a genome assembly to ENA.

Ensure your reference genome has a **GCF/GCA accession**.
Verify:
Chromosome names match those in ENA.
VCF includes: `##reference=GCA_XXXXXXX.X`

Note: Researchers from countries like India, China, and Korea can submit their data through their respective national data centres, provided these centres broker submissions to one of the INSDC member repositories (ENA, GenBank, or DDBJ). For example, the Indian Nucleotide Data Archive (INDA), hosted by the Indian Biological Data Centre (IBDC), brokers its open-access submissions in real time to the ENA. In this way, any submission will receive both Indian as well as INSDC accessions.

Step 5: Submit the VCF files to the EVA

5.1 Preparation of a valid VCF data file by adding minimal list of metadata fields:

To ensure your VCF file is FAIR-compliant and meets the standards outlined by Beier et al. (2022), following additional fields must be included. These metadata fields improve interoperability, data reuse, and submission compatibility with EVA

- Obligatory meta-information line:

  ##fileformat : file format

  Example:

  ##fileformat=VCFv4.3

- Recommended meta-information lines:
  - o ##fileDate (Date): creation date of the VCF in the basic form without separator: YYYYMMDD.

  - o ##bioinformatics_source (DOI, URL or URI): analytic approach used for creating the VCF file

    Example:

    ##bioinformatics_source="github.com/gramarga/tassel4-poly"

  - o **##reference_ac (assembly_accession):** accession number along with the version of the concerned reference genome.

    Example:

    ##reference_ac=
    https://www.ebi.ac.uk/ena/browser/view/GCA_000003195.3"GCA_000003195.3

  - o **##reference_url (DOI)**: a URL (or URI/DOI) for downloading of the concerned reference genome assembly, preferably from one INSDC archive.

    Example:
    ##reference_url="ftp.ncbi.nlm.nih.gov/genomes/all/GCA/902/498/975/GCA_902498975.1_Morex_v2.0/GCA_902498975.1_Morex_v2.0_genomic.fna.gz"

  - o **Contig field format**: ##contig (<ID=ctg1, length=sequence_length, assembly=gca_accession, md5=md5_hash, species=NCBITaxon:id>); detailed description of individual reference genome sequence

    Example:

    ##contig=<ID=chr1H,length=522466905,assembly=GCA_902498975.1,md5=8d21a35cc68340ecf40e2a8dec9428fa,species=NCBITaxon:4513>

  - o **Sample field format**: ##SAMPLE(<ID=url, ext_ID=$registry:identifier>); description of the concerned sample material present in the VCF file.

Example:
##SAMPLE=<ID=SAMEA104646767,DOI="doi.org/10.25642/IPK/GBIS/7811152”

Please read the [VCF specification] when converting data to VCF in order to ensure a valid format.

5.2 Prepare the metadata associated with VCF file

Next, the user needs to provide the metadata details describing the samples and analysis of the EVA submission for accurate understanding, enhanced visibility and effective use of the data in future. This file will also be validated alongside of VCF files with eve-sub-cli Download the metadata template from the following link:

https://www.ebi.ac.uk/eva/?Submit-Data&src=wizard&wiztype=quicklink.

The form is divided into four sections, Submitter Details, Project, Analysis, Sample. Appropriate metadata for your submission should look similar to the example given below:

**Submitter Details**	Information about User, contact email and its affiliation
**Project**	
Project Title	Whole-genome SNP analysis of Pennisetum glaucum cultivar ICMB 123
Description	This study reports high-confidence SNPs identified through whole-genome sequencing of the ICMB 123 cultivar to support pearl millet genomics and breeding.
Centre	Centre_Name
Publications (DB:ID Format)	PubMed:37298320
Taxonomy ID (as per NCBI taxonomy)	4544 (Pennisetum glaucum)
Parent Project (Accession of parent project)	
Child Projects	
Peer Projects	
Links	
Hold date	
Collaborations	
Broker	

**Analysis**	
Analysis Title	Variant annotation of ICMB 123 using VEP
Analysis Alias	icmb123_vep_analysis_v1
Description	SNPs were functionally annotated using Ensembl VEP with reference genome GCA_902498975.1.
Experiment type	Whole Genome Sequencing (WGS)
Reference	GCA_902498975.1 (Pennisetum glaucum reference genome)
Reference Fasta Path	/path/to/GCA_902498975.1_genomic.fna.gz
Platform	Illumina HiSeq 2500, Oxford Nanopore MinION
Software	GATK v4.3, bcftools, Ensembl VEP v107
Pipeline Description	reads aligned with BWA-MEM; variants called using GATK HaplotypeCaller and annotated using VEP
Imputation	Not Performed
Phasing	Not Performed
Centre	EBI
Date	2025-09-15
Link(s)	
Run Accession(s)	ERR1234567, ERR1234568
**Sample- Mandatory fields**	
Analysis Alias	ICMB123_SNPanalysis_2025
Sample Name	ICMB123_leaf_sample
**Fields for Pre-Registered Sample**	
Sample Accession	SAMEA12345678

If you have not registered your samples, please provide details as per the metadata sample field for novel samples in the metadata template. Otherwise you can first register the samples separately in Biosample and then proceed with VCF submission.

5.3 Submission of a VCF file to EVA

After the preparation of a valid VCF file and metadata template, you are good to go with the submission of your VCF file to EVA following the instructions. Prior to submission, the files can be validated using EVA VCF Validation suite on GitHub. Please go through the “"Getting Started" guide” for detailed instructions.

The eva-sub-cli tool is a command line interface for the data validation and submission.

- Install eva-sub-cli using either of the two ways:
  1. Conda

  Use following command to install eva-sub-cli in a new conda environment called **eva**

  ```
  conda create -n eva -c conda-forge -c bioconda
  eva-sub-cli
  ```

  ```
  conda activate eva
  ```

  ```
  eva-sub-cli.py --help
  ```

  2. Source using Docker

  Ensure you have Python 3.8+ and Docker installed. Use following command to install eva-cli-cli

  ```
  pip install eva-sub-cli
  ```

  Test the installation using help message

  ```
  eva-sub-cli.py -h
  ```

- Prepare a unique submission directory for each submission and do not reuse directories to avoid data loss during the validation process.
- Metadata files can be provided in either as spreadsheets or as JSON and passed using the option `--metadata_xlsx or --metadata_json.`
- Provide a VCF file and the reference FASTA can be provided using the `--vcf_files` and `--reference_data` options. It can also be provided directly in the metadata file directly.
- Use following commands for validation:

```
eva-sub-cli.py --metadata_xlsx metadata_spreadsheet.xlsx
--submission_dir submission_dir --tasks VALIDATE
```

For Docker users

```
eva-sub-cli.py --metadata_xlsx metadata_spreadsheet.xlsx
--submission_dir submission_dir --tasks VALIDATE --executor
docker
```

Note: For large VCF files, users can add `--shallow` argument to the command to validate only the first 10,000 lines in each VCF.

- After validation user can submit the vcf data and metadata using following command:

```
eva-sub-cli.py --metadata_xlsx metadata_spreadsheet.xlsx --submission_dir submission_dir --tasks VALIDATE
```

For docker users

```
eva-sub-cli.py --metadata_xlsx metadata_spreadsheet.xlsx --submission_dir submission_dir --tasks VALIDATE --executor docker
```

For validation as well as submission

```
eva-sub-cli.py --metadata_xlsx metadata_spreadsheet.xlsx \
                         --vcf_files vcf_file1.vcf vcf_file2.vcf --reference_fasta assembly.fa --submission_dir submission_dir
```

- Submission *via* email: Users can also email to eva-helpdesk@ebi.ac.uk to request submission and be required to follow the instructions sent by the EVA support team to complete the submission.

Step 6: Post-submission normalization, annotation, and statistical analysis at EVA:

All the non-human variants are accessioned by EVA including plants and EVA brokers the human variants to dbSNP. To make all the submitted data uniform post submission, EVA follows a few basic steps to normalize variants similar to dbSNP.

- Each variant shifted to left aligned.
- Each variant is processed to keep the start and end position specific to the variation only.

Details of the normalization process can be found here**.**

After the normalization process, variants are annotated using Ensembl's VEP.

The EVA defines allele frequency (AF) as the proportion of a specific allele at a genetic locus within a given population. AF values are study-specific, meaning the same variant can have different AFs across studies.

Step 7: Access the accessioned variants and its future use:

To access the accessioned variants at the EVA, you can use the following ways :

- **Web Browser Search**: Visit the EVA website at https://www.ebi.ac.uk/eva/ and use the search bar to look up variants by rsID, study accession (e.g., PRJEBxxxxxx), or species.
- **Study Browser**: Navigate to the "Browse EVA Studies" section to locate datasets by organism, study ID, or project title. You can download VCF files or explore annotated variant tables directly.
- **REST API**: EVA also offers a public REST API for programmatic access to variant data, enabling automated querying by rsID, genomic region, or gene.
- **FTP Access**: For bulk downloads, EVA provides access via FTP at ftp://ftp.ebi.ac.uk/pub/databases/eva/, where VCF files and metadata are organized by project.

Optional step A: Register and submit sequencing reads and samples

A.1 Register the study

Each submission must be associated with a pre-registered study and sample. Users can register to study (also called Project) via the Webin Submission Portal.

- Register your account at the Webin Submission Portal and login with your credentials.
- Click on the 'Register Study' button and fill in all required fields in the form and submit.
- After submission, a study will receive a study accession number (*ERP#####, PRJEB#####)* which will be needed for submitting genome assembly and raw reads. These accessions would be cited in publications involving the data.

Example Submission:

Release Date	2025-12-01 (Set to future date upto 2 years if data needs to remain private for a while)
Study Name	GenomeAssembly_ICMB123_India_2025
Will you provide functional genome annotation?	Yes
Short Descriptive Study Title	Draft Genome Assembly of Pearl Millet Cultivar ICMB 123
Detailed Study Abstract	This study presents the draft genome assembly of ICMB 123, a widely cultivated pearl millet variety in India. Sequencing was performed using Illumina HiSeq and Oxford Nanopore platforms, and hybrid assembly was generated using Flye and polished with Pilon. This dataset is expected to support future breeding and climate resilience research.
PubMed Citations	PMID: 37298320 (Add if already published; otherwise leave blank)
**Study Attributes:**	
Tag	Geographic location
Value	India
Tag	Target species

Value	Pennisetum glaucum
Locus Tag Prefixes (required only if you checked the box for functional annotation)	PMI (for *Pearl Millet India*)<br>(should contain only alphanumeric characters of 3 to 12 characters long, starts with a letter and all letters should be in uppercase)

A.2 Register a Sample

Samples represent the biological source of your sequencing data and are essential for organizing your reads, assemblies, or analyses. Here's how to register them easily using the Webin Submission Portal.

- Log in to your Webin Submission Portal and click on 'Register Samples'.
- Select "Download spreadsheet to register samples" to open the checklist menu.
- Browse and pick the appropriate checklist (e.g., "Plant and Fungi Sample Checklist").
- Customize your checklist by adding relevant fields and click 'Next' followed by downloading the TSV template spreadsheet.
- Fill out the template spreadsheet in excel or google sheets.

  See the example below:

scientific_name	Pennisetum glaucum
collection_date	2025-01-15
geographic_location	India: Rajasthan
isolate	ICMB 123
host	*None* (if not applicable)
environment_biome	*agricultural field*
description	*Pearl millet sample used for genome assembly*

- To submit the spreadsheet, go back to the 'Register Sample' section and click on 'Upload filled spreadsheet' to register samples. Upload the file and click 'Submit Completed Spreadsheet'. Upon successful upload you'll receive sample accession numbers (e.g., ERS####### and SAMEA#######).

A.3 Submit associated raw reads

- Go to the ENA Webin Submission Portal and click "Submit Reads."
- Expand "Download spreadsheet template for Read submission."
- Choose the appropriate file type for your data: Single-end FASTQ, Paired-end FASTQ, CRAM files, BAM files, HDF5 or FAST5 files
- Review the required and optional metadata fields (e.g., instrument model, insert size).
- Click "Next" and then download your customized .tsv spreadsheet template.
- Fill out the template spreadsheet.

See the example below:

sample	SAMEA12345678 (Your BioSamples accession)
study	PRJEB12345 (Your ENA Study accession)
instrument_model	Illumina HiSeq 2500
library_name	ICMB123_RNASeqLib1
library_source	GENOMIC (Permitted: GENOMIC, TRANSCRIPTOMIC, etc.)
library_selection	RANDOM (Permitted: RANDOM, PCR, RANDOM PCR, etc.)
library_strategy	WGS (Permitted: WGS, RNA-Seq, WXS, etc.)
library_layout	PAIRED
forward_file_name	ICMB123_R1.fastq.gz
forward_file_md5	1a79a4d60de6718e8e5b326e338ae533 (Generated via md5sum)
reverse_file_name	ICMB123_R2.fastq.gz
reverse_file_md5	4e07408562bedb8b60ce05c1decfe3ad (Generated via md5sum)

- Return to the 'Submit Reads Section' in the Webin Submission Portal, expand "Upload filled spreadsheet template for Read submission," use the Browse button or drag-and-drop your file, and click "Submit Completed Spreadsheet."

Note:

All reads must be de-multiplexed before submission.
Ensure that your files are already uploaded to your Webin FTP/SFTP account.

- If your metadata passes validation, you will immediately receive experiment (e.g., ERX123456) and run (e.g., ERR123456) accession numbers, after which ENA will check your uploaded read files and notify you of any errors via email, while allowing you to track submission progress through the Run Reports tab in the Webin Submission Portal. Link them in the RUN_REF field of the manifest file in *Step 2.4*.

Optional Step B: Submit a genome assembly to ENA

Genome assemblies can be submitted to ENA via Webin command line submission interface with '-context genome' option. The detailed submission instructions are available at Submitting Genome Assemblies of Individuals or Cultured Isolates — ENA Documentation 1 documentation.

1. *Refer to Optional Step A to submit the sample and raw reads associated with the genome assembly*
2. *Prepare the files for genome assembly:*

- Create the manifest file to specify the set of files that are part of submission. The file is identified in the Webin-CLI command using the -manifest <filename> option. The table summarises the list of files required based on assembly level:

**Assembly Types**	**Required Files**
Contig-Level	1 manifest file<br>1 FASTA or flat file<br>Example: GCA_000003085
Scaffold-Level	1 manifest file<br>1 FASTA **or** flat file<br>Optional AGP file (for scaffold structure)<br>Example: GCA_902705575
Chromosome-Level	1 manifest file<br>1 FASTA **or** flat file<br>1 chromosome list file<br>Optional: AGP + unlocalised list<br>Example: GCA_000237925

- Add the metadata and filenames as per the assembly type in the manifest file. This text file has only two columns 'Field name' and 'Field value' as depicted in the example below:

STUDY	PRJEB12345
SAMPLE	SAMEA123456
ASSEMBLYNAME	ICMB123_assembly_v1
ASSEMBLY_TYPE	clone or isolate
COVERAGE	80
PROGRAM	Flye v2.9 + Pilon
PLATFORM	Oxford Nanopore, Illumina
MOLECULETYPE (optional)	genomic DNA
FASTA (optional)	icmb123.fasta.gz
MINGAPLENGTH (optional)	200
DESCRIPTION	Draft genome of ICMB 123 pearl millet cultivar

- Install Webin-CLI from ENA docs. Please refer to the Webin command line submission interface documentation for full information about the submission process.
- Run the Webin-CLI validation command by specifying your credentials and path to your manifest file:

```
webin-cli -username Webin-XXXXX -password YYYYYYY -context genome -manifest manifest.txt -submit
```

- Now run the Webin-CLI submission command:

```
webin-cli -username Webin-XXXXX -password YYYYYYY -context genome -manifest manifest.txt -validate
```

- Upon successful submission genome assembly has been assigned with an analysis accession number (ERZxxxxxx) immediately on the interface. These are temporary accessions and should not be cited in publications.
- After the internal validation process ENA assigns stable public accessions to the assembly (GCA_123456789.1).

References

1. Cezard, T. et al. The European Variation Archive: a FAIR resource of genomic variation for all species. *Nucleic Acids Res.* **50**, D1216–D1220 (2022).

2. Beier, S., Thiel, T., Münch, T., Scholz, U. & Mascher, M. VCF specification and best practices for variant data interoperability. *VCFv4.3 Specification*. Samtools/HTS-specs (2022). https://samtools.github.io/hts-specs/VCFv4.3.pdf

3. Cingolani, P. et al. A program for annotating and predicting the effects of single nucleotide polymorphisms, SnpEff. *Fly* **6**, 80–92 (2012).

4. Danecek, P. et al. Twelve years of SAMtools and BCFtools. *GigaScience* **10**, giab008 (2021).

5. McLaren, W. et al. The Ensembl Variant Effect Predictor. *Genome Biol.* **17**, 122 (2016).

6. EMBL-EBI. European Variation Archive (EVA) Submission Portal. https://www.ebi.ac.uk/eva/?Submit-Data (2025).

7. EMBL-EBI. EVA Submission CLI (eva-sub-cli). GitHub repository. https://github.com/EBIvariation/eva-sub-cli (2025).

8. EMBL-EBI. VCF Validator Tool. GitHub repository. https://github.com/EBIvariation/vcf-validator (2025).

9. EMBL-EBI. ENA Webin Submission Portal. https://www.ebi.ac.uk/ena/submit/webin/login (2025).

10. EMBL-EBI. Submitting Genome Assemblies of Individuals or Cultured Isolates. ENA Documentation. https://ena-docs.readthedocs.io/en/latest/submit/assembly/genome.html (2025).
